# As^III^ Selectively Induces a Disorder-to-Order Transition in the Metalloid Binding Region of the AfArsR Protein

**DOI:** 10.1021/jacs.3c11665

**Published:** 2024-05-31

**Authors:** Annamária Tóth, Kadosa Sajdik, Béla Gyurcsik, Zeyad H. Nafaee, Edit Wéber, Zoltan Kele, Niels Johan Christensen, Juliana Schell, Joao Guilherme Correia, Kajsa G. V. Sigfridsson Clauss, Rebecca K. Pittkowski, Peter Waaben Thulstrup, Lars Hemmingsen, Attila Jancsó

**Affiliations:** † Department of Molecular and Analytical Chemistry, University of Szeged, Dóm tér 7-8, H-6720 Szeged, Hungary; ‡ Department of Medical Chemistry, University of Szeged, Dóm tér 8, H-6720 Szeged, Hungary; § HUN-REN-SZTE Biomimetic Systems Research Group, Dóm tér 8, H-6720 Szeged, Hungary; ∥ Department of Chemistry, Faculty of Science, University of Copenhagen, Thorvaldsensvej 40, 1871 Frederiksberg C, Denmark; ⊥ Institute for Materials Science and Center for Nanointegration Duisburg-Essen (CENIDE), University of Duisburg-Essen, 45141 Essen, Germany; # Centro de Cięncias e Tecnologias Nucleares, Departamento de Engenharia e Cięncias Nucleares, Instituto Superior Técnico, Universidade de Lisboa, 2695-066 Bobadela LRS, Portugal; ∇ European Organization for Nuclear Research (CERN), CH-1211 Geneva, Switzerland; ○ MAX IV Laboratory, Lund University, P.O. Box 118, SE-221 00 Lund, Sweden; ◆ Department of Chemistry, University of Copenhagen, Universitetsparken 5, 2100 Kobenhavn Ø, Denmark

## Abstract

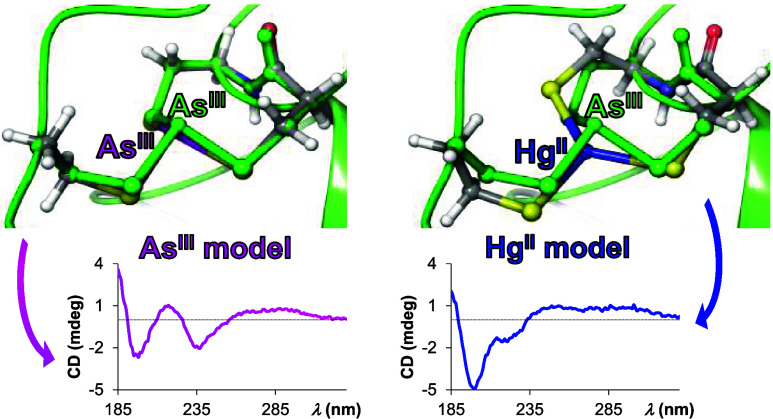

Arsenic is highly toxic and a significant threat to human health, but certain bacteria have developed defense mechanisms initiated by As^III^ binding to As^III^-sensing proteins of the ArsR family. The transcriptional regulator AfArsR responds to As^III^ and Sb^III^ by coordinating the metalloids with three cysteines, located in a short sequence of the same monomer chain. Here, we characterize the binding of As^III^ and Hg^II^ to a model peptide encompassing this fragment of the protein via solution equilibrium and spectroscopic/spectrometric techniques (pH potentiometry, UV, CD, NMR, PAC, EXAFS, and ESI-MS) combined with DFT calculations and MD simulations. Coordination of As^III^ changes the peptide structure from a random-coil to a well-defined structure of the complex. A trigonal pyramidal AsS_3_ binding site is formed with almost exactly the same structure as observed in the crystal structure of the native protein, implying that the peptide possesses all of the features required to mimic the As^III^ recognition and response selectivity of AfArsR. Contrary to this, binding of Hg^II^ to the peptide does not lead to a well-defined structure of the peptide, and the atoms near the metal binding site are displaced and reoriented in the Hg^II^ model. Our model study suggests that structural organization of the metal site by the inducer ion is a key element in the mechanism of the metalloid-selective recognition of this protein.

## Introduction

Organic and inorganic arsenic compounds in any of their regular oxidation states (+3, + 5, or −3) display toxicity to all lifeforms.^1−6^ Arsenic compounds have been proposed to interfere with numerous biochemical processes of living cells by, e.g., inhibiting enzymes (pyruvate dehydrogenase (PDH), thioredoxin (Trx), DNA repair enzymes, etc.),^7^ substituting phosphates,^8^ and playing roles in the generation of reactive oxygen species, leading to oxidative damage of biomolecules.^4−6^ It is widely accepted that the molecular mechanism of action of trivalent arsenicals involves the interaction of As^III^ with the thiol groups of proteins and enzymes or their cofactors.^7,9^ The inhibition of the PDH and α-ketoglutarate dehydrogenase (KGDH) enzyme complexes was proposed to be related to the binding of arsenous acid and monomethylarsonous acid to the reduced form of lipoic acid.^10^ Direct interaction of trivalent arsenicals with the C3H or C4 motifs of zinc finger proteins (e.g., Fpg (formamidopyrimidine DNA glycosylase), XPA (xeroderma pigmentosum group A), PARP-1 (poly(ADP-ribose) polymerase-1)) was suggested to inhibit DNA repair processes.^11,12^ The binding of various arsenicals to the CGPC peptide segment of Trx was also demonstrated and characterized by different mass spectrometry techniques.^13^

Inorganic arsenic(III) (*i*As^III^), i.e., arsenous acid (or arsenite) in aqueous solutions, may form one, two, or three As–S bonds via condensation reactions. Kitchin et al. performed systematic studies on the interaction of arsenous acid with cysteine-containing peptides bearing 0–4 cysteine residues^14^ and with peptides displaying two cysteines separated by 0–17 intervening amino acids.^15^ These experiments provided basic information both about the binding affinity of arsenous acid to mono-, di-, and trithiol binding sites and to separated thiols modeling As^III^ coordination to more than one ligand, as well as about the association/dissociation kinetics of these binding schemes.^16^ The relatively high *i*As^III^-binding affinity of di- and trithiols indicates their greater biochemical relevance, as compared to monothiol coordination.^16^ There is only a slight extra stabilization promoted by the coordination of a third thiol sulfur donor as compared to dithiolate As^III^ complexes. However, tricoordinated sites may be more common in As^III^-binding proteins. Three cysteine-coordinated metalloid centers were identified, e.g., in zinc finger proteins,^12,17^ metallothioneins,^18^ the metallochaperone ArsD,^19^ the As^III^/Sb^III^ translocating ATPase ArsA,^20^ the key enzyme in arsenic biomethylation S-adenosylmethionine methyltransferase,^21^ or the metalloid-responsive bacterial metalloregulator ArsR.^22−26^

Specific proteins classified in the large ArsR protein family^27^ play a key role in controlling arsenic (or antimony) resistance in bacterial cells through transcription regulation.^27−29^ The binding affinities of *Escherichia coli* R773 ArsR,^22^ AfArsR^23^ (from *Acidithiobacillus ferrooxidans*), CgArsR^24^ (from *Corynebacterium glutamicum*), and CviArsR (from *Chromobacterium violaceum*)^30^ for As^III^ (or Sb^III^) were found to be in the *K*_d_ = 10–150 μM range for As^III^ (and *K*_d_ = 2–10 μM for Sb^III^). While the selective responses of these ArsR proteins have rarely been tested in *in vitro* studies of the bare proteins, just as the metalloid selectivity of AfArsR against Cd^II^ in promoting the dissociation of the protein from the regulated DNA,^23^ biosensing assays showed the efficiency of As^III^ and Sb^III^ to selectively promote the transcription of reporter genes.^31−36^ However, the molecular details of the metalloid recognition mechanism of ArsR proteins are still largely unresolved.

Designed three-stranded coiled-coil oligopeptidic constructs offering three Cys thiols for metal(loid) binding, as model systems, were investigated by Pecoraro et al.^37−40^ As^III^ stabilized the formation of the α-helical three-stranded structures, by forming three As–S_Cys_ bonds, under conditions where the two-stranded coiled-coil conformation is favored in the absence of metal ions.^37^ The X-ray structure of a three-stranded Cys-containing Coil Ser (L9C)_3_ showed that the three Cys residues are locked into their interior conformation by As^III^, which is located at the same side of the three-atom sulfur plane as all three Cys Cβ carbons,^38^ mimicking the conformation of the ArsR metal centers.^25^ It is proposed that As^III^ binding in this all-*endo* trigonal pyramidal geometry is stabilized by the optimized Cys rotamers, forming a cavity that is ideal for the small As^III^ with its short-length As–S bonds.^38^ Importantly, there is space below the As center for the As lone pair; thus, steric hindrance by bulky hydrophobic residues from the second coordination sphere is minimized.^39,40^ Such a small cavity is nonideal for Pb^II^, a soft metal ion also having a lone electron pair but a different size and charge.^40^ It was also proposed that the hydrophobic layers above and below the 3-Cys planes influence the shape and stability of trigonally coordinated Hg^II^ structures, manifested in a difference in the p*K*_a_ values of the third coordinating thiols.^39^ As^III^ binding by two tripodal pseudopeptides also demonstrated the preferred formation of the all-*endo* conformers, mimicking well the structure of ArsR As^III^-centers.^41^ DFT studies indicated not only the importance of the proper cavity size, accommodating the As lone pair, but also the role of electrostatic interactions and H-bonding in the second coordination sphere of As^III^ in stabilizing the energetically favored structures.

**Figure 1:**
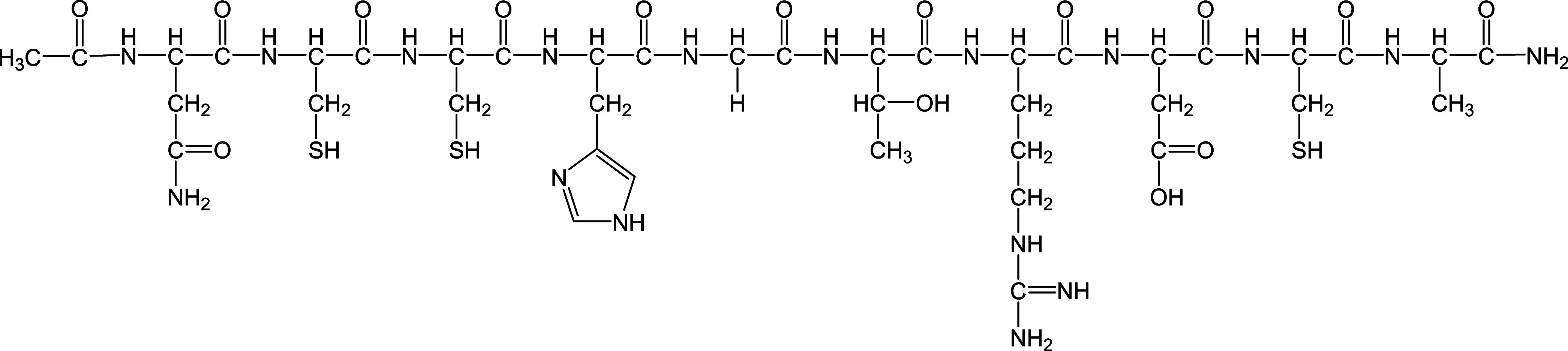
Schematic structure of the studied ligand, Ac-NCCHGTRDCA-NH_2_ (**L**).

Here, we present spectroscopic and theoretical studies with the oligopeptide Ac-NCCHGTRDCA-NH_2_ (**L**) (Figure 1), encompassing the effector binding fragment of the ArsR metalloid regulator from *A*. *ferrooxidans*. The AfArsR protein is particularly interesting for potential biotechnological applications since it is the only ArsR family metalloregulator with a known crystal structure where the three cysteine residues forming an AsS_3_ site are located within a short sequence at a single monomer chain,^25^ in contrast to, e.g., CgArsR^25^ and several other dimeric metalloid regulators where the coordinating donor ligands are recruited from two monomers. As^III^ and Hg^II^ interaction with the model peptide is explored aiming to characterize As^III^ binding to the metalloid site in solution and to elucidate if differences between As^III^ and Hg^II^ binding may indicate how the selective response for As^III^ is achieved.

## Results and Discussion

### Titrations of the Ligand by As^III^ and Hg^II^ – UV Absorption and CD

**Figure 2:**
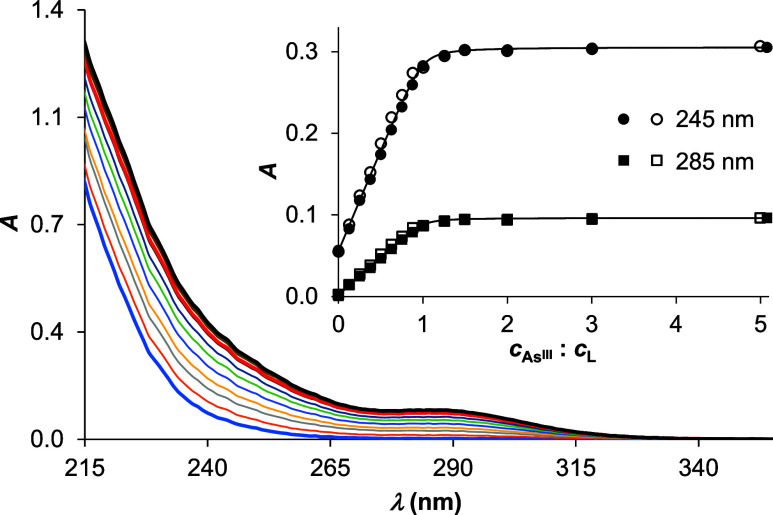
UV spectra recorded at different *c*(As^III^):*c*(**L**) ratios at pH 7.5. The thick lines are highlighted spectra for 0.0 (blue), 1.0 (red), and 5.0 equiv (black) of As^III^ per ligand. The inset shows the trend in change in absorbances at 245 and 285 nm using data from two independent measurements (symbols) and the fit (line) of data by the model As^III^ + **L** ⇌ As**L** (*c*(**L**) = 50.0 μM).

The interaction of arsenous acid (As^III^) and the peptide (Figure 1) was studied at different pH values by recording the UV-absorption spectra of the ligand at gradually increasing As^III^:**L** concentration ratios (Figures 2 and S1). The gradually increasing absorption between 215 and 330 nm is associated with S^–^ → As^III^ charge transfer bands, similar to the transitions observed for the binding of As^III^ by simple mono-,^42^ bis-,^42−44^ or tristhiol^41^-type compounds. While these bands usually appear as smooth or more definite shoulders, in the spectra recorded for the present system, there is a clearcut local absorption maximum slightly below 290 nm. The observed λ_max_ value (= 287 nm) and the intensity of this band (ε_287 nm_ = 1.91 × 10^3^ M^–1^ cm^–1^) unambiguously reflect three As^III^-bound thiolates^41,42^ and are clearly distinct from the less intense transitions characteristic for species with two thiolates.^42−44^ The trends of the spectroscopic features (Figures 2 and S1) suggest the formation of the same As^III^–peptide complexes between pH 2.0 and 7.5 and binding of one As^III^ per peptide.

Evaluation of the UV-monitored As^III^ titrations of **L** allowed determining the As^III^ binding affinity of the peptide. The fitting of data obtained at four different pH values (pH 2.0, 4.5, 6.0, 7.5; Figures 2 and S1), executed by the PSEQUAD software,^45^ reflects a slightly increasing stability with pH increase and an As^III^ binding affinity of the peptide under the neutral condition: log*K*^pH7.5^ = 6.35(3) falling slightly above the upper end of the range reported for multiple Cys-containing oligopeptides.^14,15^ A comparative analysis of stabilities and the details of calculations are presented in the Supporting Information.

**Figure 3:**
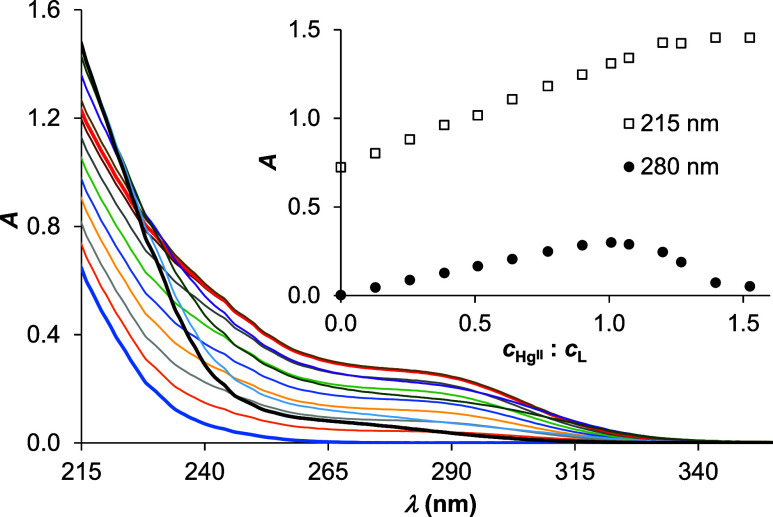
UV spectra recorded at different *c*(Hg^II^): *c*(**L**) ratios at pH 7.5. The thick lines are highlighted spectra for 0.0 (blue), 1.0 (red), and 1.5 equiv (black) of Hg^II^ per ligand. The inset shows the trend in the change of absorbances at 215 and 280 nm (*c*(**L**) = 40.0 μM).

Titration of the peptide by Hg^II^ also results in an initial increase of absorbances between 215 and 330 nm, but the trend in the evolution of spectra observed up to 1:1 Hg^II^:**L** concentration ratios are clearly different at pH 7.5 (Figure 3) and pH 2.0 (Figure S2). This is also demonstrated by the pH-dependent series of spectra recorded for the *c*(Hg^II^):*c*(**L**) 1:1 system (Figure S3). A broad shoulder, centered around 275–280 nm, develops upon adding 1 equiv of Hg^II^ per ligand at pH 7.5, closely resembling the S^–^ → Hg^II^ charge transfer bands observed for the binding of Hg^II^ to various peptides with a {HgS_3_} coordination mode.^46,47^ The same tristhiolate donor group environment of the metal ion is further supported by the observed molar absorbances (ε_280 nm_ = 7.77 × 10^3^ M^–1^ cm^–1^; ε_240 nm_ = 1.60 × 10^4^ M^–1^ cm^–1^). These values are in accordance with those reported for Hg^II^–peptide complexes with the three Cys units in different (CXCXC or CXCXXC) patterns but are also close to the molar absorbances of the {HgS_3_} species formed with NTA-based tripodal compounds^48,49^ bearing Cys or d-Pen arms or with three-stranded coiled coils.^37,50^ Under excess of Hg^II^ over **L**, the broad shoulder collapses, but in parallel, intensities in the low-wavelength regime remarkably increase up to a 1.5:1 Hg^II^:**L** concentration ratio, potentially reflecting species with a 3:2 Hg^II^:**L** composition, as found also for other short 3-Cys-containing peptides.^46,47^ Larger Hg^II^-excess resulted in unreliable absorbance readings, possibly due to aggregation processes or precipitate formation. The detected transformation of spectra between 1.0 and 1.5 equiv of Hg^II^ per ligand, leading to a weak absorption above ∼240 nm but an absorbance increase below ca. 225 nm, is in accord with the conversion of the {HgS_3_} species into a structure with Hg^II^ ions coordinated in a {HgS_2_} environment.^46−54^

At pH 2.0, the shape of the spectra and the absence of notable absorption above 240 nm up to 1.0 equiv of Hg^II^ per **L** clearly suggest that only two of the Cys-thiolates are bound to Hg^II^ (Figure S2). This is further corroborated by a pH-dependent series, obtained at a 1:1 Hg^II^:**L** ratio, indicating that a deprotonation process takes place with a p*K*_a_ ∼ 6.43, as fitted to the sigmoidal plots of absorbance traces, driving the {HgS_2_}-type species into a {HgS_3_} complex dominating at pH 7.5 (Figure S3). Previous studies with various ligands, offering three Cys thiol groups for Hg^II^ coordination, all showed that a transformation from bisthiolate to tristhiolate binding modes occurs in the slightly acidic/neutral pH-regime; however, the observed p*K*_a_ values span a rather broad range (from ∼4.3^46^ to 8.6^37^). Several parameters, including the position of the donor groups,^46,47^ coordination geometry around the metal ion center,^39^ the presence or absence of nearby bulky and/or electron-withdrawing/-donating substituents,^48,49^ and the net charge at the metal center, influence these p*K*_a_ values. Some of the studied cyclic peptides seem to provide the highest stabilization of the tristhiolate-bound Hg^II^ center, ultimately resulting in low p*K*_a_ values, while {HgS_3_} coordination within the interior of three-stranded coiled coils with Cys residues at the so-called “d positions” is the least favored.^37,39,55^ The {HgS_2_}-type complexes of short linear peptides transform into {HgS_3_} species with p*K*_a_ values of ca. 5.0,^46,47^ which notably differ from the data observed in the studied system (p*K*_a_ ∼6.4), suggesting that the positions (^2^Cys, ^3^Cys, ^9^Cys) of the three Cys residues in the metal binding fragment of AfArsR are not ideal for stabilizing the tristhiolate coordination environment over the {HgS_2_} structure.

The Hg^II^ binding affinity of **L** was also determined by combining data of a peptide displacement reaction with iodide ions (followed by UV spectroscopy, Figure S4) and pH-potentiometric titrations (Figure S5). As expected, the apparent stability obtained for the Hg^II^-**L** species (log*K*^pH7.5^ = 39.0) reflects a huge difference in favor of Hg^II^ binding compared to As^III^. These studies are described in the Supporting Information.

**Figure 4:**
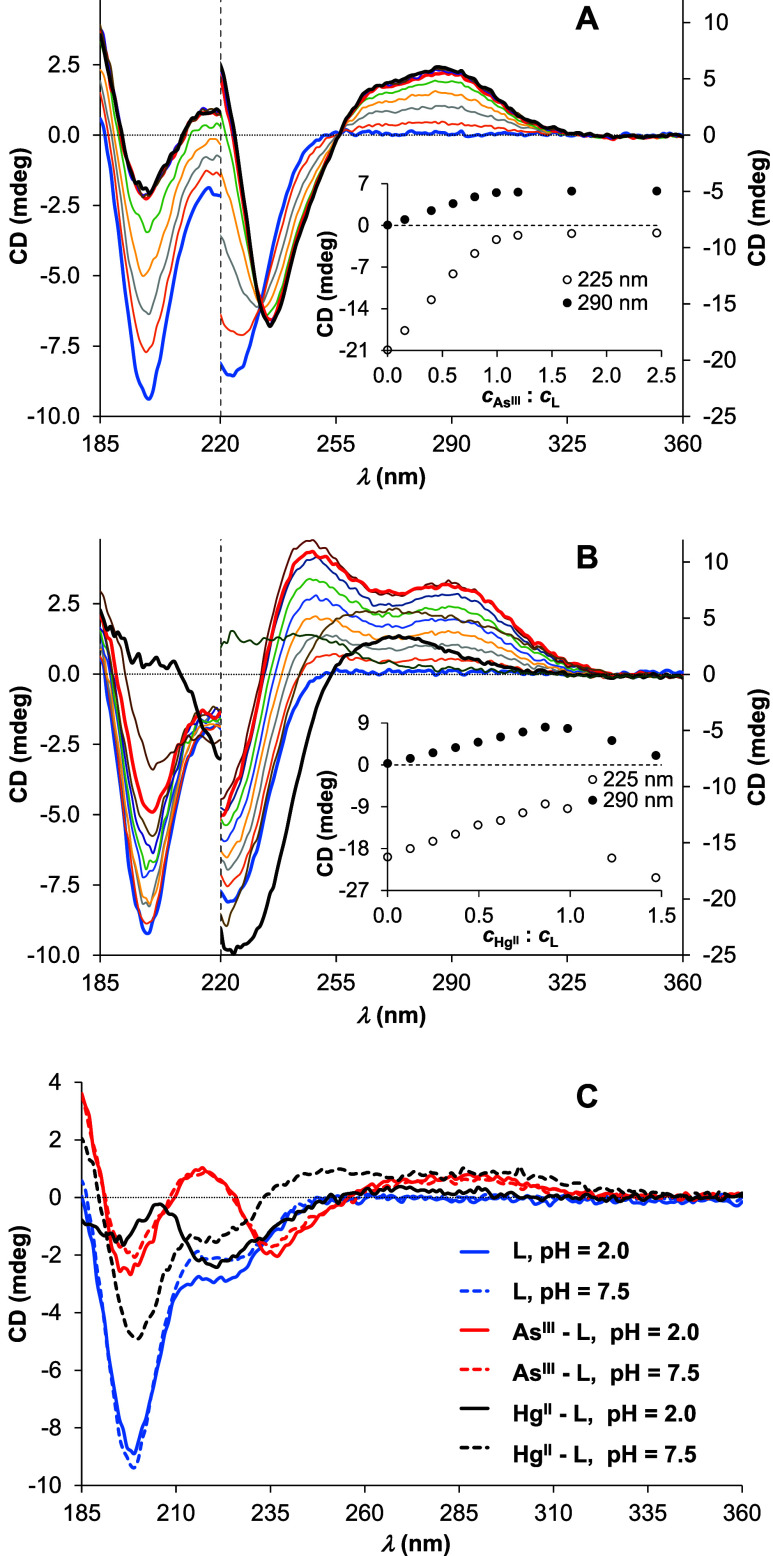
CD spectra recorded at different *c*(As^III^):*c*(**L**) (A) and *c*(Hg^II^): *c*(**L**) (B) ratios at pH 7.5. Presented data are combined from experiments performed under different conditions: data below 220 nm (left axes) were obtained in a 0.1 mm cylindrical cuvette with *c*(**L**) = 650 μM, while data above 220 nm (right axes) were measured in a 1.0 cm conventional cell with *c*(**L**) = 65.0 μM. (Full-range spectra measured in the cylindrical cuvette are presented in the Supporting Information, Figure S6.) The thick lines are highlighted spectra for 0.0 (blue), 1.0 (red), and 2.5 or 1.5 equiv (black) of As^III^ (A) or Hg^II^ (B) per ligand. The insets show the trend in the change of CD intensities at 225 and 290 nm. Panel (C) compares the CD spectra obtained without metal ions and with 1.0 equiv of As^III^ and Hg^II^ at pH 2.0 (solid lines) and 7.5 (dashed lines) with *c*(**L**) = 650 μM, path length = 0.1 mm.

Interaction of As^III^ and Hg^II^ with the AfArsR model peptide at pH 7.5 and 2.0 was also followed by circular dichroism (CD) spectroscopy (Figures 4 and S6). The trends in the change of CD intensities at selected wavelength values, accompanying the titration of **L** by either As^III^ or Hg^II^, perfectly correlate with the observed trends in the UV-monitored titrations. The recorded ellipticities follow a saturation-like tendency and level off slightly above 1.0 equiv of As^III^ per ligand at both pH values, and the observed isodichroic points at ∼232 and 257 nm demonstrate a simple equilibrium between the free ligand and a single complex species.

A clear breakpoint near 1.0 equiv of Hg^II^ reflects a stronger binding to the ligand, as compared to As^III^, as well as a shift in the speciation between 1:1 and 1.5:1 Hg^II^:**L** concentration ratios from monomeric toward multinuclear structures. The series of spectra display information about both the changes in the secondary structure of the peptide via As^III^ or Hg^II^ binding and the coordination mode of the ligand. The free peptide displays an intense negative Cotton effect (∼198 nm) with a lower energy shoulder, attributed to π–π* and n–π* transitions of the amide bond,^56,57^ reflecting a disordered structure.^58−60^ This random-coil signature completely diminishes as a consequence of binding of As^III^ to **L** (Figures 4A,C and S6A,B), indicating the organization of the peptide skeleton into a more structured form. Moreover, the same CD signal and thus the same fold of the peptide are observed at both pH 2.0 and 7.5 for the 1:1 As^III^:**L** complex. The CD signature of the As^III^-bound ligand, i.e., a combined CD minimum–maximum–minimum at 198, 216, and 236 nm, respectively, is very similar to the spectrum of the Cd^II^ complex of the (Glu–Cys)_4_–Gly phytochelatin peptide.^61^ Based on this CD pattern, the binding of As^III^ to **L** presumably enforces different turns, such as α-helix and 3_10_ helix-like turns or β-turns in the peptide backbone.^61−63^

The CD spectra recorded with 1.0 equiv of Hg^II^ at pH 2.0 and 7.5 (Figure 4C) demonstrate that the peptide adopts a remarkably different structure compared to its As^III^-bound state, indicating that Hg^II^ fails to fold the peptide into the structure of the As^III^:**L** complex. Surprisingly, at pH 7.5, the CD spectrum of the 1:1 Hg^II^:**L** complex exhibits rather similar features as the (disordered) free peptide, implying that the complex remains, at least partially, disordered. These conclusions are based on the assumption that the CD signal from the peptide backbone chromophore dominates over charge transfer bands involving Hg^II^ and As^III^ in the short wavelength range of the spectra, *vide infra*.

The right side of panels A and B in Figure 4 (from the vertical dashed lines) highlights the near-UV region of the recorded spectra by presenting experiments performed in a long path length cell (1.0 cm). The positive CD bands appearing at ≥240–250 nm are related to charge transfer (S^–^ → As^III^ or S^–^ → Hg^II^ LMCT) transitions being chirally perturbed by the peptide. Charge transfer-related CD bands are relatively rarely reported in metal ion–peptide systems,^37,47^ probably because of their typically weaker intensity as compared to the peptide backbone-related transitions. Nevertheless, as demonstrated by the present example, such bands can be rather prominent in samples of short-length peptides if they are centered in the wavelength regime that do not overlap with the ligand transitions but may also modulate the observed signals at higher energies, i.e., below ∼210 nm. Furthermore, in contrast to the well-defined signs (positive or negative) of the CD signals related to the different secondary structural elements of the peptide backbone, literature data suggests that the LMCT-induced transitions may result in negative or positive signals, depending on the type of ligand, the number/configuration of chirality centers, and their relative positions to the thiolate–metal ion bonds.^34,41,47,49,64^ The evolution of the low-energy, CT-related CD bands in the As^III^-**L** system at any studied pH (Figures 4A and S6A,B) and with Hg^II^ at pH 7.5 (Figures 4B and S6D) provides strong support for the formation of the previously proposed tristhiolate binding mode of **L**. Indeed, the absence of such features in the Hg^II^-**L** sample at pH 2.0 (Figure S6C) is in accord with the higher energy of the LMCT bands in {HgS_2_}-type species, shifting also the related CD signals into the wavelength range of the amide-bond transitions.

**Figure 5:**
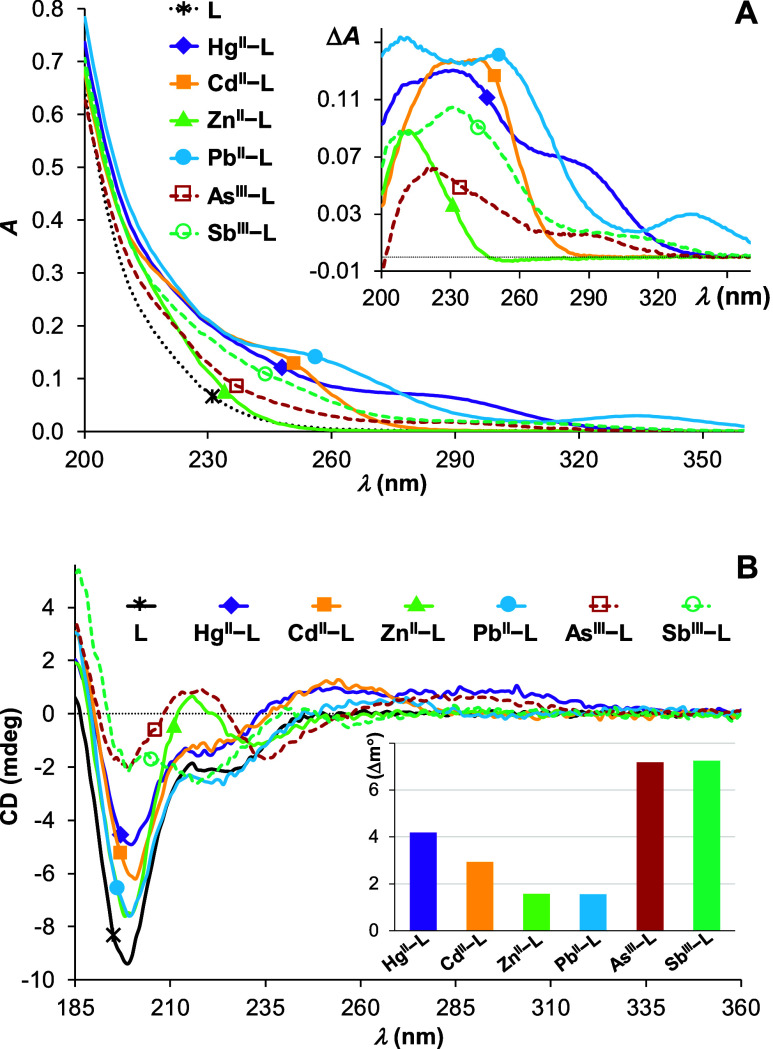
Comparison of UV spectra (A) and CD spectra (B) recorded in different M^II^-**L** and M^III^-**L** samples (M^II^: Hg^II^, Cd^II^, Zn^II^ or Pb^II^; M^III^: As^III^ or Sb^III^) at pH 7.5 (phosphate buffer, *c* = 20 mM). (A) UV spectra were recorded using c(M^II^):c(**L**) 1:1 and c(M^III^):c(**L**) 2:1 concentration ratios. Semimetals were used in excess to ensure complete transformation of the ligand to the semimetal-bound species. The inset presents difference spectra obtained by subtracting the spectrum of the unbound ligand, recorded at the same concentration, from the spectra of the metal ion-containing samples (*c*(**L**) = 10.0 μM, path length = 1.0 cm). (B) CD spectra were recorded at equimolar ratios of all of the components in all systems. The inset shows the metal ion-induced change of CD intensities at 200 nm, as compared to the intensity of the CD spectrum recorded for the free ligand (*c*(**L**) = 650 μM, path length = 0.1 mm).

Considering the short length of the AfArsR model peptide, we cannot exclude that the CD features below 240–250 nm are also affected by LMCT signatures. In general, the relative intensity of the peptide-related transitions and LMCT contributions to the CD of low-molecular-weight metal complexes is an unresolved and open question. With an aim to elucidate the influence of LMCT-related contributions to the observed CD pattern at higher energies, especially, near the negative CD signal (∼199 nm) characteristic for the unbound peptide, we compared the UV-absorption spectra as well as the CD spectra of a number of M^II^/M^III^–peptide systems in the high-energy wavelength regime (M^II^: Hg^II^, Cd^II^, Zn^II^, and Pb^II^; M^III^: As^III^, Sb^III^—in the form of its tartrate complex to hinder the hydrolysis of the metalloid^65^) (Figure 5). All of these (semi)metal ions have well-known strong affinities to multithiol ligands and are expected to be bound efficiently by the studied peptide displaying three cysteine residues.^51,65−67^ The peptide was expected to fully displace the Sb^III^-bound tartrate, according to previous studies describing the complete exchange of the Sb^III^-coordinated tartrate by a presumably less efficient monothiol ligand, glutathione.^68^ Metal ion titration series demonstrated efficient binding of all of the studied ions to the peptide under the applied conditions. The UV data shown in Figure 5A indicate that the effect of any of the metal ions on the absorbance of the free ligand around 200 nm, especially that of As^III^, is not particularly strong.

In contrast, the observed magnitudes of the CD spectra around 200 nm are rather different for the different ions (Figure 5B). All of the divalent ions, even Pb^II^ bearing a lone electron pair, induces a significantly smaller change in the intensity of the CD signal centered at ∼199 nm, as compared to the effect of As^III^ or Sb^III^. Although the influence of the coordinating (semi)metal ions on the CD of such a short peptide is clearly rather complex, the observed effects, solely at higher energies, i.e., near 200 nm, may still imply that the two semimetals shape the peptide in a more defined conformation, as compared to the divalent metal ions, whereas the contributions of LMCT to the CD spectra at longer wavelengths (>220 nm) are substantial. The question of peptide backbone-related transitions to the recorded CD spectra was also explored by semiempirical approaches and TD-DFT calculations leading to similar, but not conclusive, results. The applicability of these methods for low-molecular-weight metal ion–peptide complexes needs further investigation; nevertheless, we have added our attempts to the Appendix of the Supporting Information for interested readers.

The distinctly different conformations that the peptide adopts in its As^III^- and Hg^II^-bound states, based on the CD data, may be related to the different coordination geometry preferences of As^III^ and Hg^II^. The lone electron pair of As^III^ restricts the metalloid into a trigonal pyramidal geometry, whereas Hg^II^ is more likely to accommodate a trigonal planar or a distorted trigonal environment. Indeed, it is interesting that the peptide model of the metalloid site of AfArsR, even without the surrounding protein fold, shapes into clearly distinguishable ensembles of conformations upon binding the different ions, and we speculate that this may have a relevance in the protein’s metalloid-selective function.

### ESI-MS and ^1^H NMR Characterization of the Metal(loid) Binding of L

ESI-MS spectra provide further support for the speciation models, proposed by UV and CD spectroscopies and pH potentiometry (Supporting Information) for the As^III^-**L** and/or Hg^II^-**L** systems, along with information about possible binding isomers of the {HgS_2_}-type species present under acidic conditions. These data with representative figures (Figures S7–S9) are presented in the Supporting Information.

**Figure 6:**
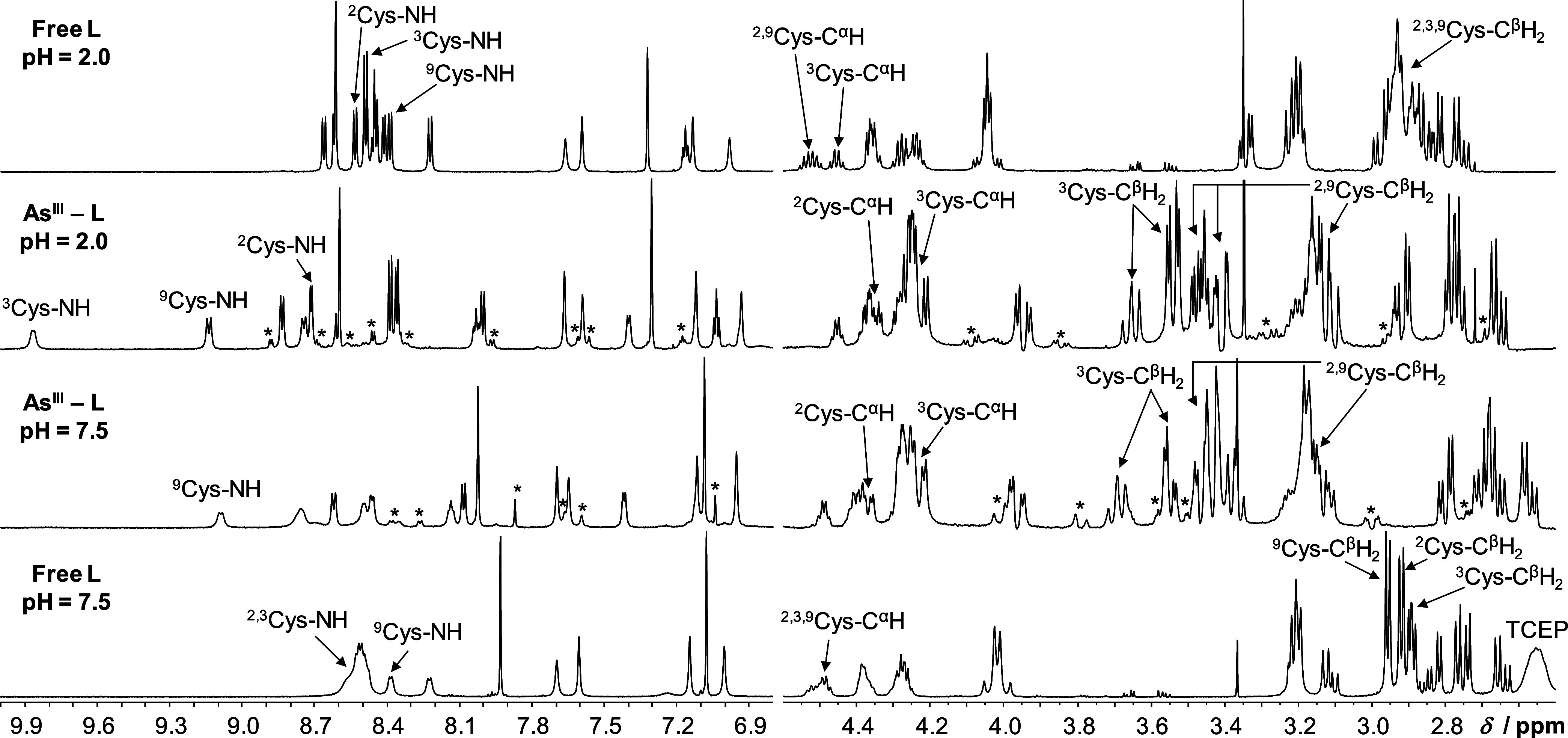
Parts of the ^1^H NMR spectra of **L** in the absence and presence of 1.0 equiv of As^III^, recorded in H_2_O/D_2_O 90/10 V/V % at pH 7.5 (bottom) and 2.0 (top) and assignment of the Cys residue-related resonances (*T* = 282 K, *c*(**L**) = 1.0 mM). Note that the free ligand sample at pH 7.5 contained the reducing agent TCEP (signals of P-CH_2_ and CH_2_-COO^–^ appear at ∼2.55 and ∼2.30 ppm, respectively) to maintain the reduced (thiol) form of the peptide (*c*(TCEP) = 1.0 mM). Representative resonances of minor isomers present in As^III^-**L** are marked by * symbols (see also Tables S6 and S7). The sharp peak between 3.3 and 3.4 ppm is due to a small amount of methanol.

Full assignment of the ^1^H NMR resonances of the free peptide was done, via 1D and 2D NMR experiments (Experimental Procedures in the Supporting Information), at pH 7.5 and 2.0 (Tables S4 and S5 and Figure S10) at 282 K, resulting in significantly better resolved spectra, as compared to those recorded at 298 K, especially at pH 7.5. Spectra obtained for samples with a 1:1 As^III^:**L** concentration ratio at either pH 7.5 or 2.0 (Figure 6) demonstrate that the presence of As^III^ induces significant changes in the chemical shifts and the splitting pattern of the peptide both in the aliphatic (ca. δ < 4.7 ppm) and in the amide region (ca. 6.8 < δ < 9.5 ppm). Signals of nearly all amino acids are notably affected, but the observed new resonances are only slightly broadened and can be assigned to the same dominant species at both pH values (Tables S6 and S7). The chemical shifts of the different protons change very little with pH with the exception of those that are notably affected by the change of the protonation state of nearby noncoordinating residues. This indicates that the binding of all three Cys-thiolates to As^III^ affects the structure of the whole ligand. The sharp lines imply that either As^III^ binding “locks” the peptide into a well-defined structure or that several structures are present in a fast exchange on the NMR time scale. Our previous ^1^H NMR results, obtained in samples of *i*As^III^ and bis- or tristhiol ligands, all reflected slow exchange between different As^III^ complexes and/or the free ligand when such species were in equilibrium.^41,43,44^ In contrast, ^1^H NMR experiments following the addition of the monothiol ligands cysteine and glutathione to As^III^ showed a relatively fast (Cys) and intermediate (GSH) exchange rate between the free and bound forms.^69^ This difference demonstrates that the “origin” of the multiple As–S bonds, whether they form with the same molecule (even if the chelate size is large) or with separate ligands, affects, besides stability, the kinetics of *i*As^III^ complexation.

It is not surprising that the most severely affected resonances are those of the Cys^β^H_2_ protons that are all shifted downfield by several tens of ppm by As^III^ binding. Besides, the NH (amide) signals of the Cys residues also appear at notably higher chemical shifts compared to the unbound peptide. A careful inspection reveals that small additional resonances for almost all groups of protons, besides those attributed to the complexed peptide, also emerge in the spectra of the As^III^-**L** samples (see Figures 6 and S11). Most of these are also fundamentally shifted, as compared to the signals of the free ligand, implying that the ligand is indeed present in (at least) two complexed isomeric forms, and one of these isomers is significantly more populated than the other(s).

^1^H–^1^H ROESY and NOESY spectra demonstrate several medium- and long-range NOE interactions (Figure S12) that could not be observed in the unbound peptide, displaying a random-coil structure. The contacts indicate a turn-like orientation of the central part of the peptide in the presence of As^III^, which is a consequence of the tristhiolate-type coordination of the As^III^ center.

The presence of Hg^II^ also has a strong impact on the ^1^H NMR spectra of **L** (Figure S13); however, the signals are severely broadened, providing very little direct information about the binding modes of the peptide. Both at pH 7.5 and at pH 2.0, all three Cys^β^H_2_ resonances seem to be shifted downfield (Figure S13). At pH 7.5, it is in line with the proposed tristhiolate binding mode of the peptide. Indeed, at this pH, a small fraction of the ligand is found in a {HgS_2_}-type species (see Figure S5B), and an exchange between the {HgS_2_} (isomers) and the {HgS_3_} complex, at an intermediate rate on the NMR time scale, may partially explain the large line widths.^70^ The role of chemical exchange is further corroborated by the observed gradual shifting of the Cys^β^H_2_ signals along with the increasing Hg^II^ content from 0 to 0.5 and finally to 1.0 equiv per ligand (Figure S13). Besides, the presence of a small amount of other species, i.e., trinuclear complexes, or internal conformational exchange dynamics within the monocomplex(es) might also contribute to line broadening.

Based on our previous experiences with systems of Hg^II^ and short, two-Cys-containing oligopeptides,^53,71−74^ we assumed that the presence of a uniform {HgS_2_} structure at pH 2.0 would result in a well-resolved ^1^H NMR spectrum with clearly assignable resonances. The predominance of one of the possible {HgS_2_} isomers, especially the one with an unbound ^9^Cys thiol (suggested under ESI-MS conditions; see the Supporting Information), might leave one of the Cys^β^H_2_ signals unaffected or only weakly affected. As it turned out, the spectrum recorded at pH 2.0 displays broad signals for all amide protons, the aliphatic protons of Cys, and several other residues (Figure S13). This implies that the {HgS_2_}-type complex presumably exists in the three possible isomeric forms and exchanges the thiolates with the rate falling into an intermediate regime on the NMR time scale.^70^ Furthermore, the presence of a small fraction of trinuclear species at a 1:1 c(Hg^II^):c(**L**) ratio cannot be excluded at the concentration of the NMR experiments.

These data, again, suggest fundamental differences in the As^III^- and Hg^II^-binding characteristics of the model peptide. While As^III^ is accommodated in a well-defined structure, there is a larger flexibility in Hg^II^ coordination, as also indicated by the remarkably different CD features of the As^III^- and Hg^II^-bound species.

### Characterization of the Hg^II^ Coordination Geometry by ^199m^Hg PAC Spectroscopy

^199m^Hg PAC experiments were conducted in order to directly characterize the metal site structure of the Hg^II^-L complex(es).

**Figure 7:**
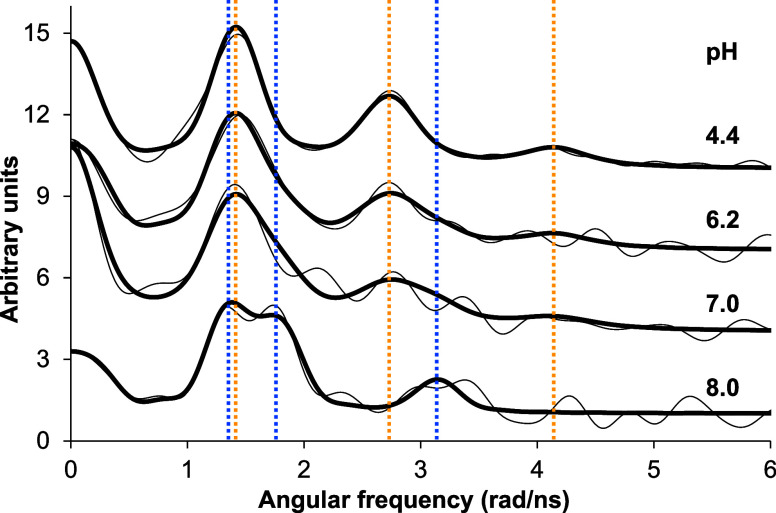
Fourier-transformed ^199m^Hg PAC data (thin lines) and fits (bold-faced lines) obtained for the *c*(Hg^II^): *c*(**L**) = 1:1 system at different pH values (at 1 °C, *c*(**L**) = 34 μM). The sets of yellow and blue vertical dotted lines indicate the positions of signals corresponding to NQI1 and NQI2, respectively.

The samples contained Hg^II^ and the ligand in a 1:1 concentration ratio at different pH values. The PAC data are presented in Table S8 and Figure 7. The data are consistent with essentially only one metal site structure at the starting and end points of a pH titration, i.e., at pH 4.4 and 8.0, while two species are reflected by the data at pH 6.2 and ∼7.0. The p*K*_a_ is estimated to be in the range from 6.0 to 7.0, in accordance with the UV and CD data, *vide supra*. The NQI recorded at low pH (NQI1) agrees excellently with the {HgS_2_} coordination mode.^55,73,75,76^ The high pH form (NQI2) bears some resemblance to previous ^199m^Hg PAC data for trigonal planar structures, for which the quadrupole frequency, ν_Q_, falls in the range of 1.16–1.18 GHz and the asymmetry parameter, η, falls in the range of 0.22–0.31,^55^ although the frequency is lower (1.02 GHz) and the NQI is considerably more asymmetric (η = 0.68). A perfect trigonal pyramidal HgS_3_ structure does not agree with the high asymmetry parameter either: in the ideal case, that would also (like the trigonal planar structure) give η = 0. According to the simple AOM (angular overlap model),^77^ a T-shaped {HgS_3_} structure with equal Hg–S bond lengths gives the same NQI frequency, ν_Q_, as trigonal planar species, however, with an asymmetry parameter η = 1.0. Under subequimolar Hg^II^:protein concentration ratios, the Hg^II^-bound CueR metalloregulator also displayed a pair of parameters (ν_Q_ = 1.04 or 1.13 GHz, η = 0.77 or 0.56 depending on the absence/presence of DNA)^75^ that indicated an intermediate state between a perfect trigonal planar and T-shaped structures. Consequently, in the present system, the most likely structural interpretation of the high pH signal (NQI2) is a distorted {HgS_3_} binding mode. The notion that the structure is distorted agrees well with the coordination by two adjacent cysteinates in the amino acid sequence, imposing geometrical constraints on the positions of the two sulfur atoms in the first coordination sphere. This is substantiated by the optimized structures (Figure S18G), where the S–Hg–S angle for ^2^Cys and ^3^Cys is larger than the other S–Hg–S angles.

### Characterization of the As^III^ and Hg^II^ Coordination Centers by EXAFS Spectroscopy

**Figure 8:**
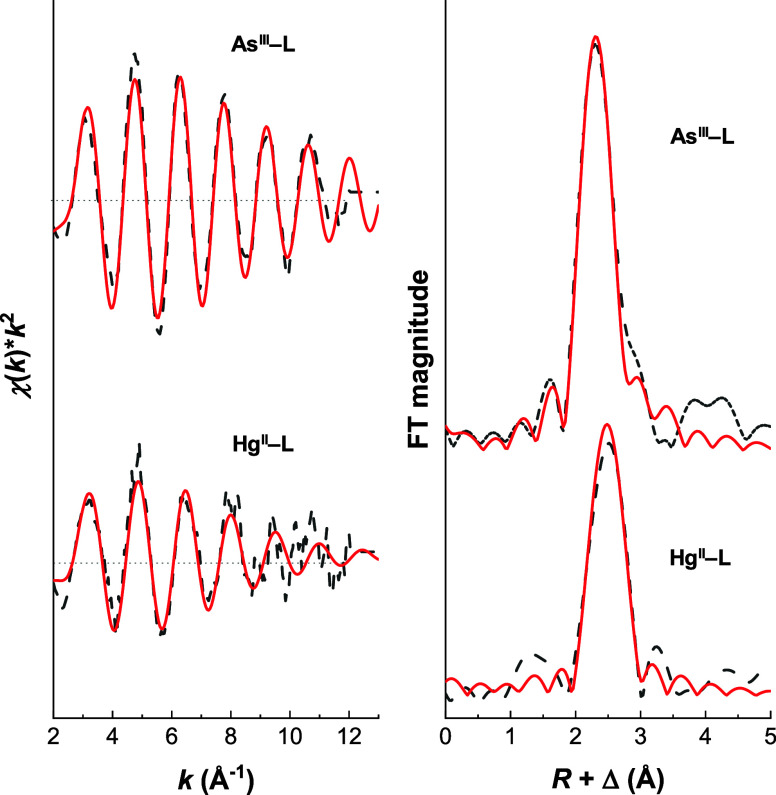
EXAFS spectra and corresponding Fourier transforms of the As^III^-**L** (top) and Hg^II^-**L** (bottom) complexes with the simulated models of a single scattering path (As–S_3_ or Hg–S_3_) shown in red as solid lines. Experimental conditions: *T* = 15 K; 25% (V/V) glycerol; *c*(As^III^): *c*(**L**) = 1:1.2, *c*(As^III^) = 2.8 mM, pH 7.5; *c*(Hg^II^): *c*(**L**) = 1:1.2, *c*(Hg^II^) = 2.9 mM, pH 8.0.

EXAFS spectra were recorded for the As^III^-**L** (As K-edge) complex and the Hg^II^-**L** (Hg L_III_-edge) complex, as well as for two reference samples, using glutathione (GSH) as the ligand; see Figures 8 and S15.

The spectra recorded for the As^III^-**L** complex and the As^III^-GSH reference are highly similar. Fitting the data using a model with only sulfur in the first coordination sphere gives a coordination number of 2.9(1) for both data sets and As–S bond lengths of 2.262(6) Å and 2.257(7) Å for the As^III^-**L** complex and for the As^III^-GSH reference, respectively; see Table S9. The structures optimized using DFT methods, *vide infra*, indicate that there may be minor differences in the three As–S bond lengths, presumably caused by the constraints imposed by the peptide backbone, and this may be reflected in the line width of the Fourier-transformed EXAFS data for the As^III^–peptide complex, which is slightly larger than that for the reference.

Fitting the data recorded for As^III^-**L** with two independent As–S bond lengths gives one As–S bond length of 2.24(5) Å and two of 2.27(4) Å. Most importantly, the bond lengths and coordination number are consistent with the EXAFS data and fits reported by Rosen and co-workers for the mother protein AfArsR (As–S bond length of 2.24 Å)^23^ as well as for another As^III^ sensor protein CgArsR (As–S bond length of 2.25 Å),^24^ indicating that the peptide adequately models the As^III^ binding site of the protein. There is a weak signal in the As^III^-**L** spectrum around 4 Å, which is not accounted for by the fit. This might originate from multiple scattering or atoms at a longer distance from As^III^ than the three sulfurs. This additional signal is not present for the As^III^-GSH reference, possibly because the reference is less ordered. This additional weak signal at large values of *R* cannot unambiguously be discerned for the Hg^II^-**L** complex because the signal-to-noise ratio is inadequate. This might indicate that the Hg^II^-**L** complex is more dynamic and disordered than As^III^-**L**, in agreement with the MD simulations, *vide infra*, and CD spectroscopy.

Fitting the EXAFS data recorded for the Hg^II^-**L** complex with only sulfur in the first coordination sphere gives a coordination number of 2.5(3) and a Hg–S bond length of 2.46(1) Å; see Figure 8 and Table S9. The bond length falls in the range expected for HgS_3_ coordination as determined by X-ray diffraction (2.40–2.51 Å, with an average of 2.44 Å),^78^ as well as by EXAFS data on Hg^II^-GSH complexes.^79,80^ The too-low coordination number (<3) may arise due to significant distortion from the trigonal planar geometry of the Hg^II^ binding site, possibly accompanied by dynamics, as indicated by the relatively large Debye–Waller factor, and in agreement with the molecular dynamics simulations, *vide infra*. Alternatively, the coordination number of 2.5(3) reflects a distribution of HgS_2_ and HgS_3_ species, and fitting a linear combination of two such sites to the EXAFS data leads to 51(9)% HgS_3_ and 49(7)% HgS_2_, with bond lengths of 2.50 and 2.36 Å, respectively (Table S9). Allowing the three-coordinated site to have two different Hg–S bond lengths leads to 69(9)% HgS_3_ and 31(9)% HgS_2_. In either case, the HgS_3_-to-HgS_2_ ratio is higher than expected given the UV, CD, and PAC spectroscopic analyses in this work, indicating that the EXAFS conditions (25% V/V glycerol and low temperature) induce this shift in the equilibrium between HgS_3_ and HgS_2_.

In summary, the EXAFS data for As^III^-**L** indicate the presence of an AsS_3_ complex, in agreement with the interpretation of EXAFS data recorded for the AfArsR^23^ and CgArsR^24^ proteins, while the Hg^II^-**L** complex displays more structural diversity, either as a highly distorted trigonal planar site or due to the coexistence of HgS_2_ and HgS_3_ species.

### DFT-Optimized Structure of the As^III^ and Hg^II^ Binding Segment of the Coordinated Ligand

**Figure 9:**
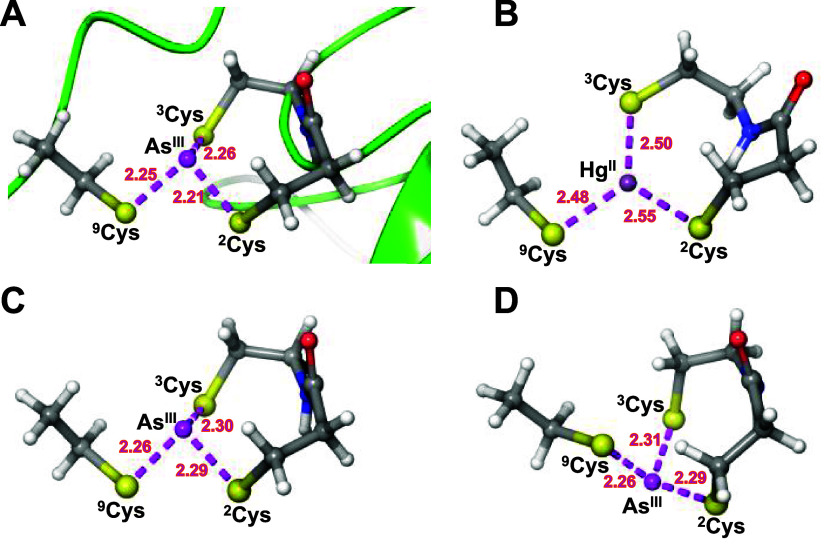
Reference crystal structure (A) and DFT-optimized structures of the ^2^Cys-^3^Cys fragment of **L**, complemented with a bound CH_3_–CH_2_–S^–^ ion (mimicking the distant ^9^Cys unit) in complexes with Hg^II^ (B) and As^III^ in *endo*-conformation (C) and *exo*-conformation (D). Models were optimized by ORCA 5.0.3^81^ (see the Experimental Procedures in the Supporting Information for details). The crystal structure (pdb: 6J05(25)) cutout has been saturated with H atoms. S–metal ion–S angles for the models are shown in Table S10.

The metal site model system excised from the As^III^-bound AfArsR structure, shown in Figures 9A and S17–S19 in a ball and stick representation, was geometry-optimized by DFT calculations. The model with Hg^II^ inserted instead of As^III^ gave the planar Hg^II^ coordination geometry in Figure 9B. In contrast, optimization with As^III^ converged to the structure in Figure 9C, which strongly resembles the crystal structure *endo*-form (Figure 9A).

The structure in Figure 9D is the corresponding *exo*-form, resulting from geometry optimization of the model with As^III^ initially placed on the other side of the plane spanned by the three S atoms. The calculated difference in Gibbs free energy between the optimized *exo* and *endo* models is 2.6 kcal/mol, corresponding, respectively, to ∼1.3 and ∼98.7% population of the two structures at 25 °C. This suggests that a low-lying conformer may coexist with the major *endo* species, providing a possible explanation for the appearance of the additional set of ^1^H NMR resonances besides that of the main complex conformer. Moreover, the DFT optimizations show a metal-ion-specific orientation of the amide plane connecting ^2^Cys and ^3^Cys. The amide plane is parallel to the (near) C3 symmetry axis of the AsS_3_ structure in both the *endo*- and *exo*-models but is tilted in the Hg^II^ model, possibly caused by charge–dipole interactions of the negative charge at the HgS_3_ center and the amide dipole. Consequently, in As^III^ models, the distance between the amide hydrogen and the metal ion is 3.46 Å (*endo*) and 3.39 Å (*exo*), while it is significantly shorter, 2.86 Å, in the Hg^II^ model, reflecting a fundamental difference of the peptide structure in the close surroundings of the As^III^ and Hg^II^ centers.

There is only one other ArsR protein for which the structure of the As^III^-bound form has been characterized by X-ray diffraction, CgArsR.^25^ It is interesting how well the crystal structure of the CgArsR protein, offering two of the As^III^ binding Cys thiols from one monomer and a third Cys thiol from the end of the α2 helix of the other monomer, overlaps with the structure of AfArsR-As, which displays the three As^III^-bound Cys residues in a short sequence of the same protein chain.^25^ In the CgArsR structure, helix α2, connected directly to As^III^, can be regarded as a link from the As^III^ binding site toward the DNA-binding site.

A more comprehensive account of the signaling pathway, operating in ArsR family member proteins, may be found in recent literature.^82−85^ This provides a pathway for signal transmission that is a function of how As^III^ binding may affect the DNA-binding site. Indeed, a plausible link between the metal site and helix α2 is also present in AfArsR-As via a hydrogen bond connecting the amide NH between ^96^Cys and ^97^His and the last carbonyl oxygen of helix α2 (2.7 Å). Hg^II^ binding to the metal site and the changes induced in the surrounding protein fold may disrupt this connection to the DNA-binding domain. One may speculate that the total charge at the As^III^ binding site (=0) and the Hg^II^-binding site (=–1) also affects metal ion selection. The α2 helix in AfArsR (of the other monomer) points the negative end of the helix dipole toward the metal center in the crystal structure,^25^ which may affect the structure of the C-terminal peptide fragment in the Hg^II^-loaded form.

One of the advocated structural origins of the metal ion-selective functioning of metalloregulatory proteins is related to the coordination geometry of the bound metal ion,^86^ which is well-corroborated by our data. It seems likely that the selectivity based on proper folding of part of the protein, induced only by the cognate metal(loid), is operational in other sensor proteins, as well. Indeed, careful comparison of crystal structures of the metal-free and metal-bound forms of various metalloregulators (relying partly on modeled structures using AlphaFold^87,88^ (Uniprot id: B7J952 (AfArsR), P0ACS5 (ZntR)) and I-TASSER^89−91^ (I-TASSER server)) indicates a certain level of flexibility in the metal-free or repressor forms near the effector binding site (Figure S20). Similarly, structural data on the DtxR/MntR family member proteins MntR^92,93^ and ScaR,^94^ the MerR family sensors CueR,^95,96^ ZntR,^95^ and MeR,^97^ the tetrameric NikR,^98^ as well as the ArsR/SmtB family sensory proteins CzrA,^99^ CadC,^100,101^ and AfArsR^25^ all show that the coordinating ligands are only partially arranged in the absence of metal ions. However, metal ion binding reorganizes the coordinating donor groups as well as the surrounding protein skeleton in a way that allows the metal ion to adopt its favored coordination geometry. These sometimes subtle structural changes in the protein scaffold around the metal center may serve as the initiation of the signal transmission pathway toward the DNA-binding domain.

### MD Simulations of Ac-NCCHGTRDCA-NH_2_

**Figure 10:**
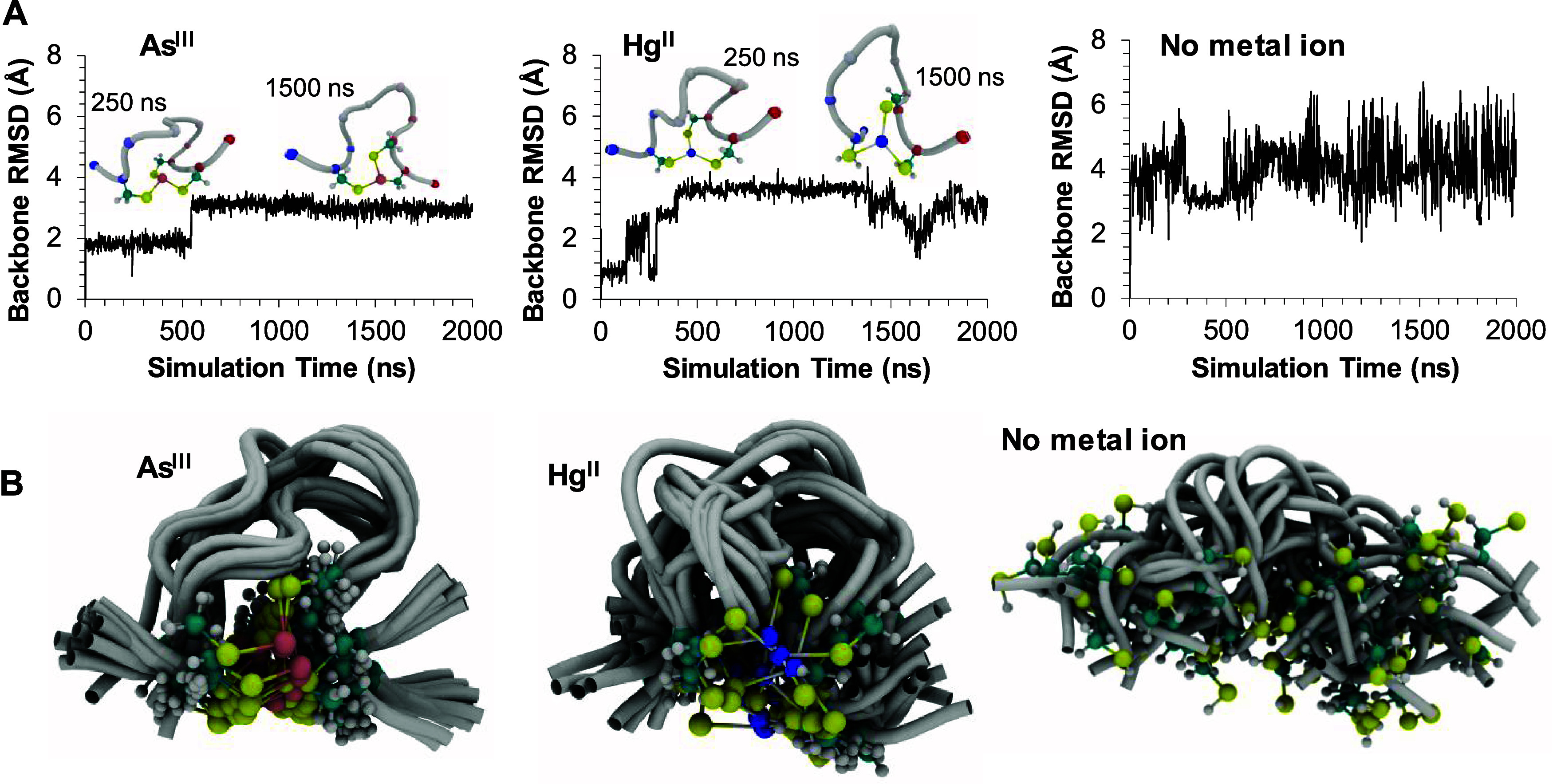
(A) Backbone root-mean-square distance (RMSD) from the initial structure during MD simulations of the Ac-NCCHGTRDCA-NH_2_ peptide with As^III^ (left), with Hg^II^ (middle), and without metal ions (right). Insets show structural snapshots for the As^III^ and Hg^II^ systems at 250 and 1500 ns. C^α^ atoms are indicated by spheres colored by the sequence position (red, N-terminal; blue, C-terminal). (B) Superposition of MD snapshots within each trajectory to highlight conformational variation.

The Ac-NCCHGTRDCA-NH_2_ peptide with coordinated As^III^ populated two main conformational ensembles in the 2000 ns MD trajectory, as indicated by the backbone RMSD plot (Figure 10A, left). Inset structural snapshots at 250 and 1500 ns highlight the characteristics of these ensembles. The backbone atoms of the snapshots have been aligned to allow their comparison. As expected, the first coordination sphere geometry remains largely unchanged upon comparison of the two snapshots. However, the backbone is more expanded at 1500 ns, mainly as a consequence of the disruption of the backbone hydrogen bond between ^2^Cys and ^7^Arg. Indeed, such a hydrogen bond in the As^III^-bound ligand is also suggested by ^1^H NMR spectra recorded at varying temperatures that reflect a −0.2 ppb/K temperature coefficient for the ^7^Arg-NH signal (see Figure S14 with text).^102,103^

In the Hg^II^ system, the first coordination sphere also remains well-conserved. However, the RMSD curve in Figure 10A (middle) reveals increased backbone dynamics relative to those of the system with As^III^. Snapshots at 250 and 1500 ns are displayed for consistency with the As^III^ system, although the RMSD fluctuations indicate more than two conformational ensembles. The metal-ion-free system displays pronounced backbone RMSD fluctuations (Figure 10A, right) due to the vastly increased conformational freedom in the absence of metal ion coordination. The RMSD curves in Figure 10A are further detailed with structural snapshots. The initial and final structures for each simulation are shown in Figure S21. Figure 10B depicts the superposition of frames for each MD trajectory, reduced to 20 frames. This superposition highlights the increase in conformational dynamics, advancing across the subfigures from the left (As^III^) through the middle (Hg^II^) to the right (no metal ion). Thus, the MD simulations demonstrate that the apparent minor local difference in basic inorganic chemical coordination preference for As^III^ (trigonal pyramidal) to Hg^II^ (trigonal planar) has a profound effect on the structure and dynamics of the entire peptide, with As^III^ binding leading to formation of a well-defined structure and presumably the functional form of the AfArsR protein.

## Conclusions

Studies on the metalloid binding fragment of the transcriptional regulator AfArsR revealed that the possible origins of the effector recognition selectivity of the protein are manifested already at the peptide model level. The As^III^ and Hg^II^ binding characteristics of the peptide were compared applying several equilibrium, solution structural, and computational methods at different As^III^/Hg^II^–ligand concentration ratios and pHs. Hg^II^ was chosen as a highly thiophilic metal ion, which may also accommodate a tristhiolate-coordinated environment. The Hg^II^ binding affinity of the AfArsR model peptide was demonstrated to surpass the affinity for As^III^ by an enormous margin, which highlights the significance of the metalloid-selective operation of the ArsR proteins. Titrations of the peptide by As^III^ and Hg^II^, followed by UV and CD techniques, show that tristhiolate-coordinated structures dominate for both ions at pH 7.5; however, the peptide is driven into a fundamentally different conformation in the As^III^-bound species as compared to either the free ligand or the Hg^II^ complex. ^1^H NMR data suggest that the structure of the peptide is significantly more defined in the As^III^ complex than in the presence of Hg^II^. In agreement with this, conformational disorder in MD simulations was lower for As^III^-peptide models relative to Hg^II^- and metal ion-free models. The DFT-optimized structure of the As^III^-bound ligand, using a small model, reflects that the structure of the AfArsR-As, displaying an endo-conformation, is very closely retained, but the calculations also indicate the possibility for the existence of the *exo*-type species, in line with the NMR data. This minor isomer represents only a very small fraction of the ligand for the peptide model system, and it is presumably not relevant with regard to the native metalloid site of the protein. Optimization of the minimal model counterpart of the small endo model, i.e., As(CH_3_S)_3_ (Figures S18 and S19), leads to a closely overlapping structure to that of the small model. This implies that the amino acid sequence with the adjacent ^2^Cys and ^3^Cys residues allows for the organization of part of the metalloid binding site where the optimal trigonal pyramidal structure is not significantly distorted by the constraints imposed by the protein backbone. In contrast, DFT calculations showed that Hg^II^ can accommodate a distorted trigonal planar geometry in the energetically favored structure of its peptide complex, in good correlation with ^199m^Hg PAC results, which ultimately means that the metal center is significantly displaced from the position where the metalloid is located in the crystal structure. It is remarkable that the amide group of the Cys–Cys fragment, being perpendicular to the AsS_3_ plane, is tilted in the Hg^II^-bound peptide possibly because of the electrostatic interaction between the HgS_3_ center and the amide dipole.

In summary, our spectroscopic and computational results indicate that the local peptide fold, shaping an optimal trigonal pyramidal coordination geometry via the binding of the three Cys thiolate groups, plays an important role in the selection of the cognate effectors. The fundamentally different binding preferences of As^III^ and Hg^II^ to the effector binding site of AfArsR might lead to a disparate protein structure in the As^III^- vs the Hg^II^-bound forms, at least in the surroundings of the metal center, with a potential impact on the protein function by controlling metal ion recognition. In addition, all of the data in this work imply that for target ions such as As^III^, where it is difficult to establish selectivity over other biologically relevant metal ions by design of a high-affinity binding site, an alternative route to selectivity is found, where only the target ion triggers a conformational change—for AfArsR, it is a change from disorder of the free peptide to order of the As^III^–peptide complex—which leads to the active form of the protein. In a broader perspective, the metal ion-induced organization of the structurally flexible metal binding site, observed in the peptide model of AfArsR, seems to operate also in a number of metal sensor proteins, and this may be a key step in metal ion selection. The flexibility of the metal binding site allows the cognate metal ion to reorganize the local protein fold to achieve the preferred coordination geometry, leading to signal transmission from the metal site to the DNA-binding domain, a pathway that is disrupted by the different coordination structure of the noncognate ions.

## Supporting Information Available

Detailed description of the applied materials, sample preparation protocols, and experimental methods; additional information and figures of As^III^ and Hg^II^ titrations of the ligand followed by UV and CD spectroscopies; determination of the As^III^-binding affinities; evaluation of pH-potentiometric data and determination of Hg^II^-binding affinities; characterization of As^III^ and Hg^II^ binding by ESI-MS; additional NMR data and figures; table of fitted ^199m^Hg PAC parameters; table of fitted EXAFS parameters; figure of the spectra recorded for reference samples; figure of simulated species distribution for Hg^II^-GSH; additional data and figures of DFT and MD studies; and appendix describing semiempirical (SESCA and PDBMD2CD) approaches to predict CD spectra, as well as TD-DFT calculations of the excited states of the As^III^-bound peptide complex and the related electronic CD (PDF)
