# Supplementary material for: AsIII Selectively Induces a Disorder-to-Order Transition in the Metalloid Binding Region of the AfArsR Protein
Source: J Am Chem Soc. 2024 May 31;146(25):17009–22. doi: 10.1021/jacs.3c11665 (PMC11212059; doi:10.1021/jacs.3c11665)
Supplement: Supplementary file 1 — ja3c11665_si_001.pdf [file ja3c11665_si_001.pdf]

Supporting Information

As^{III} selectively induces a disorder to order transition in the metalloid binding region of the AfArsR protein

Annamária Tóth,[#] Kadosa Sajdik,[#] Béla Gyurcsik,[#] Zeyad H. Nafaee,[#] Edit Wéber,[ʃ,ɘ] Zoltan Kele,[ʃ] Niels Johan Christensen,[&] Juliana Schell,[§,¦] Joao Guilherme Correia,[¿,¦] Kajsa G.V. Sigfridsson Clauss,[⊥] Rebecca K. Pittkowski,[‡] Peter Waaben Thulstrup,[‡] Lars Hemmingsen[‡] and Attila Jancsó[#] *

[#] Department of Molecular and Analytical Chemistry, University of Szeged, Dóm tér 7-8. H-6720 Szeged, Hungary; [ʃ] Department of Medical Chemistry, University of Szeged, Dóm tér 8. H-6720 Szeged, Hungary; [ɘ] HUN-REN-SZTE Biomimetic Systems Research Group, Dóm tér 8. H-6720 Szeged, Hungary; [&] Department of Chemistry, Faculty of Science, University of Copenhagen, Thorvaldsensvej 40, 1871 Frederiksberg C, Denmark; [§] Institute for Materials Science and Center for Nanointegration Duisburg-Essen (CENIDE), University of Duisburg-Essen, 45141 Essen, Germany; [¿] Centro de Ciẹncias e Tecnologias Nucleares, Departamento de Engenharia e Ciẹncias Nucleares, Instituto Superior Técnico, Universidade de Lisboa, 2695-066 Bobadela LRS, Portugal; [¦] European Organization for Nuclear Research (CERN), CH-1211 Geneva, Switzerland; [⊥] MAX IV Laboratory, Lund University, P.O.Box 118, SE-221 00 Lund, Sweden; [‡] Department of Chemistry, University of Copenhagen, Universitetsparken 5, 2100 København Ø, Denmark

Table of Contents

Experimental Procedures 3

Materials 3

UV absorption spectroscopy 3

Determination of the apparent stabilities of the complexes of As^{III} and Hg^{II}........ 4

Circular dichroism (CD) spectroscopic studies 4

NMR spectroscopy 5

Mass spectrometry 5

^{199m}Hg PAC spectroscopic experiments 5

EXAFS spectroscopy 6

pH-potentiometric measurements and determining thermodynamic stability constants for the Hg^{II}-complexes 7

DFT calculations on the tristhiolate-coordinated As^{III} and Hg^{II} centers of the complexes formed with the studied ligand 8

MD simulations 9

Results and Discussion 11

UV absorption spectroscopy: Titration of the ligand by As^{III} and determination of the As^{III} binding affinity 11

UV absorption spectroscopy: Titration of the ligand by Hg^{II} 12

Hg^{II} binding affinity of the ligand determined by the combination of competition experiments and pH-potentiometric titrations 14

Circular dichroism (CD) spectroscopy 18

ESI-MS data characterizing As^{III} and Hg^{II} binding by the ligand 19

^{1}H NMR spectroscopy 21

^{199m}Hg PAC data 19

EXAFS data 30

Computational studies 33

References 43

Appendix 46

Prediction of CD spectra by semiempirical methods 46

Prediction of CD spectral properties using time-dependent density functional theory (TD-DFT) of the As^{III} bound NCCHGTRDCA peptide 48

Experimental Procedures

Materials

The *N*-acetyl-Asn-Cys-Cys-His-Gly-Thr-Arg-Asp-Cys-Ala-NH_2 (**L**) peptide (98.9 % purity) was acquired from CASLO, Denmark. Anhydrous arsenic(III) oxide, mercury(II) perchlorate hydrate, mercury(II) chloride, lead(II) chloride, zinc(II) perchlorate hexahydrate, potassium antimonyl tartrate trihydrate, D_2O, Tris(2-carboxyethyl)phosphine hydrochloride (TCEP) and 3-(trimethylsilyl)propionic-2,2,3,3-d_4 acid sodium salt (TSP-d_4) were purchased from Merck (Sigma-Aldrich). Cadmium(II) perchlorate hexahydrate was a product of Alfa Aesar (Thermo Scientific Chemicals). All chemicals were used without further purification. The stock solutions of the peptide, with a desired concentration in the range of 1.0×10^{-3} – 1.0×10^{-4} M, were prepared by dissolving the solid material in a perchloric acid solution (*c* = 1.0×10^{-4} M). The exact concentration of the peptide was determined by the Ellman's test where DTNB (5,5'-dithhiobis-(2-nitrobenzoic acid)) reacts with the thiol groups of the peptide producing one TNB^{2-} (2-nitro-5-thiobenzoate ion) per each derivatized thiol group with ε_{412} = 14150 M^{-1} cm^{-1} molar absorbance.[1,2] Arsenous acid stock solution (*c* ~ 5.0×10^{-3} M) was prepared from a precisely weighed quantity of arsenic(III) oxide dissolved in sodium hydroxide solution (*c* = 0.1 M). Its pH was adjusted to ca 3–4. The mercury perchlorate stock solution (*c* ~ 0.02 M), containing perchloric acid in a concentration of 0.1 M to prevent the hydrolysis of the metal ion, as well as stock solutions of cadmium perchlorate (*c* ~ 0.02 M) and zinc perchlorate (*c* ~ 0.02 M), were standardized complexometrically. Mercury chloride, lead chloride and antimonyl tartrate solutions of a known concentration were prepared by dissolving precise weights of the high purity solids in MilliQ water. In all samples, except those prepared for ESI-MS studies, the ionic strength was adjusted to 0.1 M by sodium perchlorate (Sigma-Aldrich). The pH of the samples was adjusted to the desired values by sodium hydroxide (*c* = 0.1 M) and perchloric acid (*c* = 0.1 M) solutions. The pH was measured by a Metrohm 6.0234.100 combined pH glass electrode connected to an Orion 710A digital pH-meter calibrated at room temperature (*T* = 298 K) against standard buffer solutions. pH-potentiometric titrations were performed by 0.1 M sodium hydroxide solutions (Aldrich), which were standardized using potassium hydrogen phthalate (Sigma-Aldrich). To prevent the oxidation of As^{III} and/or the thiol groups of the ligand, the sample preparations were accomplished under argon atmosphere.

UV absorption spectroscopy

UV absorption spectra were measured on a Thermo Scientific Evolution 220 spectrophotometer in the wavelength range of 200 – 400 nm in a quartz optical cell with 1.00 cm path length, covered by a Teflon cap. The typical concentration of the model peptide was 50 μM in the samples except those containing Hg^{II} where the concentration of the peptide was 40 μM. Spectra in the As^{III} – **L** system were recorded at pH = 2.0, 4.5, 6.0 and 7.5. Each series included 14 individual samples with the *c*(As^{III}) : *c*(**L**) ratio varying in the 0 – 5 range. In order to adjust the pH, we used perchloric acid for pH = 2 (*c* = 0.01 M), acetate buffer for pH = 4.5 (*c* = 15 mM), MES buffer (2-(N-morpholino)ethanesulfonic acid, VWR) for pH = 6.0 (*c* = 15 mM) and phosphate buffer for pH = 7.5 (*c* =15 mM). Because of the rather slow complexation rate of As^{III} under acidic conditions, 20 min – 26 h equilibration time was applied after the sample preparation, depending on the pH of the samples (pH = 7.5: 20 min; pH = 2.0: 26 h). Two series of UV spectra, both involving 15 individual samples, were measured in the Hg^{II} – **L** system covering the *c*(Hg^{II}) : *c*(**L**) = 0 to 2 concentration ratio range. Moreover, pH-dependent experiments were implemented in the pH range of 2.0 – 11.0 at *c*(Hg^{II}) : *c*(**L**) = 0:1, 0.5:1 and 1:1 ratios by performing the titrations in a thermostated cell (*T* = 298 K). The obtained absorbance data, after background correction, were corrected for dilution and normalized to *c* = 50.0 μM or 40.0 μM peptide concentration. The presented absorbances were obtained by averaging the data of two parallel experiments. The effect of various divalent metal ions, as well as As^{III} and Sb^{III}, on the UV absorption of the peptide were compared by recording spectra in samples containing the metal ions and **L** in a 1:1 (Hg^{II}, Cd^{II}, Zn^{II}, Pb^{II}) or 2:1 concentration ratios with a ligand concentration of 10.0 μM. The excess of As^{III} and Sb^{III} ensured that the peptide was fully transformed into its complexed form. Data were corrected by the weak absorption related to the unbound (excess) of arsenous acid and antimonyl tartrate.

Determination of the apparent stabilities of the complexes of As^{III} and Hg^{II}

The apparent stability constants of the As^{III} complexes at pH = 2.0, 4.5, 6.0 and 7.5 were calculated by evaluating the relevant As^{III} : peptide concentration ratio dependent series of UV spectra in a selected wavelength range (235 – 330 nm) by the computer program PSEQUAD.[3] The input file for each pH value included two parallel series of baseline corrected spectra at increasing $c(As^{III})$: $c(\mathbf{L})$ ratios, the total concentrations of As^{III} and the ligand corresponding to the individual measurement points, as well as the composition matrix built from two basic components, As^{III} and **L**, and the molar absorption spectrum of the ligand calculated from the first points of the series.

Since Hg^{II} is bound extremely tightly by the thiolate groups of cysteine residues, we could only determine the apparent stability of the Hg^{II}-peptide complex by implementing competition studies with iodide ions at pH = 2.0. These measurements were based on the well-defined stepwise complex formation of Hg^{II} with I^- allowing the displacement of the ligand from Hg^{II} by the iodide ions. The stability constants of the Hg^{II}-I^- complexes ($[HgI]^+$, HgI_2, $[HgI_3]^-$, $[HgI_4]^{2-}$) were obtained from the literature[4] and recalculated according to the SIT (Specific Ion-interaction Theory) model[5,6] for the applied conditions (pH = 2.0, I = 0.1 M $NaClO_4$), leading to the values $\log\beta_1$ = 13.05, $\log\beta_2$ = 24.09, $\log\beta_3$ = 27.84 and $\log\beta_4$ = 29.91, as described in details previously.[7] Evaluation of the titrations of Hg^{II} by iodide ions ($c(Hg^{II})$ = 55.0 μM), using data in the wavelength range of 270 – 400 nm, resulted in slightly refined $\log\beta_3$ and $\log\beta_4$ values (27.77 and 29.92, respectively) and molar absorption spectra of the Hg^{II}-I^- complexes, used afterwards as fixed input parameters for the Hg^{II} – **L** – I^- ternary system.[7] Next, a sample of Hg^{II} and the peptide ($c_L = 1.1\times c(Hg^{II})$ = 55.0 μM) was titrated by iodide ions up to a ca. 2800-fold excess of I^-. The slight excess of the ligand was applied to prevent the formation of Hg^{II}-I^- complexes preceding the displacement process that could occur if a tiny fraction of Hg^{II} is not bound to the peptide. The pH of the sample was re-adjusted to pH = 2.0 by adding small volumes (2-3 μL) of a c = 0.1 M $HClO_4$ solution whenever it was necessary. The spectra of the Hg^{II} solution, the free peptide, the solutions of KI, as well as the sample of Hg^{II} and the ligand in the absence of iodide ions were recorded to obtain the molar absorption spectra of these species. The obtained data were used in the evaluation of the ternary system refining the apparent stability constant of the [Hg**L**] complex at pH = 2.0 according to the following formulae:

$$\mathrm{Hg} + \mathbf{L} \rightleftharpoons \mathrm{Hg}\mathbf{L} \qquad K_{\mathrm{Hg}\mathbf{L}}^{\mathrm{pH2.0}} = \frac{[\mathrm{Hg}\mathbf{L}]}{[\mathrm{Hg}]_{\mathrm{free}} \times [\mathbf{L}]_{\mathrm{free}}}$$

where Hg represents the free form of Hg^{II} ions (not bound to the peptide or I^-) while **L** represents the unbound ligand in general, independently of its protonation state, and similarly, Hg**L** stands for the Hg^{II}-peptide monomeric complexes independently of the form of the coordinated ligand.

Circular dichroism (CD) spectroscopic studies

CD spectra were recorded on a JASCO J-1500 CD spectrometer with 1 nm steps at 50 nm/min scan speed, 2 s D.I.T. and 200 mdeg/1.0 dOD CD scale accumulating 4 scans for each sample measurements. The samples were prepared similarly to those used in the UV absorption studies, with a few differences in the conditions. The (semi)metal/ligand concentration ratio dependent experiments were carried out at 298 K at pH = 2.0, 7.5 and 8.7 with both Hg^{II} and As^{III} using perchloric acid for pH = 2.0 and phosphate buffer for pH = 7.0, while boric acid was applied to adjust pH = 8.7. The $c(Hg^{II})$: $c(\mathbf{L})$ concentration ratios were matched to those applied in the UV measurements, but with As^{III} 11 individual samples were measured only between $c(As^{III})$: $c(\mathbf{L})$ = 0 to 3, based on the results of the UV spectroscopic experiments. The spectra were recorded in the wavelength range of 185 – 360 nm using a cylindrical cuvette with 0.1 mm path length ($c(\mathbf{L})$ = 650 μM and c(buffer) = 20 mM), or in the wavelength range of 220 – 360 nm in a conventional quartz cuvette with 1.0 cm path length ($c(\mathbf{L})$ = 65 μM, c(buffer) = 10 mM). Spectra were also obtained for samples containing Cd^{II} and **L**, Zn^{II} and **L**, Pb^{II} and **L** and Sb^{III} and **L** in a 1:1 concentration ratio using 0.1 mm path length ($c(\mathbf{L})$ = 650 μM, c(buffer) = 20 mM). For samples of Sb^{III}, the very weak contribution of the released tartrate ion to the spectra was taken into correction (CD-spectrum for *L*-tartrate at a condition representing the released tartrate ions in the displacement process was recorded).

NMR spectroscopy

1D and 2D ^{1}H NMR measurements were carried out on a Bruker Avance III 600 MHz instrument equipped with a 5 mm CP-TCI triple resonance cryoprobe. Samples were prepared in a H_2O/D_2O 90/10 *V*/*V* % solvent mixture with a peptide concentration of $c = 1.0\times10^{-3}$ M. Chemical shifts were referenced to the signal of TSP-d_4 (0.000 ppm) used as an internal reference. The ^{1}H NMR spectra of the peptide in the absence of metal ions were recorded at pH = 2.0 and 7.5 serving as references for the various experiments performed in the presence of As^{III} or Hg^{II}. Spectra for samples of As^{III} and the ligand with a concentration ratio of 1:1 were recorded both at pH = 7.5 and 2.0. The Hg^{II}: peptide system was investigated under various conditions. 1D and 2D experiments were run at pH = 2.0, using an equimolar ratio of the components. At pH = 7.5, spectra were acquired both with a 0.5:1 and 1:1 Hg^{II}:ligand concentration ratios. 2D NMR spectra were recorded only for the latter sample. Samples were measured at different temperatures, e.g. at 298 K and at 282 K allowing for a more univocal assignation of resonances. The desired pH values were set by $HClO_4$ at pH = 2.0 (c = 0.01 M in the sample) or by a phosphate buffer at pH = 7.5 (c = 20 mM in the sample). The provided pH values were measured at 298 K. Several samples were also prepared and measured with the presence of the reducing agent TCEP in order to ensure/verify the reduced (thiol) form of the peptide.

For the ^{1}H signal assignment of the ligand, 2D TOCSY and ROESY experiments were acquired. ^{1}H-^{1}H TOCSY measurements were performed with a mixing time of 80 ms applying the DIPSI2 sequence for mixing, and the number of scans was 32. Spectra were acquired with 2048(F2) and 512(F1) complex points in a spectral width of 11 ppm. ^{1}H-^{1}H ROESY experiments were acquired with the same spectral width and complex points using 64 scans and a mixing time of 400 ms. To eliminate the residual H_2O/HDO resonances, the excitation sculpting solvent suppression pulse scheme was applied. For the analysis of the solution phase conformation of the peptide in the presence of As^{III} at pH 7.5, experiments were repeated for a sample containing $c = 3.0\times10^{-3}$ M ligand, and a series of 2D NOESY and ROESY spectra were collected using various mixing times (400 ms, 300 ms and 200 ms). Spectra were processed by the Topspin 3.6.5 software (Bruker) with a cosine-bell window function applying single zero-filling and automatic baseline correction.

Mass spectrometry

Full scan and MS/MS mass spectra of the complexes were acquired in the range of 100 to 1100 *m*/*z* with a Q Exactive Plus quadrupole-orbitrap mass spectrometer (Thermo Fisher Scientific, Waltham, MA, USA) equipped with a heated electrospray (HESI). Analyses were performed in positive ion mode applying flow injection mass spectrometry with a mobile phase of 50% (*v*/*v*) aqueous acetonitrile. The flow rate was 0.3 ml/min. Five µl aliquots of the samples were loaded into the flow. The ESI capillary was adjusted to 3.5 kV and N_2 was used as a nebulizer gas. In a typical sample the concentration of the ligand was ca. 6.5×10^{-5} M and the concentration of As^{III} or Hg^{II} varied according to the applied metal-to-ligand ratio. For experiments under slightly acidic conditions the pH of samples was set by acetic acid to pH = 3.2. Ammonium hydrogen carbonate was used to prepare samples at pH = 7.8 and 9.1, the latter adjusted to the desired value by 25 % ammonia solution. The As^{III}-peptide system was studied at pH = 7.8 applying 0.5:1, 1:1 and 4:1 As^{III}:L concentration ratios. Spectra for the Hg^{II}-L system were recorded at various Hg^{II}:peptide concentration ratios and at different pH values, i.e. $c(Hg^{II}) : c(\mathbf{L})$ = 0.25:1 (pH = 9.1), $c(Hg^{II}) : c(\mathbf{L})$ = 1:1 (pH = 7.8 and pH = 3.2) and $c(Hg^{II}) : c(\mathbf{L})$ = 1.5:1 (pH = 7.8). The theoretical isotope patterns were determined by Xcalibur Software (Thermo Fisher Scientific, Waltham, MA, USA).

^{199m}Hg PAC spectroscopic experiments

Isotope production

^{199m}Hg PAC measurements were performed at the ISOLDE laboratories at CERN.[9] ^{199m}Hg was produced by irradiating a liquid Pb target with protons (1.4 GeV) along with several other radionuclides, and a general purpose separator (GPS) provided a very pure beam of ^{199m}Hg, which was implanted into ca. 180 µL MilliQ ice sitting in a Teflon cup on a copper base, kept at low temperature via a cold finger immersed into liquid nitrogen.

Preparation of samples for ^{199m}Hg PAC spectroscopy

The irradiated frozen liquid, containing ^{199m}Hg isotopes, was thawed and mixed with 10 µL of a non-radioactive $HgCl_2$ solution. To this sample 20 µL of the peptide stock solution, prepared in 0.001 M $HClO_4$, was added. 10 µL of various concentrated (0.4 M) buffer solutions and 5 µL of a 4.0 M $NaClO_4$ solution was mixed into the samples. According to the desired pH values, HEPES (pH ~ 7.9), MES (pH ~ 6.0), acetate (pH ~ 4.4) and a mixture of HEPES and MES (pH ~ 6.8) buffers were utilized. The final pH values were adjusted by 0.1 M NaOH or $HClO_4$ solutions. Finally, sucrose (55 % *w/w*) was added to the mixtures and dissolved by using a Vortex. The pH values were measured at room temperature and recalculated to the temperature of the experiments (1 °C) by the known temperature dependence of the various buffers.[8] The pH values of the samples were verified after the measurements and the decay of radioactive mercury. The concentration ratio of the peptide and Hg^{II} was 1:1 in the prepared samples, $c(Hg^{II}) = c(\mathbf{L}) = 3.4\times10^{-5}$ M.

PAC instruments and data analysis

A digital PAC setup, DIGIPAC,[10] using #6 1.5″×1.5″ $LaBr_3$(Ce) detectors was used for the spectroscopic measurements. The time resolution was 0.7 ns, and the time-per-channel was 0.04883 ns. Data analysis was carried out with the Winfit program (provided by prof. T. Butz) using 500 data points (excluding the first 10 points), except for the reference compound[11] for which 300 data points were used. A Lorentzian line shape was used with the parameter δ accounting for line broadening due to a static distribution of electric field gradients (EFGs). Fourier transformation of the data and fits was carried out using 600 points after mirroring 300 data points using a Keiser-Bessel window with the window parameter equal to 4. Initially the PAC parameters, see Ref. 12, were fitted to the data recorded at pH 4.4 and 8.0, respectively, giving nuclear quadrupole interaction 1 (NQI1 at pH 4.4) and NQI2 (at pH 8.0). Next, the spectra at intermediate pH values (6.2 and ~7.0) were analysed with these NQIs, with only the amplitudes, the base line, and inverse rotational correlation time as free parameters, see Table S8. As a test, also the spectra recorded at pH 4.4 and 8.0 were analysed with the two fixed NQIs, but this resulted in no amplitude (less than 0.02) for the NQI2 and NQI1, respectively, i.e. there is only one measurable NQI in each of these two data sets.

In ^{199m}Hg PAC spectroscopy the NQI is measured in the intermediate nuclear level (E = 158.3 keV excited state, I = 5/2) of a γ-γ cascade. For nuclear spin 5/2, a time independent EFG, and randomly oriented molecules, the NQI is described by[12,13]:

$$A_{22}^{eff}\,G_{22}(t) = A_{22}^{eff}\,(a_0 + a_1\cos(\omega_1 t) + a_2\cos(\omega_2 t) + a_3\cos(\omega_3 t))$$

where A_{22}^{eff} is the effective anisotropy, and a_i and ω_i depend on the quadrupole coupling constant ν_Q and the asymmetry parameter η of the NQI.[13] The experimental equivalent of $A_{22}^{eff}G_{22}(t)$ to which the NQI parameters are fitted is[13]:

$$R(t) = 2\,\frac{W(180°,t) - W(90°,t)}{W(180°,t) + 2W(90°,t)}$$

where $W(180°,t)$ and $W(90°,t)$ are the geometrical mean of coincidence spectra recorded with 180° and 90° between detectors.

EXAFS spectroscopy

Instrumentation and data collection

As K-edge (11867 eV) and Hg L_{III}-edge (12284 eV) x-ray absorption spectra were collected in transmission or fluorescence mode at the Balder beamline of the MAX IV Laboratory synchrotron light source in Lund, Sweden. The LN2 cooled double crystal monochromator equipped with Si111 crystals was calibrated to the first inflection point of a Se-foil (12658 eV). The X-ray beam at the sample position was defocused to a tilted rectangle H 200 x V 400 µm^2. Gas filled ionization chambers one before (I0: 50 mbar Ar, 950 mbar N_2, voltage 1.7 kV) and one after the sample (I1: 350 mbar Ar, 650 mbar N_2, voltage 2.5 kV) were used as detectors for incoming x-ray intensity and transmission. To detect fluorescence, the 7 element Ge detector (Mirion Technologies Inc., USA) was used set to the emission line of the element of interest (As K alfa/ Hg L alfa), and a Ge filter was used to decrease elastic scattering counts on the detector. EXAFS scan settings were: 30 sec/scan, edge range −100 to

100 relative to the edge energy with energy-resolution 0.2 eV, post edge range up to k 16/13 with k-resolution 0.025 Å^{-1}. The frozen sample solutions were cooled down to 15 K in a closed cycle He cryostat (in house built) during measurements. The sample was moved vertically by 0.3 mm after two scan repeats (á 30 sec) per beam spot to avoid radiation damage.

Preparation of samples for EXAFS spectroscopy
Samples of As^{III}–**L** and Hg^{II}–**L** were prepared under Ar atmosphere by mixing stock solutions of the peptide, arsenous acid or mercury perchlorate and a phosphate buffer (containing sodium perchlorate for adjusting the ionic strength) and their pH were adjusted to the desired values (As^{III}–**L**: pH = 7.5; Hg^{II}–**L**: pH = 8.0). The somewhat higher pH of the Hg^{II}–**L** sample was set according to the pH-speciation profile (see Figure S5 below) to promote the formation of species with a HgS_3 coordination environment. The stock solutions were all prepared in Ar-degassed MilliQ water and their concentration were set/determined as described in the Materials section.

The As^{III}–GSH reference sample was prepared by dissolving a precisely weighed mass of glutathione in Ar-degassed MilliQ water followed by the addition of a calculated volume of arsenous acid to reach a 1:15 $c(As^{III})$:c(GSH) concentration ratio. The pH of the sample was adjusted by NaOH solutions of 2.0 M and 0.1 M to pH = 7.6. The Hg^{II}–GSH reference was prepared in a rather similar way where we followed the protocol described by Mah et al..[14] The final sample contained a 12-fold GSH excess over Hg^{II} and the pH was adjusted by NaOH (2.0 M and 0.1 M) to pH = 8.2. The two reference solutions did not contain buffer or additional salt.

Before the experiments, glycerol (25 % V/V) was added to all the four samples (the pH effect was found to be negligible). Approximately 50 μL of each solution was transferred into cylindrical sample cells (6 mm diameter) made in acrylic glass (thickness 1.5 mm) covered on both sides by Kapton tape windows secured with peak clamps. The sample holder with the loaded samples was flash frozen in liquid nitrogen and transferred frozen into the cryostat (15 K).

The final concentrations (after the addition of glycerol) and pH values for the four samples were as follows: $c(As^{III})$: c(**L**) = 2.8 mM : 3.3 mM, pH 7.5; $c(As^{III})$: c(GSH) = 10.2 mM : 154 mM, pH 7.6; $c(Hg^{II})$: c(**L**) = 2.9 mM : 3.5 mM, pH 8.0; $c(Hg^{II})$: c(GSH) = 21.0 mM : 252 mM, pH 8.2.

Data analysis
The x-ray absorption spectra collected in fluorescence mode were used for the fitting of the As^{III}–**L** and Hg^{II}–**L** samples, while for the GSH reference samples the data collected in transmission mode were used. Data reduction and analysis were performed with the DEMETER software package based on the IFEFFIT libraries.[15] Before data treatment, each scan was examined to exclude spectral anomalies. Data processing included data averaging, background correction and normalization by the edge jump. The energy units (eV) were converted to photoelectron wave number k units (Å^{-1}) by assigning the photoelectron energy origin, E_0, corresponding to k = 0, to the first inflection point of the absorption edge. For the fitting of the extended x-ray absorption fine structure (EXAFS), the $\chi(k)$ functions were weighted with k^2. The k-range used for the Fourier transform was k = 3 – 12 for Hg and k = 14 for As. The local structure parameters were refined from EXAFS functions by non-linear least square (NLLS) fitting in an R-space range of 1.3–3.3 Å.

pH-potentiometric measurements and determining thermodynamic stability constants for the Hg^{II}-complexes

Acid dissociation constants of the free peptide and formation constants of the Hg^{II}-peptide complexes were determined by pH-potentiometric titrations performed with samples of the ligand in the absence and presence of Hg^{II} ions in the pH range of 2.5 – 11.4. Titrations were executed in a thermostated cell (T = 298.0 ± 0.1 K) in aqueous solutions (I = 0.1 M $NaClO_4$) under argon atmosphere applying a continuous stirring. The standard base titrant (0.1 M NaOH) was dosed into the samples by a PC-controlled autoburette (Metrohm 765 Dosimat). Calibration of the system was performed according to our protocol reported earlier.[16,17] The applied concentration of the peptide was $c_L = 1.2\times10^{-3}$ M and the Hg^{II} concentration was varied to set 0:1, 0.5:1, 1:1

and 1.4:1 Hg^{II}:peptide concentration ratios. Protonation and complex formation processes were characterized according to the following general equilibrium reaction:

$$\mathrm{pM + qH + rL \rightleftharpoons M_pH_qL_r}$$

Data were fitted by the Hyperquad program[18] providing overall formation constants:

$$\beta_{\mathrm{M_pH_qL_r}} = \frac{[\mathrm{M_pH_qL_r}]}{[\mathrm{M}]^{\mathrm{p}}[\mathrm{H}]^{\mathrm{q}}[\mathrm{L}]^{\mathrm{r}}}$$

where M indicates the Hg^{II} ions, H the protons and here L stands for the fully deprotonated form of the ligand (and not the ligand in general). Charges are omitted in the above formulae but can be easily calculated based on the composition and the charge of the fully protonated peptide, $[H_5L]^{2+}$. It is worth noting that according to the highly basic character of the Arg residue the guanidium sidechain was considered to be protonated and thus positively charged throughout the titrations and thus having no effect on the titration curves in the studied pH-region.

Because of the very high stability of the Hg^{II}-complexes evaluation of pH-potentiometric data cannot provide thermodynamic overall formation constants for the species, however, acid dissociation constants (pK_a) characterizing the transformations between the differently protonated complexes may be accurately determined.[19] Furthermore, if the formation constant of the most plausible monomeric species, dominating at the start of the titrations (between pH = 2–3) can be estimated, the fixing of this logβ value in the data fitting process may allow to derive the formation constants for all other complexes.[19] As we have demonstrated in a previous study, the apparent stability constant determined for the binding of Hg^{II} to the peptide at pH = 2.0 may serve as the basis for estimating the formation constant of the monomeric Hg^{II}-complex dominating at low pH.[7] The composition of this latter species may be given as $[HgH_3L]^{2+}$, assuming two Hg^{II}-coordinated thiolate groups, and is formed via the following process:

$$\mathrm{Hg^{2+} + [H_5L]^{2+} \rightleftharpoons [HgH_3L]^{2+} + 2H^+}$$

Based on the pK_a values of the peptide (see later) the unbound ligand is present only as $[H_5L]^{2+}$ at pH = 2.0 and the overall concentration of the complexes can be approximated by the equilibrium concentration of $[HgH_3L]^{2+}$. Consequently, the equilibrium constant characterizing the above complexation reaction maybe, indeed, approached by the apparent stability constant determined for pH = 2.0 ($K^{pH2.0}$), vide supra. (Note that charges are omitted for simplicity in all following expressions.)

$$K_{\mathrm{Hg}\mathbf{L}}^{\mathrm{pH2.0}} = \frac{[\mathrm{HgH_3L}]}{[\mathrm{Hg}] \times [\mathrm{H_5L}]}$$

Substituting the concentration $[HgH_3L]$ by the term $\beta_{HgH3L}\times[Hg]\times[H]^3\times[L]$ and the concentration $[H_5L]$ by the term $\beta_{H5L}\times[H]^5\times[L]$ in the equilibrium constant equation, the formation constant, β_{HgH3L} can be calculated (using 0.01 M for the H^+ concentration, i.e. pH = 2.0):

$$\beta_{\mathrm{HgH_3L}} = K_{\mathrm{Hg}\mathbf{L}}^{\mathrm{pH2.0}} \times \beta_{\mathrm{H_5L}} \times [\mathrm{H}]^2 \;\; ; \quad \log\beta_{\mathrm{HgH_3L}} = \log K_{\mathrm{Hg}\mathbf{L}}^{\mathrm{pH2.0}} + \log\beta_{\mathrm{H_5L}} - 2 \times \mathrm{pH}$$

The formation constant for $[HgH_3L]^{2+}$ and the protonation constants of the ligand were fixed in the fitting of titration data of the Hg^{II}-containing samples, allowing the determination of the final speciation model and the stabilities of the various complexes present at equimolar or sub-equimolar concentration of Hg^{II} relative to the ligand (see the relevant chapter below).

DFT calculations on the tristhiolate-coordinated As^{III} and Hg^{II} centers of the complexes formed with the studied ligand

Metal site models and geometry optimization

The crystal structure of AfArsR-As1[20] was obtained from the protein data bank[21] via accession number 6J05. The metal site was extracted as a small model system, comprising As^{III}, C^{α}, C^{β}, and S atoms from C102, C95, and C96, along with the backbone nitrogen of C96 and the backbone carbonyl of C95 (i.e., the backbone between C95

and C96). Hydrogens were added to the model with Maestro.[22] All cysteines were in their thiolate form. Two additional variants of this system were prepared. In the first variant As^{III} was replaced with Hg^{II}. In the second variant, the crystal structure coordinates of As^{III} were altered by reflecting the ion through the plane formed by the three cysteine sulfur atoms. This placed As^{III} equidistantly on the opposite side of the plane, at a perpendicular distance of 1.10 Å. Charges of 0 and −1 were assigned to systems with As^{III} and Hg^{II}, respectively. The three systems were subjected to gas-phase DFT geometry optimization with the BP86 functional.[23] Vibrational frequencies were calculated to confirm optimization to a minimum by the absence of imaginary modes. Scalar relativistic effects were included with the Zero-Order Regular Approximation (ZORA).[24] The relativistically recontracted ZORA-def2-TZVP all-electron basis set[25,26] and the decontracted def2/J auxiliary SARC/J basis set[27] were used for non-metal atoms. The segmented all-electron relativistically contracted (SARC) basis set SARC-ZORA-TZVP was used for Hg^{II}.[26] All DFT calculations were carried out with ORCA 5.0.3.[28]

MD simulations

The fragment NCCHGTRDCA was extracted from the AfArsR-As crystal structure (PDB-ID: 6J05) and protonated in Maestro[29] corresponding to neutral pH. N-terminal acetylation and C-terminal amidation were introduced followed by a local geometry optimization of the added atoms. Three variants of the Ac-NCCHGTRDCA-NH_2 peptide were prepared for MD simulations: (A) the system with As^{III} coordination geometry from the crystal structure, (B) the system with planar Hg^{II} coordination geometry from the DFT structure in section "Computational studies" and (C) the metal-free system with the three cysteines protonated. The Hg^{II} system was structurally relaxed with Macromodel[30] using the OPLS3 [31] force field, to minimize strain resulting from merging the DFT and crystal structure coordinates. During relaxation, the DFT optimized coordinates of Hg^{II} and sulfur atoms were fixed, while the remaining atoms were allowed to move freely.

In order to maintain the metal site geometry during MD simulations, Desmond automatically assigned harmonic constraints with force constants of 300 kcal/mol/$Å^2$ for metal ion-sulfur bonds, and 60 kcal/mol/rad^2 for angles involving sulfur and the metal ion. Automatic partial charge assignment by Desmond employed charge-smearing across the atoms, thereby reducing the initial formal charges on metal ions to 0.645 for Hg^{II} and 0.755 for As^{III}, see Table S1.

Table S1. First coordination sphere partial charges

**Atom**	**Partial charge**
Hg	0.645
S (C102)	−0.591
S (C96)	−0.663
S (C95)	−0.648
As	0.755
S (C102)	−0.357
S (C96)	−0.495
S (C95)	−0.435

In preparation for MD simulations, each system was solvated in a 20 Å cubic TIP3P water box with the addition of Na^+ to neutralize charge, see Table S2. MD simulations were carried out in Desmond with the OPLS3 [31] force field and TIP3P [32] water using the default Desmond pre-relaxation protocol and NPT simulation settings at p = 1 bar and T = 300 K. Simulations were run for 2000 ns for each system, and coordinates were saved each 50 ps. MD trajectories were analyzed and visualized with VMD.[33]

Table S2. MD system composition

System name	Description	Total atoms	Counter ions (Na^+)	Water molecules
A	As^{III}	17367	0	5743
B	Hg^{II}	17335	1	5732
C	Metal free	17363	0	5741

Results and Discussion

UV absorption spectroscopy: Titration of the ligand by As^{III} and determination of the As^{III} binding affinity

Evaluation of the UV-monitored As^{III}-titrations of **L** allowed determining the As^{III} binding affinity of the peptide under the applied conditions. The fitting of data from the four studied pH values (pH = 2.0, 4.5, 6.0, 7.5), executed by the PSEQUAD software[3], produced similar apparent stability constants varying in a ca. 1.0 log unit range (with a possible outlier at pH 6.0, see Figure S1), but displaying a slight increasing tendency with pH-increase ($\log K^{pH2.0}$ = 5.43(2), $\log K^{pH7.5}$ = 6.35(3)). In spite the formation of each As–S bonds occurs via condensation reaction with no actual pH-effect[34–36] (at least with the protonated form of the thiol groups), the ionization state of the ligand still seems to have some influence on the binding affinities. It is interesting to compare the apparent stability of **L** (under neutral conditions) with other *i*As^{III} binding thiolates. The determined $\log K^{pH7.5}$ = 6.35 value is on par with the stabilities of the best *i*As^{III} chelating bisthiol compounds, such as 2,3-dimercaptopropan-1-ol (BAL, $\log K^{pH7.0}$ = 6.95),[36] 1,3-dimercaptopropan-2-ol (1,3-DMPP, $\log K^{pH8.1}$ = 6.31),[37] dithioerythritol (DTE, $\log K^{pH8.1}$ = 6.43)),[37] dithiothreitol (DTT, $\log K^{pH7.4}$ = 6.04),[37,38] but notably larger than the affinities determined for tripodal compounds bearing three Cys or d-Pen units grafted on a NTA platform[34] ($\log K^{pH7.0}$ = 5.26 and 3.04, respectively) or for multiple Cys (3 or 4) containing oligopeptides displaying the Cys units in varying positions (although measured at lower temperature) (2-8 °C, $\log K^{pH7.5}$ = 5.37 – 5.88).[39] This demonstrates that the affinity of the metalloid binding segment of the AfArsR protein towards *i*As^{III}, even at the peptide level without any additional stabilizing effect of the protein structure, is reasonably strong.

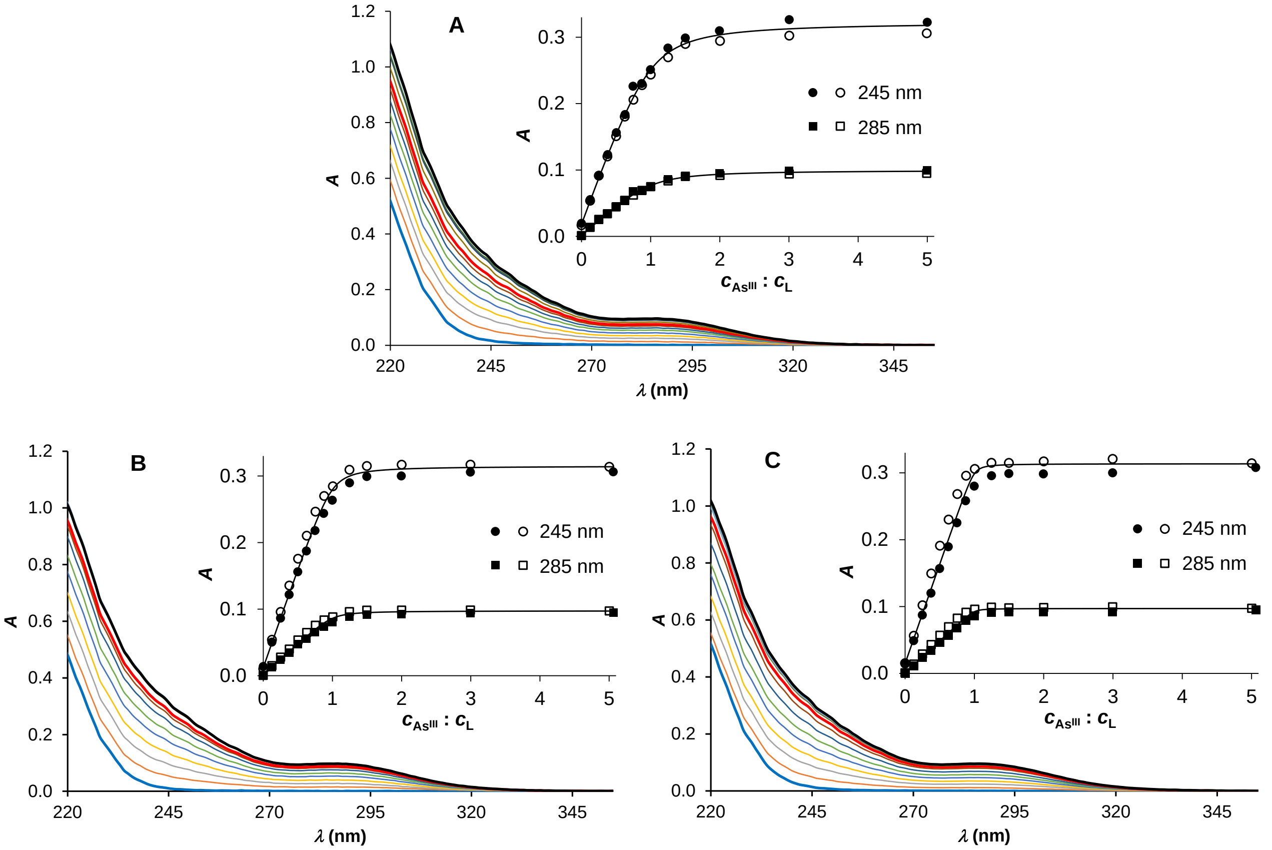

Figure S1. UV-spectra of the ligand at different $c(As^{III}) : c(\mathbf{L})$ ratios at pH = 2.0 (**A**), pH = 4.5 (**B**) and pH = 6.0 (**C**) ($c(\mathbf{L})$ = 50.0 μM). The thick lines are highlighted spectra for 0.0 (blue), 1.0 (red) and 5.0 equiv. (black) of As^{III} per ligand. The inset shows the trend in the change of absorbances at 245 and 285 nm using data from two parallel measurements and the fit of data by the model As^{III} + **L** ⇌ As**L**. A similar figure, displaying data for pH = 7.5, is presented in the main text (Figure 2) (see the details of fitting in the Experimental Procedures). The fitting of data at the four pH values resulted in the following apparent stability constants: $\log K^{pH2.0}$ = 5.43(2), $\log K^{pH4.5}$ = 6.10(4), $\log K^{pH6.0}$ = 6.9(2), $\log K^{pH7.5}$ = 6.35(3).

UV absorption spectroscopy: Titration of the ligand by Hg^{II}

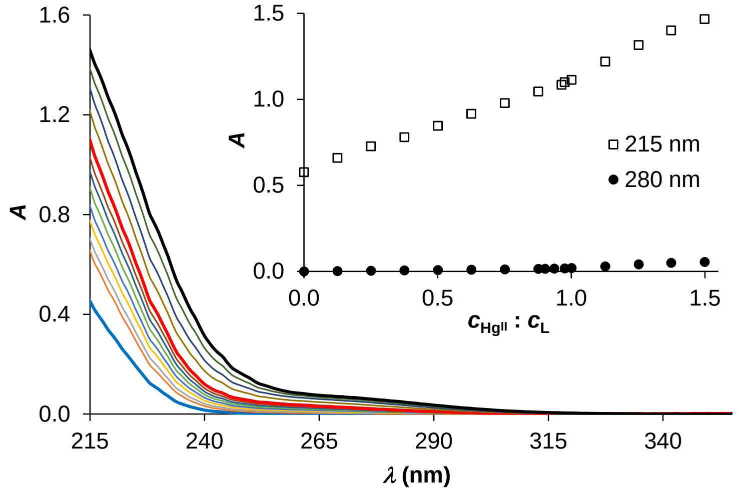

Figure S2. UV-spectra of the ligand at different $c(Hg^{II})$: $c(\mathbf{L})$ ratios at pH = 2.0. The thick lines are highlighted spectra for 0.0 (blue), 1.0 (red) and 1.5 equiv. (black) of Hg^{II} per ligand. The inset shows the trend in the change of absorbances at 215 and 280 nm. ($c(\mathbf{L})$ = 40.0 μM)

Comparison of the pH profiles of the absorbance traces (at 240 and 280 nm – Figure S3.B and C), obtained for **L** without Hg^{II} and in the presence of 0.5 and 1.0 equiv. of Hg^{II}, shows that the plot at 0.5 equiv. of Hg^{II} runs rather close to half way between the other two curves in the whole studied pH-range. Additionally, an extra step between pH ~ 7 and 10 appears on the profile for 0.5 equiv. Hg^{II} per peptide at 240 nm (see Figure S3.B), which may be easily associated with the unbound fraction of the peptide undergoing proton dissociations from the Cys sidechain thiols in the same pH-range, as also seen in the *A* vs. pH profile of the free ligand (triangles). Accordingly, UV-titrations do not indicate the formation of bis-ligand complexes in the Hg^{II}-**L** system, in addition to the differently protonated monocomplexes and the species with 3:2 Hg^{II}:**L** molar ratio, appearing in the presence of Hg^{II} excess over the peptide.

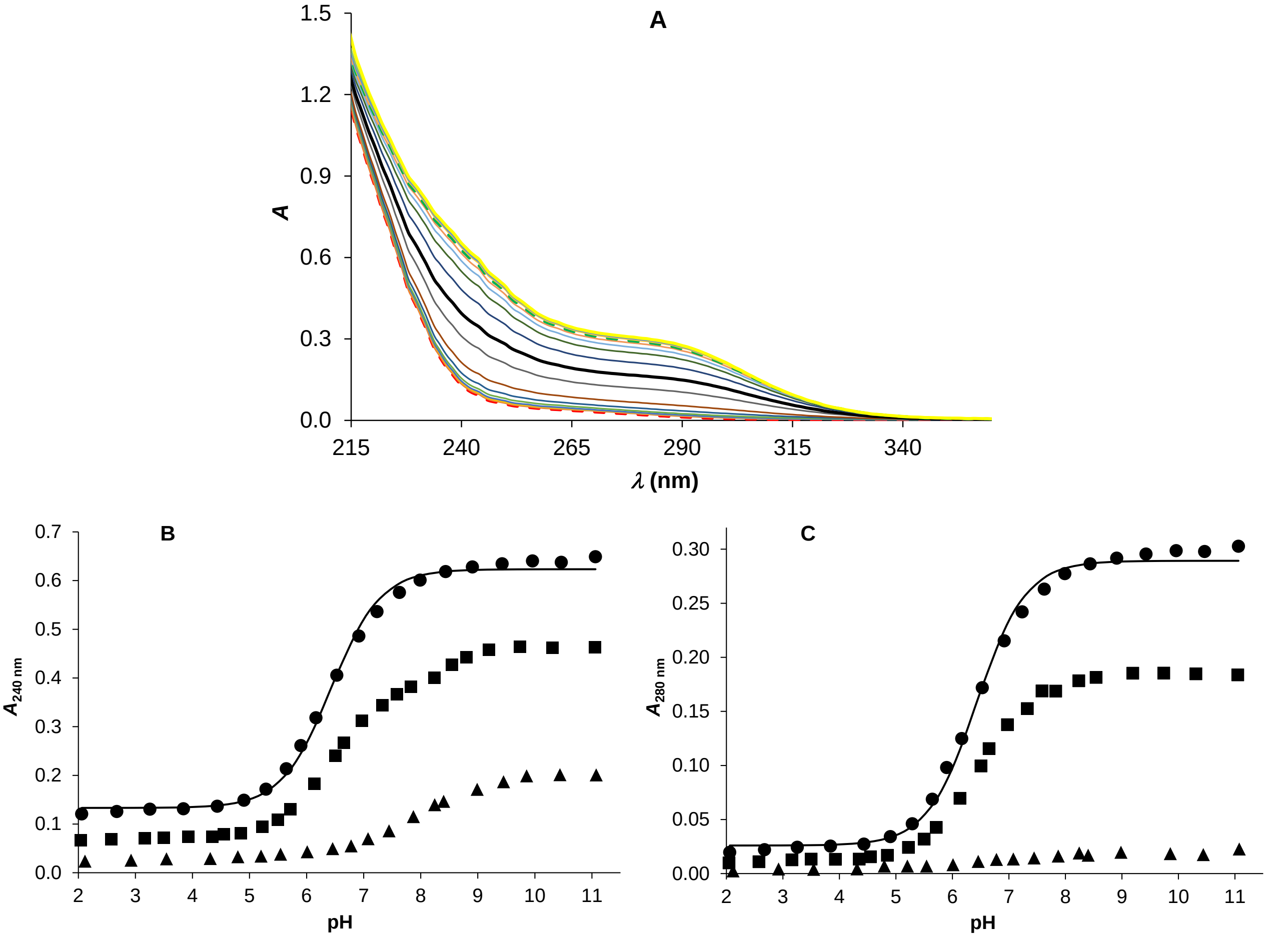

Figure S3. The effect of pH on the UV-spectra of the peptide in the absence and presence of Hg^{II}.
A) pH-dependent series of UV-spectra of **L** recorded for the $c(Hg^{II})$:c(**L**) = 1:1 system. The thick continuous or dashed lines are highlighted spectra for pH = 2.1 (red dashed), 6.5 (black), 8.4 (green dashed) and 11.0 (yellow).
B) and **C**) Absorbances measured at 240 (B) and 280 nm (C) as a function of pH for samples of the ligand containing 0 (triangles), 0.5 (squares) and 1.0 equiv. of Hg^{II} (circles). The continuous lines are fitted curves for the plot of the latter system considering a simple deprotonation process accompanying the binding of the third thiolate group to Hg^{II}: [HgHL] ⇌ $[HgL]^-$ + H^+. The fitted pK_a value for this process is 6.43. (c(**L**) = 40.0 μM)

Hg^{II} binding affinity of the ligand determined by the combination of competition experiments and pH-potentiometric titrations

Considering the well-known outstanding affinity of Hg^{II} to the thiolates of Cys, we conducted an indirect measurement of the stability of the Hg^{II}-**L** monocomplexes, relying on a competition reaction between the excellent Hg^{II} coordinating ligand, iodide ion[4] and the studied peptide, following a previously described protocol[7] (see the Experimental Procedures). The competition reaction, followed by UV spectroscopy (Figure S4), was executed at pH = 2.0 since our preliminary investigations indicated that the apparent stability of the Hg^{II}-peptide complex is so large under neutral conditions that iodide ions cannot displace the Hg^{II}-bound ligand. Indeed, as indicated by panel C) and D) of Figure S4, even at pH = 2.0 a rather large excess of iodide ions is necessary for the completion of the displacement process and the release of the Hg^{II}-coordinated peptide occurs only from ca. 50-fold excess of iodide ions over Hg^{II}. Accordingly, only the tri- and tetraiodido-coordinated Hg^{II}-complexes are formed in the displacement process. The apparent stability constant determined for Hg**L** at pH = 2.0, according to the equations presented in the Experimental Procedures, is $\log K^{pH2.0}$ = 26.81(1). This value is very close to those reported for various cyclic and linear short peptides with cysteines included in a CXCXC pattern ($\log K^{pH2.0}$ = 27.0 – 27.5)[7] These latter apparent stabilities correspond to species with Hg^{II} bound in a $\{HgS_2\}$ coordination environment, as proposed by stability data reported for the Hg^{II} binding of two Cys-containing peptides: (i) the stability constant of the Hg^{II} complex of Ac-Cys-dPro-Pro-Cys-NH_2 when extrapolated to pH 2.0 ($\log K'$ = 26.2)[40] or (ii) the stability determined for the Hg^{II} complex of a flexible, loop forming peptide with Cys residues in positions 2 and 10 in the amino acid sequence, $\log K'$ = 25.7).[41]

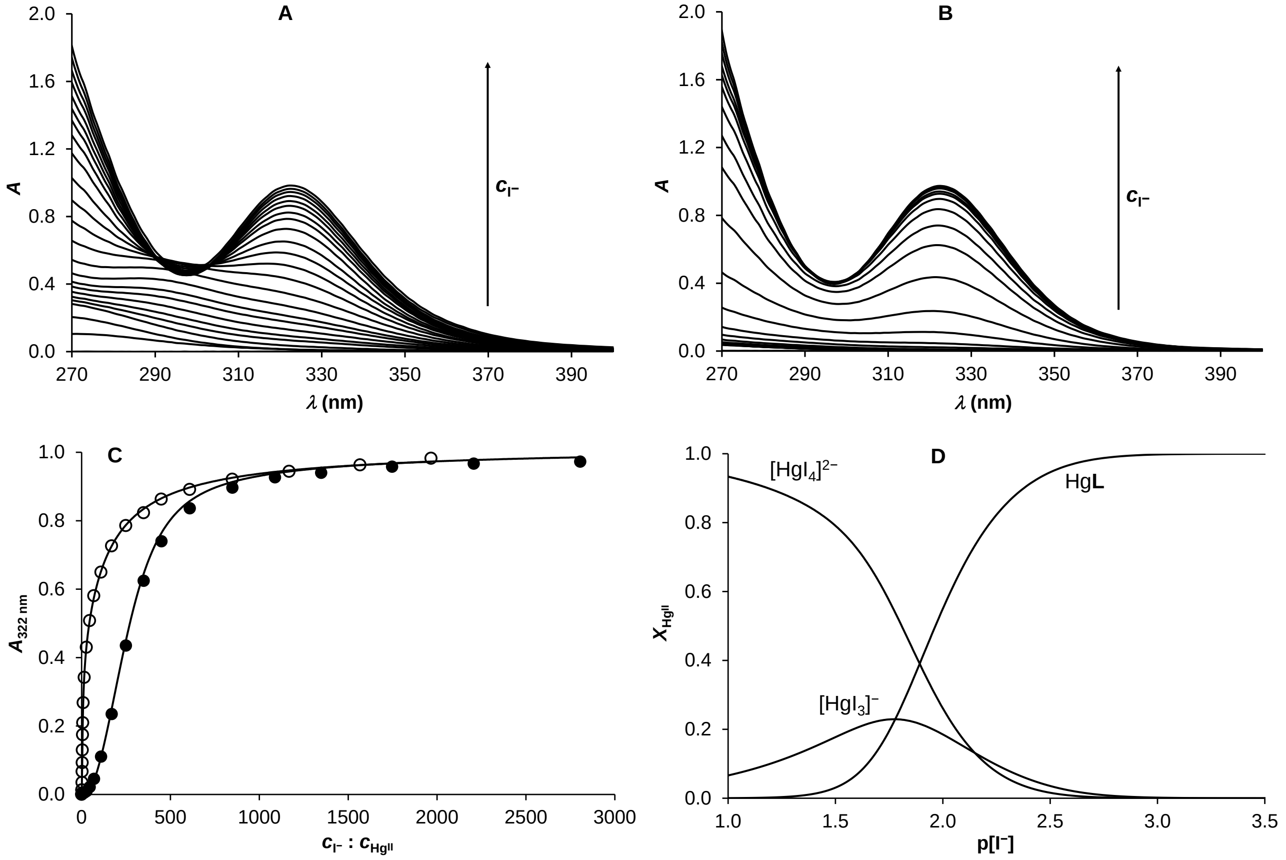

Figure S4. Displacement of the Hg^{II}-bound peptide by iodide ions at pH = 2.0.

A) and **B**) Series of UV-spectra in the titration of Hg^{II} by iodide ions in the absence (A) and presence of **L** (B). ($c(Hg^{II})$ = 50.0 μM, c(**L**) = 1.1×$c(Hg^{II})$ = 55.0 μM).

C) Measured and fitted absorbance traces at 322 nm from the UV-titrations (presented in panels A) and B)) performed in the absence (open circles) and presence of the ligand (filled circles). Data were evaluated as described in the Experimental Procedures.

D) Distribution of species in the Hg^{II} – **L** – I^- system as a function of p[I^-], representing the complex equilibria in the course of the displacement process.

pH-potentiometric titrations were also performed to describe the complex speciation in the Hg^{II} – **L** system allowing the calculation of thermodynamic overall formation constants of the individual complexes (Table S3), relying on the apparent stability constant determined separately at pH 2.0 (Figure S5, detailed explanation is provided in the Experimental Procedures). The five determined pK_a values of the free peptide, in increasing order, can be attributed to the deprotonations of the Asp, His and the three Cys sidechain groups (the pK_a of the Arg guanidine group was too high to be determined). The titration curves obtained in the presence of Hg^{II} indicate that complexation processes take place already under acidic conditions. Samples containing Hg^{II} and the peptide at three different concentration ratios (see Figure S5.A) were titrated without any sign of precipitate formation, suggesting that oligomeric structures, assuming the simplest 3:2 composition of Hg^{II} and **L**, must be present in the samples, especially at Hg^{II} excess over **L**. The presence of such complexes, possibly displaying interligand Hg^{II}-bridges between thiolate groups of two ligands, was also strongly supported by UV-Vis and CD spectroscopic experiments, as well as by ESI-MS. At larger Hg^{II}-excess (2-fold) precipitate appeared in the studied samples, which either suggests the formation of insoluble complexes with higher than 1.5 metal-to-ligand ratio, or the hydrolysis of the non-coordinated metal ions.

The linearly increasing trend of absorbances observed in between 1:1 and 1.5:1 Hg^{II}:**L** concentration ratios at pH 2.0 (Figure S2) clearly indicates that monomeric species are fully transformed into trinuclear structures, independently of the pH of the sample (see also Figure 3 in the main text). Therefore, stability constants for these trinuclear species cannot be reliably calculated if additional independent data are not available from other type of experiments. However, such independent data was available only for the monocomplexes from the competition studies, as described above. Accordingly, the titration curve of the sample containing 1.4-fold Hg^{II} excess over the ligand was necessarily omitted from data fitting and thus the model was restricted to species that describe the speciation only up to a 1:1 Hg^{II}:**L** concentration ratio. It must be noted, however, that the presence of trinuclear species, especially in experiments where the components are used in a concentration near 1 mM, may have some influence on the observed data.

Table S3. Logarithmic overall formation constants (logβ) for the protonation and Hg^{II}-complexation processes of the ligand (with errors in the last digits in parentheses) and calculated pK_a values for the deprotonation processes of the species (I = 0.1 M $NaClO_4$, T = 298 K).[a]

Species	logβ	pK_a (ligand)
$[H_5L]^{2+}$	35.83(3)	3.67
$[H_4L]^{+}$	32.16(2)	6.33
$[H_3L]$	25.83(2)	7.95
$[H_2L]^{-}$	17.88(1)	8.60
$[HL]^{2-}$	9.28(1)	9.28
		pK_a (HgH_qL_r) [b]
$[HgH_3L]^{2+}$ [c]	58.64(fixed)	3.61
$[HgH_2L]^{+}$	55.03(3)	5.67
[HgHL]	49.36(5)	6.96
$[HgL]^{-}$	42.40(6)	–

[a] Number of fitted data points and the fitting parameter (σ) in the evaluation of the Hg^{II}-ligand titration curves by Hyperquad[18] were 212 and 0.48, respectively

[b] pK_a (HgH_qL_r) = logβ (HgH_qL_r) – logβ ($Hg_pH_{q-1}L_r$)

[c] The formation constant of this species was derived from the apparent stability constant for the Hg^{II}-binding of the ligand at pH = 2.0 as described in the Experimental Procedures: logβ(HgH_3L) = log$K^{pH2.0}$ + logβ(H_5L) – 2×pH = 26.81 + 35.83 – 2×2.0 = 58.64. This value was then fixed in the data fitting process.

The involvement of bis-ligand species (with two peptides binding to one metal ion) in the model resulted in a somewhat reduced fitting parameter. Nevertheless the match between the measured and calculated pH values in the slightly alkaline pH-regime, where the Cys residues of the non-bound ligand also dissociate without metal ion assistance, was only little improved. Therefore, the formation of such species was ambiguous based on the pH-potentiometric data and it was not supported either by any other applied spectroscopic techniques. Bis-ligand complexes were thus omitted from the final, accepted speciation model, nevertheless, the existence of such species in a small quantity cannot be fully neglected.

Evaluation of titrations of the peptide containing Hg^{II} in subequimolar or equimolar concentrations, by applying a fixed logβ value for the $[HgH_3L]^{2+}$ species (see the footnote of Table S3), led to a model consisting of differently protonated monomeric species (Figure S5). These curves can be described by the consecutive formation of the monocomplexes $[HgH_3L]^{2+}$, $[HgH_2L]^{+}$, [HgHL] and $[HgL]^{-}$ (charges are omitted in the speciation diagram). The pK_a values, characterizing the transformations of the consecutively formed monomeric complexes, are assigned to the dissociation of protons from the Asp, His and the previously unbound third Cys residue. The lowest pK_a value (3.61) is nearly identical to the first pK_a of the free ligand confirming that the dissociation of the Asp carboxyl group takes place without metal ion assistance. There is a notable drop in the subsequent two pK_a values, as compared to those characterizing the proton release of the His and one of the Cys units in the unbound ligand. Indeed, the dissociation of the third Cys thiol is promoted by Hg^{II}-coordination, as proposed by other spectroscopic findings (see the main text), and it notably overlaps with the deprotonation of the His sidechain imidazole. [HgHL] very likely exists in two different micro-protonation states, one with a deprotonated His and a protonated, unbound Cys thiol and another one with a protonated His and a deprotonated, coordinating Cys thiolate group. This is also supported by the spectroscopically (UV-Vis) observed Hg^{II}-induced dissociation of the third Cys residue, reflecting a pK_a (~6.4) that falls in between the two values observed by pH-potentiometry for the two consecutive deprotonation steps, leading from $[HgH_2L]^{+}$ to $[HgL]^{-}$ (5.7 and 7.0) – see also Figure S3.A-C and the main text.

In summary, the observed pK_a values characterizing the deprotonation and interconversion of the monocomplexes together with the results of solution structural studies (see the main text) show that $[HgH_3L]^{2+}$ and $[HgH_2L]^{+}$ involve two Hg^{II}-bound thiolates whereas the metal center is coordinated by all the three Cys sidechains in $[HgL]^{-}$. [HgHL] most likely exists in isomeric structures with either two or three Hg^{II}-bound thiolates. In other words, the $\{HgS_2\}$ type species, present in the acidic/slightly acidic pH regime, transforms between pH ~ 5.5 – 7.5 into the parent monocomplex with the $\{HgS_3\}$ coordination mode, dominating above pH ~ 8 (Figure S5.B).

Based on the determined overall formation constants, the apparent stability for Hg^{II} binding at pH 7.5 was also calculated: log$K^{pH7.5}$ = 39.0. This value is ca. two orders of magnitude larger than the similar extrapolated data for the Hg^{II} complex of Ac-Cys-dPro-Pro-Cys-NH_2 (log$K^{pH7.5}$ = 37.2)[40] and by the same margin falls short of the stabilities of some deca/nonapeptides with a CXCXC motif, *vide supra* (log$K^{pH7.4}$ = 40.0 – 41.0).[7] This shows that the binding of the third thiolate in this specific amino acid sequence only moderately stabilizes the already extremely strong Hg^{II} binding. On the other hand, the nearly 33 orders of magnitude difference in the affinity of the AfArsR model peptide in favor of Hg^{II} over As^{III} implies that it is highly improbable that affinity plays a decisive role in recognizing the appropriate effector by the metalloregulator and the distinction of metalloids from other thiophilic metal ions, including Hg^{II}.

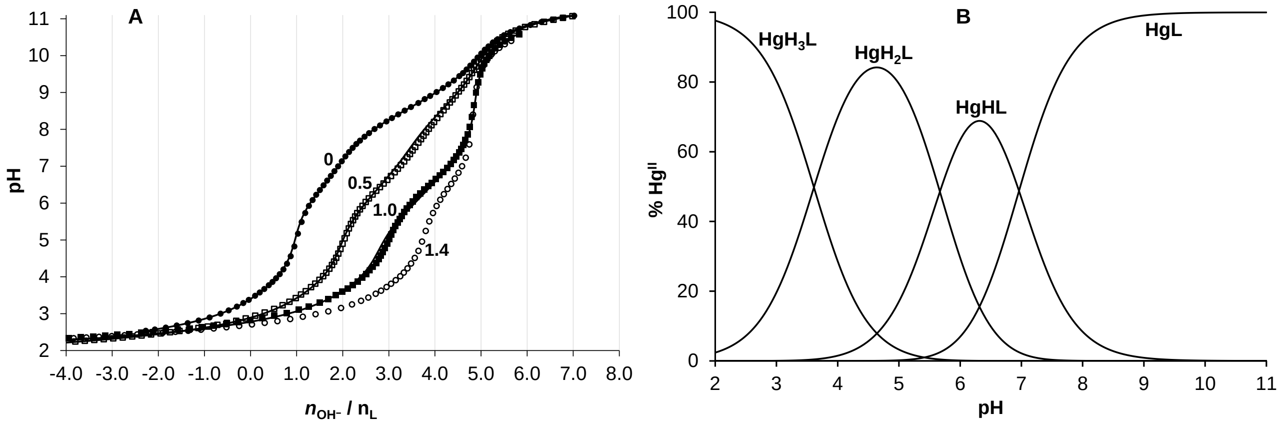

Figure S5. Titration curves of **L** in the absence and presence of Hg^{II}, and speciation diagram calculated based on the stability constants obtained by evaluating the titration data (see the relevant section of Experimental Procedures and Table S3).

A) Titration curves of **L** with 0 (filled circles), 0.5 (open squares), 1.0 (filled squares) and 1.4 equiv. of Hg^{II} (open circles) in a form of consumed base equivalents per ligand after subtracting the base consumption for the free strong acid content of the samples (c(**L**) varied in the range of 9.2×10^{-4} – 1.3×10^{-3} M).

B) Species distribution curves calculated for 1:1 Hg^{II}:**L** concentration ratio with c(**L**) = 5.0×10^{-5} M.

Circular dichroism (CD) spectroscopy

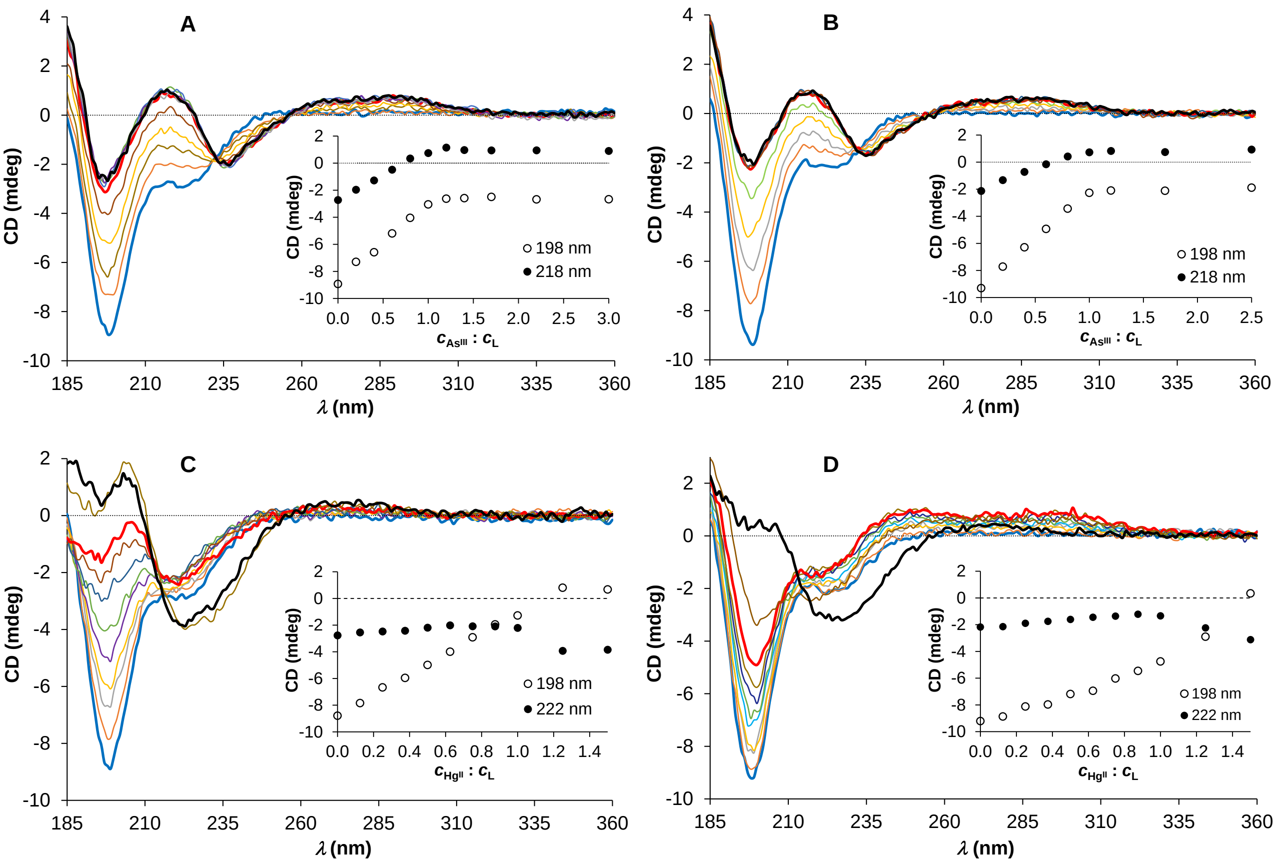

Figure S6. Titration of the ligand by As^{III} or Hg^{II} at different pH values (c(**L**) = 650 µM, l = 0.1 mm), followed by CD spectroscopy. The pH of the samples was set by perchloric acid (c = 10 mM, pH = 2.0) or phosphate buffer (c = 20 mM, pH = 7.5).

A) and **B**) CD-spectra of the ligand at increasing $c(As^{III}) : c$(**L**) ratios recorded at pH = 2.0 (**A**) and pH = 7.5 (**B**). The thick lines are highlighted spectra for 0.0 (blue), 1.0 (red) and 3.0/2.5 equiv. (A/B) (black) of As^{III} per ligand.

C) and **D**) CD-spectra of the ligand at increasing $c(Hg^{II}) : c$(**L**) ratios recorded at pH = 2.0 (**C**) and pH = 7.5 (**D**). The thick lines are highlighted spectra for 0.0 (blue), 1.0 (red) and 1.5 equiv. (black) of Hg^{II} per ligand.

ESI-MS data characterizing As^{III} and Hg^{II} binding by the ligand

ESI-MS spectra were recorded with an aim to provide support for the speciation models, proposed by UV and CD spectroscopy and pH-potentiometry for the As^{III}–**L** and/or Hg^{II}–**L** systems, but also to potentially exploit information on the presence of binding isomers of the {HgS_2} type species present under acidic conditions. The spectra obtained at pH = 7.8 for As^{III}:**L** at any applied concentration ratios show the exclusive formation of adducts corresponding to the simple monocomplex, also indicated by all other methods (Figure S7).

Experiments performed with Hg^{II} at different Hg^{II}:**L** concentration ratios confirm the formation of the monocomplex (Figure S8 and Figure S9.A) but also the presence of species with a 3:2 Hg^{II}:**L** molar ratio (Figure S9.A), in line with the results of other techniques. Our attempt to collect information for possible bis-ligand type species was not successful since the spectra recorded at pH = 9.1 with a substantial molar excess of **L** over Hg^{II} (4:1 concentration ratio) did not show any sign of the presence of such complexes (Figure S8). MS-MS spectra were also recorded at pH 3.0 at 1.0 equiv. of Hg^{II} per ligand to monitor the fragments obtained for the ion with most intense peak at *m/z* value characteristic for the simple monocomplex (Figure S9.B-C). The fragmentation pattern indicates the presence of several type of Hg^{II}-bound molecule ions that form if amide bonds in between ^{3}Cys and ^{9}Cys are cleaved. However, neither of the possible fragments that would form if the cleavage takes place between ^{2}Cys and ^{3}Cys was detected. While this finding does not fully exclude the presence of species with Hg^{II} bridging the ^{2}Cys or ^{3}Cys thiolate with that of ^{9}Cys, it suggests that, under the conditions of ESI-MS, coordination of Hg^{II} to the two neighboring thiolates occurs with a notably higher probability (Figure S9.B-C).

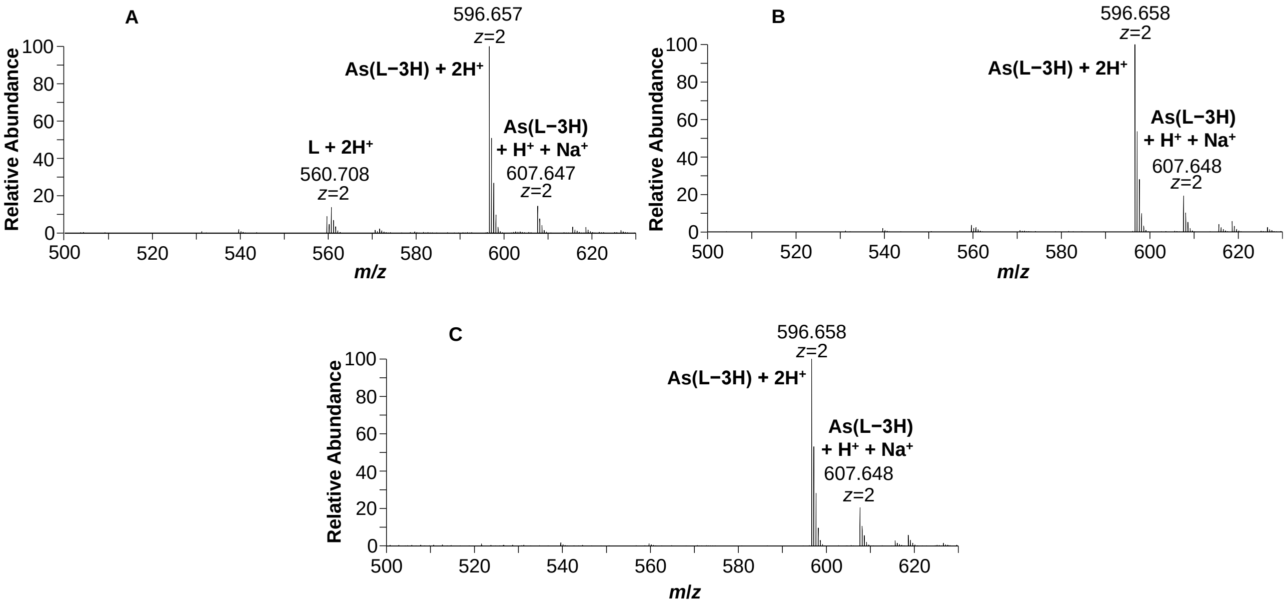

Figure S7. ESI-MS spectra recorded for the ligand with 0.5 (**A**), 1.0 (**B**) and 4.0 equiv. (**C**) of As^{III} at pH = 7.8 in positive ion mode. (c(**L**) = 65 μM, $c(NH_4HCO_3)$ = 10 mM)

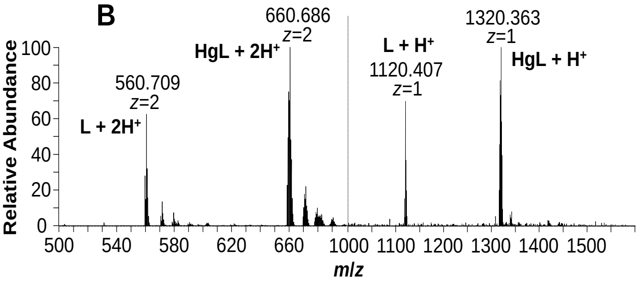

Figure S8. Two segments of the ESI-MS spectrum recorded for the ligand with 0.25 equiv. of Hg^{II} at pH = 9.1 (B) in positive ion mode. (c(**L**) = 65 μM, $c(NH_4HCO_3)$ = 10 mM)

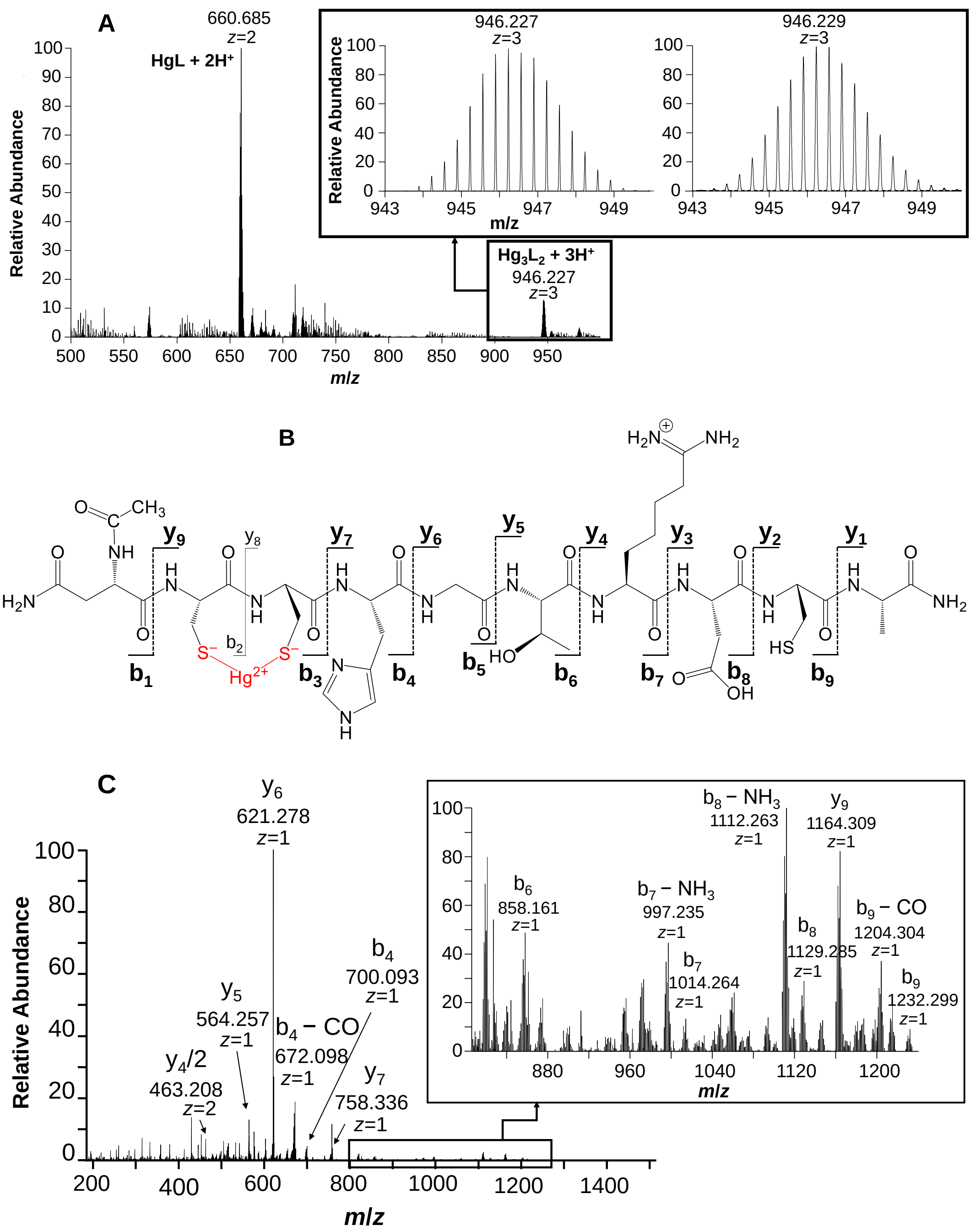

Figure S9. ESI-MS information about various Hg^{II}-bound species.

A) ESI-MS spectrum recorded for the $c(Hg^{II})$:$c(\mathbf{L})$ = 1.5:1 system (pH = 7.9, $c(NH_4HCO_3)$ = 10 mM, $c(\mathbf{L})$ = 65 µM). The framed box presents the measured (left) and simulated (right) isotope pattern for the peak at m/z = 946.23, attributed to the triple-charged form of the trinuclear Hg^{II}-complex.

B) and **C**) Fragmentation scheme in the CID (collision-induced dissociation) analysis of the peptide with 1.0 equiv. of Hg^{II} (**B**) and the MS-MS spectrum (**C**) showing the assigned fragments obtained for the ion with the most intense peak at m/z = 660.69 (z = 2) in the MS spectrum of the $c(Hg^{II})$:$c(\mathbf{L})$ = 1:1 system at pH = 3.0 (c(Ac-OH) = 10 mM, $c(\mathbf{L})$ = 65 µM). The indicated 'b' fragments, except 'b_1', are all Hg^{II}-adducts, while 'y' fragments, with the exception of 'y9', are Hg^{II}-free ions.

The observed fragmentation pattern indicates the absence of both 'b_2' and 'y_8' that would form if the backbone was cleaved between ^{2}Cys and ^{3}Cys. At the same time, most of the other possible Hg^{II}-bound 'b', as well as the Hg^{II}-free 'y' fragments are present indicating that species with Hg^{II}-bridging the thiolates of ^{2}Cys and ^{9}Cys or ^{3}Cys and ^{9}Cys are not prominent. These findings suggest that at low pH (= 3.0), at least under the conditions of the MS studies, the ^{2}Cys and ^{3}Cys bound HgS_2-type monocomplex isomer dominates over the other two possible bisthiolate-coordinated isomeric structures.

^{1}H NMR spectroscopy

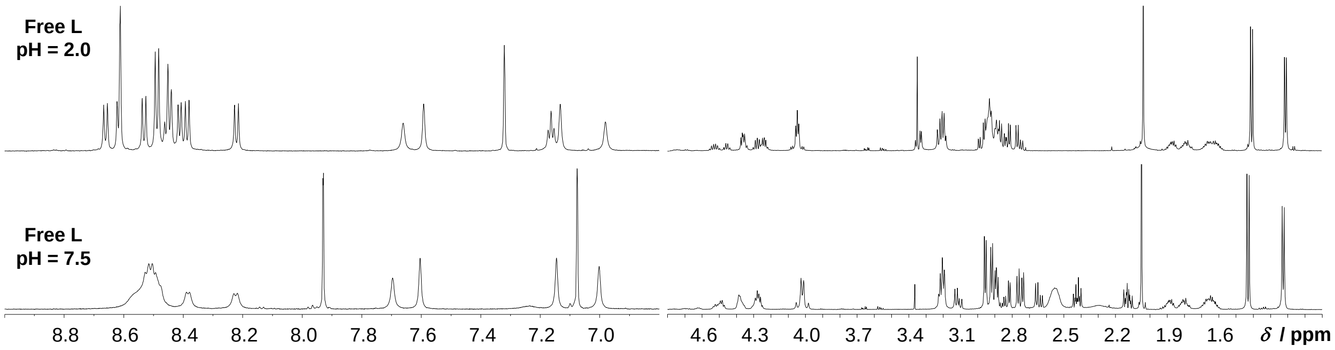

Figure S10. ^{1}H NMR spectra of **L** recorded in H_2O/D_2O 90/10 *V*/*V* % at pH = 7.5 (bottom) and 2.0 (top) (*T* = 282 K, *c*(**L**) = 1.0 mM). Note that the sample at pH = 7.5 contained the reducing agent TCEP to maintain the reduced (thiol) form of the peptide (*c*(TCEP) = 1.0 mM). The sharp peak between 3.3 – 3.4 ppm is a resonance of a small amount of methanol.

Table S4. Assignment of the ^{1}H NMR (600 MHz) chemical shifts (δ / ppm) of the proton resonances of Ac-NCCHGTRDCA-NH_2 (**L**) at *T* = 282 K at pH = 7.5 (*c*(**L**) = 1×10^{-3} M, *I* = 0.1 M $NaClO_4$, H_2O/D_2O 90/10 V/V %). The sample contained TCEP ($c = 1\times10^{-3}$ M) in order to maintain the reduced (thiol) form of the peptide.

pH = 7.5	NH	$C^{\alpha}H$	$C^{\beta}H_2$	Other resonances
^{1}Asn	8.52	4.70	2.75, 2.83	$N^{\delta2}H_2$: 7.00, 7.69
^{2}Cys	8.54 / 8.57	4.48 / 4.52	2.89 / 2.92	
^{3}Cys	8.54 / 8.57	4.48 / 4.52	2.89 / 2.92	
^{4}His		4.69	3.11, 3.21	$C^{\delta2}H$: 7.08, $C^{\varepsilon1}H$: 7.93
^{5}Gly	8.54	4.03 [a]		
^{6}Thr	8.22	4.38	4.27 [b]	$C^{\gamma2}H_3$: 1.22
^{7}Arg	8.51	4.37	1.80, 1.89	$C^{\gamma}H_2$: 1.65, $C^{\delta}H_2$: 3.21
^{8}Asp	8.50	4.62	2.65, 2.75	
^{9}Cys	8.38	4.50	2.95	
^{10}Ala	8.48	4.28	1.43 [c]	
Protecting groups				CO-CH_3: 2.05, CO-NH_2: 7.15, 7.60

[a] Chemical shift for $C^{\alpha}H_2$ of Gly. [b] Chemical shift for $C^{\beta}H$ of Thr. [c] Chemical shift for $C^{\beta}H_3$ of Ala.

Table S5. Assignment of the ^1H NMR (600 MHz) chemical shifts (δ / ppm) of the proton resonances of Ac-NCCHGTRDCA-NH_2 (**L**) at *T* = 282 K at pH = 2.0 (c(**L**) = 1×10^{-3} M, *I* = 0.1 M $NaClO_4$, H_2O/D_2O 90/10 V/V %).

pH = 2.0	NH	$C^{\alpha}H$	$C^{\beta}H_2$	Other resonances/
^{1}Asn	8.49	4.69	2.75, 2.83	$N^{\delta 2}H_2$: 6.99, 7.67
^{2}Cys	8.53	4.54	2.93	
^{3}Cys	8.49	4.45	2.89	
^{4}His	8.66	4.76	3.34, 3.20	$C^{\delta 2}H$: 7.32 $C^{\varepsilon 1}H$: 8.61
^{5}Gly	8.46	4.04 [a]		
^{6}Thr	8.22	4.36	4.24 [b]	$C^{\gamma 2}H_3$: 1.21
^{7}Arg	8.45	4.36	1.79, 1.86	$C^{\gamma}H_2$: 1.64, $C^{\delta}H_2$: 3.20<br>$N^{\delta 2}H_2$: 7.16
^{8}Asp	8.61	4.73	2.85, 2.97	
^{9}Cys	8.39	4.52	2.93	
^{10}Ala	8.41	4.28	1.43 [c]	
Protecting groups				CO-CH_3: 2.04, CO-NH_2: 7.13, 7.59

[a] Chemical shift for $C^{\alpha}H_2$ of Gly. [b] Chemical shift for $C^{\beta}H$ of Thr. [c] Chemical shift for $C^{\beta}H_3$ of Ala.

Table S6. Assignment of the ^1H NMR (600 MHz) chemical shifts (δ / ppm) of the proton resonances of Ac-NCCHGTRDCA-NH_2 (**L**) at T = 282 K in the presence of 1.5 equiv. of As^{III} at pH = 7.5 (c(**L**) = 1×10^{-3} M, $c(As^{III})$ = 1.5×10^{-3} M, I = 0.1 M $NaClO_4$, H_2O/D_2O 90/10 V/V %). Assigned resonances of a minor species are depicted in italic in parentheses. Experiments were executed with and without TCEP (c = 1×10^{-3} M) in the samples. By this procedure, we could verify that the reduced (thiol) form of the peptide is maintained but it also allowed avoiding interferences with the overlapping signals at the reducing agent.

pH = 7.5	NH	$C^{\alpha}H$	$C^{\beta}H_2$	Other resonances/
^{1}Asn	8.47<br>*(8.50)*	4.67<br>*(4.80)*	2.67, 2.78<br>*(2.65, 3.00)*	$N^{\delta2}H_2$: 6.95, 7.65<br>*($N^{\delta2}H_2$: 7.67, 6.95)*
^{2}Cys		4.37<br>*(4.61 / 4.78)* [d]	3.15, 3.46<br>*(3.47, 3.57 / 3.54, 3.68)* [d]	
^{3}Cys		4.23<br>*(4.61 / 4.78)* [d]	3.55, 3.68<br>*(3.47, 3.57 / 3.54, 3.68)* [d]	
^{4}His	8.75	4.26<br>*(4.61 / 4.78)* [d]	3.38, 3.42<br>*(3.47, 3.57 / 3.54, 3.68)* [d]	$C^{\delta2}H$: 7.07, $C^{\varepsilon1}H$: 8.01<br>*($C^{\delta2}H$: 7.04, $C^{\varepsilon1}H$: 7.86)*
^{5}Gly	8.14	3.96, 4.23 [a]<br>*(3.79, 4.01)*		
^{6}Thr	8.08<br>*(8.03)*	4.40<br>*(4.41)*	4.28 [b]<br>*(4.29)*	$C^{\gamma2}H_3$: 1.32<br>*($C^{\gamma2}H_3$: 1.22)*
^{7}Arg	7.42<br>*(8.35)*	4.49<br>*(4.38)*	1.79, 1.95<br>*(1.84, 1.93)*	$C^{\gamma}H_2$: 1.53, 1.35, $C^{\delta}H_2$: 3.18, $N^{\delta2}H_2$: 7.10<br>*($C^{\gamma}H_2$: 1.64, $C^{\delta}H_2$: 3.23)*
^{8}Asp	8.62<br>*(8.26)*	4.83<br>*(4.76)*	2.58, 2.69<br>*(2.58, 2.72)*	
^{9}Cys	9.09<br>*(8.38)*	4.81<br>*(4.59)*	3.16, 3.43<br>*(3.48, 3.18)*	
^{10}Ala	8.50	4.26	1.42 [c]	
Protecting groups				CO-CH_3: 2.02, CO-NH_2: 7.11, 7.70<br>*(CO-CH_3: 2.03, CO-NH_2: 7.13, 7.59)*

[a] Chemical shift for $C^{\alpha}H_2$ of Gly. [b] Chemical shift for $C^{\beta}H$ of Thr. [c] Chemical shift for $C^{\beta}H_3$ of Ala. [d] These resonance sets could not be assigned to the individual Cys or His residues of the minor component(s).

Table S7. Assignment of the ^{1}H NMR (600 MHz) chemical shifts (δ/ ppm) of the proton resonances of Ac-NCCHGTRDCA-NH2 (**L**) at T = 282 K in the presence of 1.5 equiv. of As^{III} at pH = 2.0 (c(**L**) = 1×10^{-3} M, $c(As^{III})$ = 1.5×10^{-3} M, I = 0.1 M $NaClO_4$, H_2O/D_2O 90/10 V/V %). Assigned resonances of a minor species are depicted in italic in parentheses. Experiments were executed with and without TCEP (c = 1×10^{-3} M) in the samples. By this procedure, we could verify that the reduced (thiol) form of the peptide is maintained but it also allowed avoiding interferences with the overlapping signals at the reducing agent.

pH = 2.0	NH	C$^{\alpha}$H	C$^{\beta}$H$_2$	Other resonances/
^{1}Asn	8.39<br>*(8.46)*	4.65<br>*(4.76)*	2.66, 2.78<br>*(2.67, 2.92)*	N$^{\delta2}$H$_2$: 6.93, 7.59<br>*(N$^{\delta2}$H$_2$: 7.61, 6.94)*
^{2}Cys	8.72<br>*(8.56)*	4.35<br>*(4.65)*	3.47, 3.12<br>*(3.47, 3.53)*	
^{3}Cys	9.87<br>*(8.88 / 8.35)* [d]	4.24<br>*(4.53 / 4.58)* [d]	3.54, 3.65<br>*(3.26, 3.31 / 3.18, 3.45)* [d]	
^{4}His	8.84<br>*(8.88 / 8.35)* [d]	4.28<br>*(4.53 / 4.58)* [d]	3.46, 3.53<br>*(3.26, 3.31 / 3.18, 3.45)* [d]	C$^{\delta2}$H: 7.30; C$^{\varepsilon1}$H: 8.60<br>*(C$^{\delta2}$H: 7.32; C$^{\varepsilon1}$H: 8.61)*
^{5}Gly	8.03<br>*(8.69)*	3.95, 4.22 [a]<br>*(3.85, 4.09)*		
^{6}Thr	8.00<br>*(7.97)*	4.37<br>*(4.35)*	4.25 [b]<br>*(4.29)*	C$^{\gamma2}$H$_3$: 1.30<br>*(C$^{\gamma2}$H$_3$: 1.23)*
^{7}Arg	7.40<br>*(8.37)*	4.45<br>*(4.31)*	1.78, 1.95<br>*(1.86, 1.94)*	C$^{\gamma}$H$_2$: 1.30, 1.50; C$^{\delta}$H$_2$: 3.16;<br>N$^{\delta2}$H$_2$: 7.03<br>*(C$^{\gamma}$H$_2$: 1.62; C$^{\delta}$H$_2$: 3.21; N$^{\delta2}$H$_2$: 7.18)*
^{8}Asp	8.74<br>*(8.32)*	4.92<br>*(4.84)*	2.77, 2.91<br>*(2.78, 2.94)*	
^{9}Cys	9.14<br>*(8.88 / 8.35)* [d]	4.81<br>*(4.53 / 4.58)* [d]	3.14, 3.41<br>*(3.26, 3.31 / 3.18, 3.45)* [d]	
^{10}Ala	8.36<br>*(8.84)*	4.26<br>*(4.27)*	1.40 [c]<br>*(1.42)*	
Protecting groups				CO-CH$_3$: 2.01, CO-NH$_2$: 7.12, 7.67<br>*(CO-CH$_3$: 2.03; CO-NH$_2$: 7.56, 7.12)*

[a] Chemical shift for C$^{\alpha}$H$_2$ of Gly. [b] Chemical shift for C$^{\beta}$H of Thr. [c] Chemical shift for C$^{\beta}$H$_3$ of Ala. [d] These resonance sets could not be assigned to the individual Cys or His residues of the minor component(s).

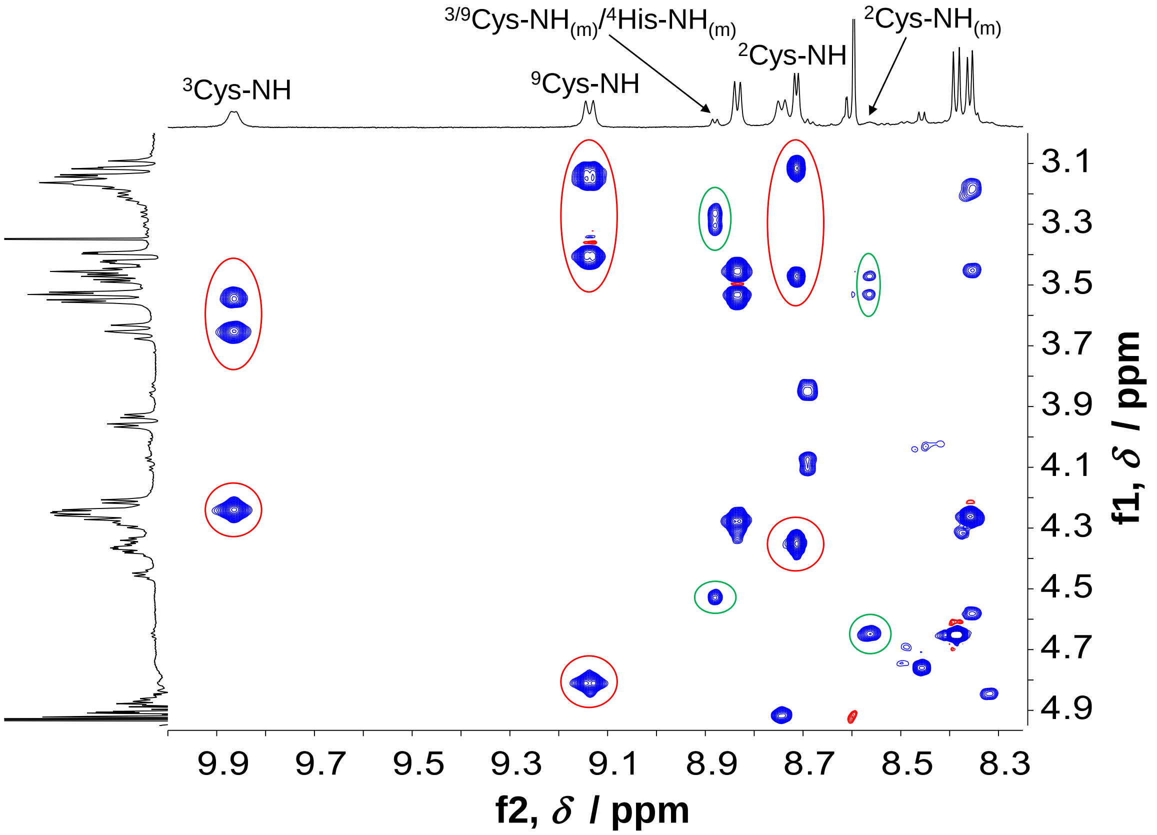

Figure S11. ^1H-^1H TOCSY NMR spectrum of **L** in the presence of 1.5 equiv. of As^{III}, recorded in H_2O/D_2O 90/10 *V*/*V* % at pH = 2.0 (*T* = 282 K, *c*(**L**) = 1.0 mM). $Cys^{\beta}H_2$ – NH and $Cys^{\alpha}H$ – NH crosspeaks of the dominant As^{III}-**L** complex are marked by red circles while similar crosspeaks of a minor species are marked by green circles.

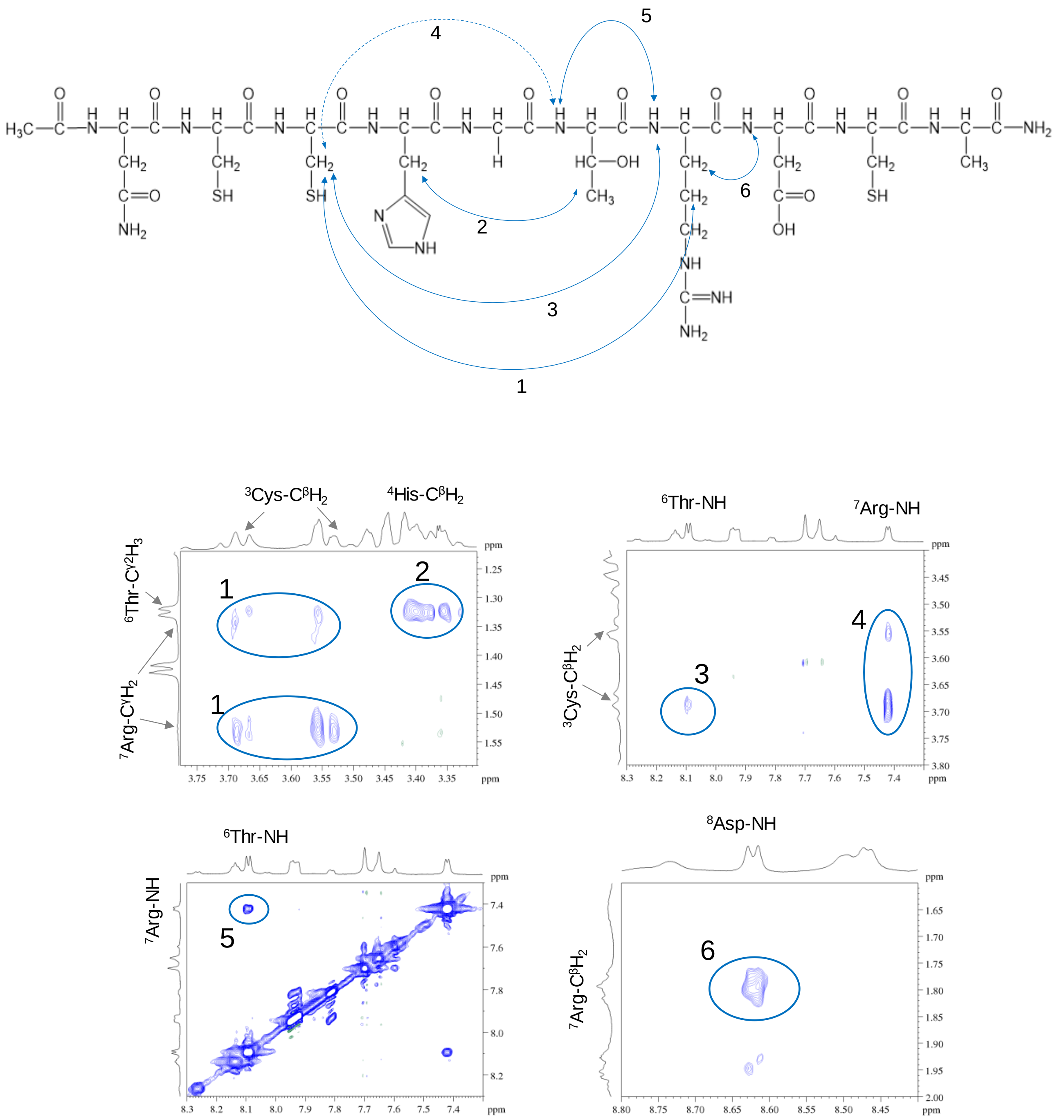

Figure S12. Detected long- and medium-range NOE interactions indicated on the peptide structure by arrows (top), and corresponding cross peaks of the ^1H-^1H NOESY spectra (bottom). The presented NOESY spectra were measured at 282 K for an As^{III}–**L** sample containing As^{III} and the peptide in a 1:1 concentration ratio at pH = 7.5 and using a mixing time of 200 ms (c(**L**) = 3.0 mM). The cross peaks could be detected only in the presence of As^{III}, the free ligand displays a random coil structure without any medium- or long-range NOE interactions.

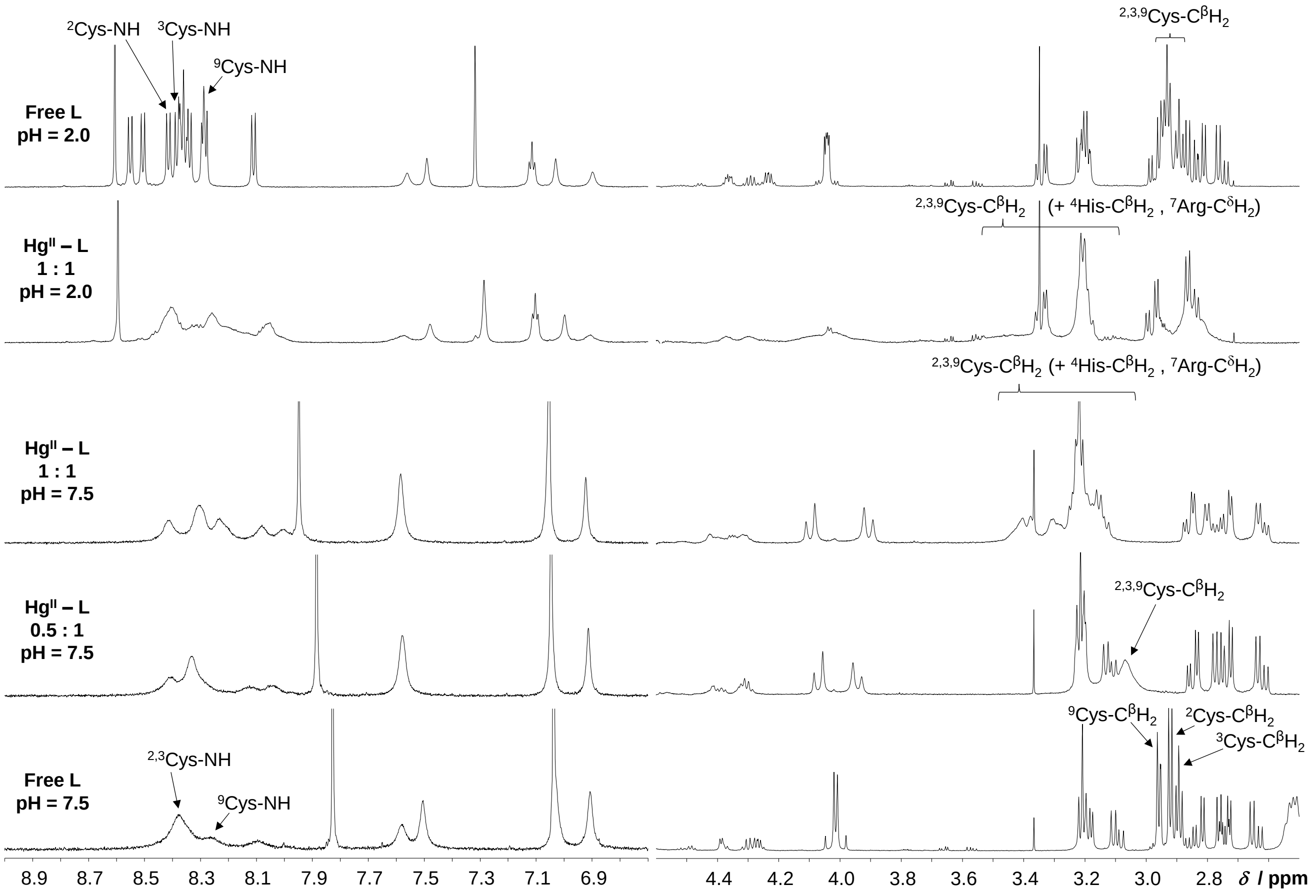

Figure S13. Parts of the ^{1}H NMR spectra of **L** in the absence and presence of 0.5 or 1.0 equiv. of Hg^{II}, recorded at pH = 7.5 (three spectra at the bottom) and 2.0 (upper two spectra) and assignment of the Cys residue related resonances, where it was possible (T = 298 K, c(**L**) = 1.0 mM, H_2O/D_2O 90/10 V/V %). The free ligand sample at pH = 7.5 contained the reducing agent TCEP to maintain the reduced (thiol) form of the peptide (c(TCEP) = 1.0 mM). The sharp peak between 3.3 – 3.4 ppm is a resonance of a small amount of methanol. Note that the figure presents experiments performed at 298 K (instead of 282 K) since, in contrast to measurements of As^{III}-**L**, sharper signals were observed at room temperature.

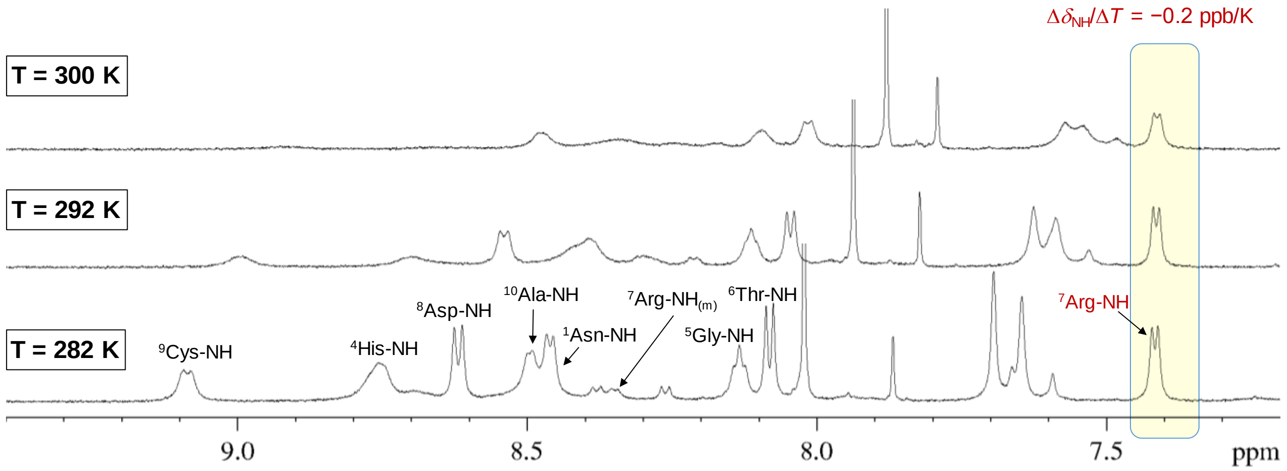

Figure S14. Temperature dependence of the amide ^{1}H NMR resonances in the As^{III}-coordinated peptide at pH = 7.5. While the ^{1}H NMR resonance of the ^{7}Arg-NH signal is nearly unaltered upon changing the temperature, other amide protons, including the ^{7}Arg-NH signal of a minor isomer, are notably shifted. Temperature coefficients for amide protons ($\Delta\delta_{NH}/\Delta T$) correlate well with the presence of H-bonds: slowly exchanging amide protons with a temperature coefficient > −4.5 ppb/K are considered to be in H-bonds.[42,43] Thus, the −0.2 ppb/K NMR temperature coefficient value observed for ^{7}Arg-NH supports the presence of the backbone hydrogen bond between ^{2}Cys-CO and ^{7}Arg-NH.

^{199m}Hg PAC data

Table S8. Parameters fitted to the recorded ^{199m}Hg PAC data with their errors in parentheses (last digit) (see the main text, Figure 7 and the Experimental Procedures for details). The relative amplitudes may not be fully reliable due to the large difference in frequency spread, $\Delta\nu_Q$, for the two nuclear quadrupole interactions (NQIs). NQIs with large frequency spread tend to pick up too much amplitude.

pH	ν_Q (GHz)	η	$\Delta\nu_Q$ (GHz)	λ (μs^{-1})	$A \times 100$	χ_r^2
4.4	1.46(1)	0.17(2)	0.07(1)	86(15)	16.2(8)	0.73
6.2	1.46 [a]<br>1.02 [a]	0.17 [a]<br>0.68 [a]	0.07 [a]<br>0.006 [a]	124(14)<br>124(14)	13(1)<br>3.8(8)	0.86
~7	1.46 [a]<br>1.02 [a]	0.17 [a]<br>0.68 [a]	0.07 [a]<br>0.006 [a]	166(15)<br>166(15)	12(2)<br>6.2(9)	0.89
8.0	1.02(2)	0.68(3)	0.006(3)	91(40)	11(1)	0.81
Reference compound [b]	1.451(9)	0.06(7)	0.00(1)	39(22)	15(1)	0.63

[a] Fixed in the fit. [b] $Hg(Cys)_2$ (solid), see Ref. 11.

EXAFS data

EXAFS spectra were recorded for the As^{III}–**L** (As K-edge) complex and the Hg^{II}–**L** (Hg L_{III}-edge) complex, as well as for two reference samples using glutathione (GSH) as ligand, aimed to represent coordination by three thiolates for As^{III} and Hg^{II}, respectively (see Figure 8 in the main text, Table S9, and Figure S15).

The composition of the As^{III}–GSH sample was planned according to the conditions in the preparation of the same reference for previous EXAFS studies.[44] In addition, we also simulated species distribution profiles using the conditional stability constants available for the As^{III}–GSH system at 37 °C.[38] These data indicated that the tris-complex $As(GSH)_3$ should be highly dominant in a solution containing ~10 mM arsenous acid and GSH in a 15-fold excess over As^{III}.

A similar strategy was used in the preparation of the reference sample Hg^{II}–GSH. pH-dependent species distribution profiles were simulated by using literature stability constant values for a series of Hg^{II}–GSH complexes of different compositions and protonation states.[14,45] These profiles indicated that tris-thiolate coordinated $Hg(GSH)_3$ complexes are dominant at a significant GSH excess (12-fold) over Hg^{II} in the pH-range of 8.0–8.5 (see the detailed discussion and Figure S16 below).

Fitting of data recorded for the As^{III}–GSH reference using a model with only sulfur atoms in the first coordination sphere gives a coordination number of 2.9(1) and As-S bond lengths of 2.257(7) Å (Table S9) in a good correlation with data published previously for the same system.[44,46]

Fitting the EXAFS data for the Hg^{II}–GSH reference with only sulfur atoms in the first coordination sphere gives the expected coordination number of 3.0(1) and a Hg–S bond length of 2.53(1) Å. The bond length, however, is slightly longer than typical Hg-S bond lengths displaying HgS_3 coordination, as determined by x-ray diffraction (2.40 to 2.51 Å, with an average of 2.44 Å),[47] as well as by EXAFS data on Hg-GSH complexes.[14] Results of detailed speciation studies that characterize the composition and thermodynamic stability of the Hg^{II}–GSH complexes formed at different conditions (pH and Hg^{II}:GSH concentration ratio),[45] as well as spectroscopic studies,[14] indicate that di- and tris-thiolate coordinated species are in an equilibrium from pH ~ 6 to 10 and that this equilibrium is substantially shifted towards HgS_3 around pH 8 and with a large excess GSH. The stability data sets published for the Hg^{II}–GSH system[14,45] *vide supra* let us calculate a distribution of Hg^{II} between complexes of HgS_2 and HgS_3 coordination environment and reflected that in our reference sample over 90 % of Hg^{II} is present in the latter form with only a small fraction of HgS_2. Our simulated speciations also showed that 4 glutathione-coordinated species form only above pH 9-10 under a decent GSH excess, as observed experimentally by EXAFS spectra recorded at room temperature.[48] However, a comparison of data measured for alkaline (pH ~ 10.5–11) samples at ambient temperature and in frozen glycerol/water (33-67 % *V*/*V*) glass indicated a significant shift of speciation towards complexes with higher coordination numbers, i.e. four-coordinate HgS_4 species, along with the decreased temperature.[48] Nevertheless, it has not been documented how reduced temperature affects the speciation at pH 8.0, where the complexed and unbound GSH is present in a differently protonated state, as compared to alkaline conditions. We attempted fitting the data, collected for the frozen Hg^{II}–GSH reference sample, by fixing the coordination number to 4, but the quality of the fit was significantly worse than using coordination number 3. However, introducing a combination of 3- and 4-coordinate species resulted in a remarkably better fit with bond length values that correlate well with literature data on HgS_3 and HgS_4 structures,[14,47,48] nevertheless, with a large uncertainty on the shorter distance. It is thus presumable that similarly to alkaline pH, the Hg^{II}–GSH speciation equilibrium is shifted towards the HgS_4 species along with the freezing under our conditions (c(GSH) : $c(Hg^{II})$ = 252 mM : 21.0 mM, pH 8.2).

Table S9. EXAFS fits for the As^{III}–GSH and Hg^{II}–GSH reference, as well as for the As^{III}–**L** and Hg^{II}–**L** samples using models with single As–S and Hg–S bond length values, as well as models introducing a mixture of species and/or non-identical bond length values for a species

Reference samples — Models with single As–S and Hg–S bond length values

		N	%	*R* / Å	DW / $Å^2$	ΔE_0 / eV	*R* factor
As^{III}–GSH reference	As–S	2.9(1)		2.257(7)	0.002(1)	9(1)	0.019
Hg^{II}–GSH reference	Hg–S	3.0(1)		2.53(1)	0.005(1)	8(1)	0.019
Hg^{II}–GSH reference	Hg–S	3 *		2.53(1)	0.004(1)	8(1)	0.019
Hg^{II}–GSH reference	Hg–S	4 *		2.53(1)	0.007(1)	8(2)	0.040

Models using two different coordination numbers and/or two different bond length values

		N	%	*R* / Å	DW / $Å^2$	ΔE_0 / eV	*R* factor
Hg^{II}–GSH reference	Hg–S<br>Hg–S	3 *<br>4 *	18 ± 13<br>82 ± 9	2.40 (10)<br>2.53(2)	0.003(2)	7(2)	0.013

Peptide samples — Models with single As–S and Hg–S bond length values

Sample		N	%	*R* / Å	DW / $Å^2$	ΔE_0 / eV	*R* factor
As^{III}–peptide	As–S	2.9(1)		2.262(6)	0.002(1)	9(1)	0.014
Hg^{II}–peptide	Hg–S	2.5(3)		2.46 (1)	0.008(2)	5(2)	0.019

Models using two different coordination numbers and/or two different bond length values

Sample		N	%	*R* / Å	DW / $Å^2$	ΔE_0 / eV	*R* factor
As^{III}–peptide	As–S<br>As–S	2 *<br>1 *		2.27(4)<br>2.24(5)	0.002(2)	9(2)	0.015
Hg^{II}–peptide	Hg–S<br>Hg–S	3 *<br>2 *	51 ± 9<br>49 ± 7	2.50 (4)<br>2.36(5)	0.003(2)	4(1)	0.016
Hg^{II}–peptide	Hg–S<br>Hg–S<br>Hg–S	2<br>1<br>2	69 ± 9<br><br>31 ± 9	2.50(5)<br>2.38(3)<br>2.33(8)	0.003 *	4(2)	0.015

Parameters marked with * were not refined in the specific fit to keep the number of parameters to a minimum. The highlighted models are presented in Figure 8 and S15.

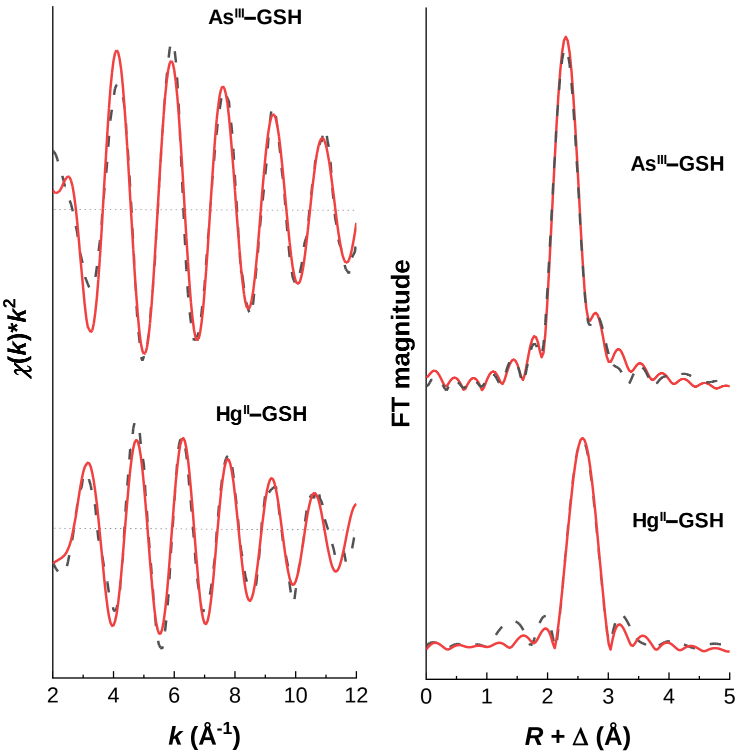

Figure S15. EXAFS spectra and corresponding Fourier transforms of the As^{III}-GSH (top), and Hg^{II}-GSH (bottom) reference systems as dashed lines with the simulated models of a single scattering path (As-S or Hg-S) shown in red solid lines. The Hg^{II}–GSH data was fitted with a model including a mixture of a 3- and 4-coordinate species (see Table S9). (c(As^{III}) : c(GSH) = 1 : 15, c(As^{III}) = 10.2 mM, pH 7.6; c(Hg^{II}) : c(GSH) = 1 : 12, c(Hg^{II}) = 21.0 mM, pH 8.2)

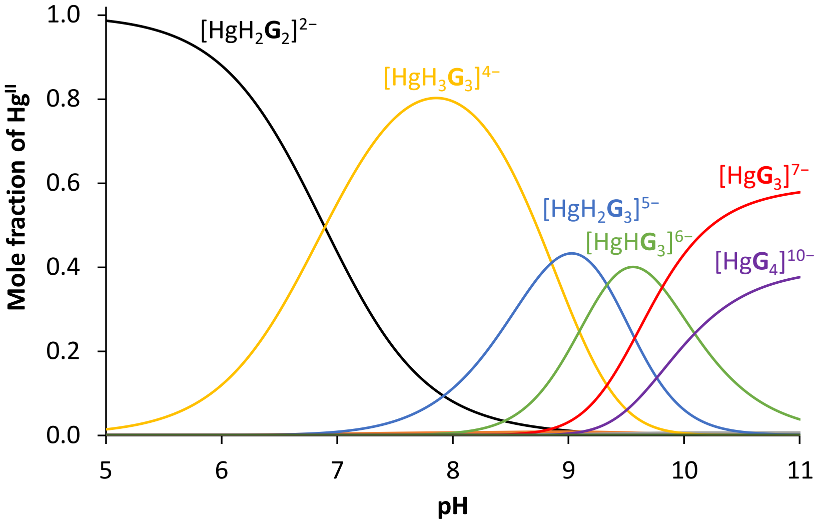

Figure S16. Species distribution curves calculated for Hg^{II}:GSH for the conditions used in the reference sample, based on the stability data from Ref. 45 and 14 (the latter describes slightly refined constants for the tris-complexes). **G** represents the non-protonated GSH molecule with a −3 charge. The differently protonated tris-complexes ($[HgH_3G_3]^{4-}$, $HgH_2G_3]^{5-}$, $HgHG_3]^{6-}$) clearly dominate between pH ~ 8.0–8.5 (at ambient temperature!). (T = 25 °C, c(Hg^{II}) = 21.0 mM, c(GSH) = 252 mM)

Computational studies

Table S10. S–metal ion–S angles for the DFT-optimized small models presented in Figure 9 in the main text.

	A	B	C	D
^{2}Cys–M–^{3}Cys	105.3	127	107.1	111.1
^{3}Cys–M–^{9}Cys	97.6	118.5	97.5	103.0
^{9}Cys–M–^{2}Cys	90.8	113.9	91.6	97.9

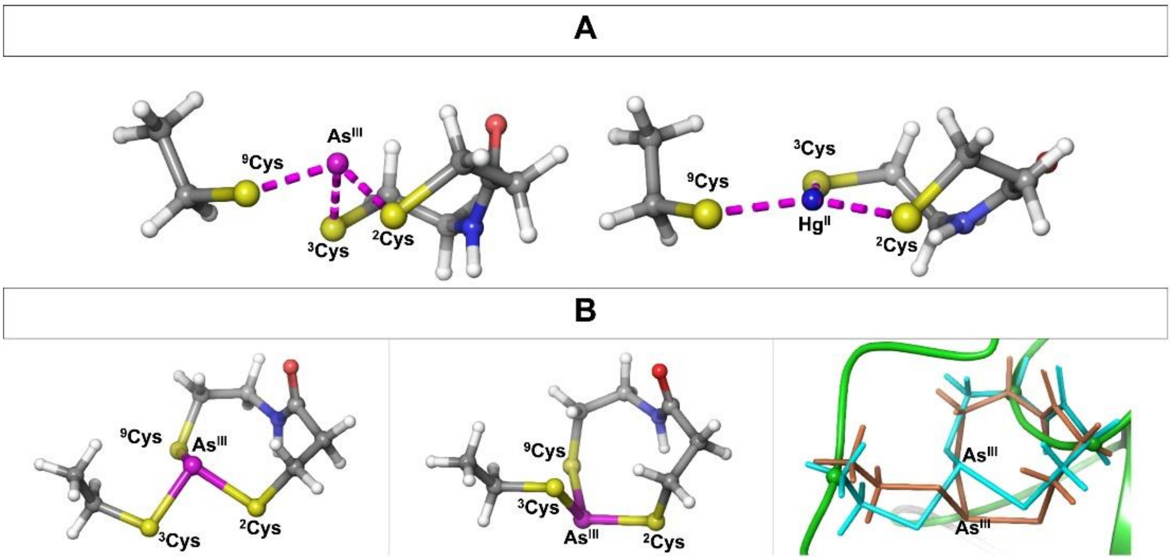

Figure S17. DFT-optimized small model structures
A) Left: Crystal structure cutout of the As^{III}-bound AfArsR (saturated with H) (pdb: 6J05)[20]; Right: DFT optimized structure of the Hg^{II} bound small model highlighting the tilted amide group, as compared to the orientation of the amide group between ^{2}Cys and ^{3}Cys residues in the crystal structure.
B) DFT structure with As^{III} bound in an *all-endo* conformation (left) compared to the structure with As^{III} bound in an *all-exo* conformation (middle); The right panel compares the two structures with an overlay of the skeleton of the protein backbone with highlighted C$^{\alpha}$ atoms of the Cys residues. The *exo*-conformation (brown) is more displaced from the C$^{\alpha}$ positions of the crystal structure as compared to the *endo*-conformation (cyan).

The DFT optimized small models (the ^{2}Cys-^{3}Cys fragment and CH_3-CH_2-S^{-} ion bound to As^{III} or Hg^{II}) and minimal model systems (three CH_3-S^{-} ions bound to As^{III} or Hg^{II}) of the metal sites are shown in Figure S18. The DFT optimizations were started individually from the appropriately prepared crystal structure starting points. Depending on the system, preparation included the addition of hydrogen atoms, substitution of Hg^{II} for As^{III} or manipulation to produce the *exo*-form. Cartesian coordinates for all optimized structures are included below.

The small models are discussed separately in the main article, whereas the following is a brief comparison of these models (Figure S18.A,D,G) with their minimal model counterparts (Figure S18.B,E,H).

Bond lengths between S and the metal ion remain largely consistent when comparing a small model system with its minimal counterpart. However, such comparison of S-metal ion-S angles show more variation. It is evident that the minimal models feature smaller ^{3}Cys-S-metal ion-S-^{2}Cys angles relative to the small models. For instance, this angle decreases from 107.1° to 103.8° when comparing the small As^{III} *endo*-model (Figure S18.A) with the minimal As^{III} *endo*-model (Figure S18.B). It thus appears that the amide plane between ^{3}Cys and ^{2}Cys acts as a spacer, imposing subtle geometric constraints on the coordination geometry of the metal ion.

Notwithstanding this, there is little overall change between the optimized structures of the small and minimal As^{III} *endo*-models (see heavy-atom superimposition in Figure S19.A) This indicates that the two adjacent cysteines allow for organization of part of the metal site where the trigonal pyramidal structure is not significantly distorted by the constraints imposed by the protein backbone.

There is a larger change between the optimized structures of the small and minimal As^{III} *exo*-models (see Figure S18.D-E and Figure S19.B) indicating that the forming metalloid binding site is not optimal to accommodate As^{III} in this conformation. This is in agreement with the *endo*-conformation observed in the crystal structure and the difference in the Gibbs free energies, calculated for the small *endo*- and *exo*-models, the former being more stable, see main text.

The change in the optimized structure of the Hg^{II}-model between the small and minimal model systems is surprisingly small, see Figure S18.G-I and Figure S19.C). This suggests that the metalloid site of AfArsR may also well accommodate Hg^{II}, albeit with some structural change, i.e. longer Hg–S bond lengths relative to those of As–S, as well as the change in the orientation of the amide group between ^{2}Cys and ^{3}Cys, as evident from comparing Figure S18.G with the corresponding As^{III} models in Figure S18.A and Figure S18.D.

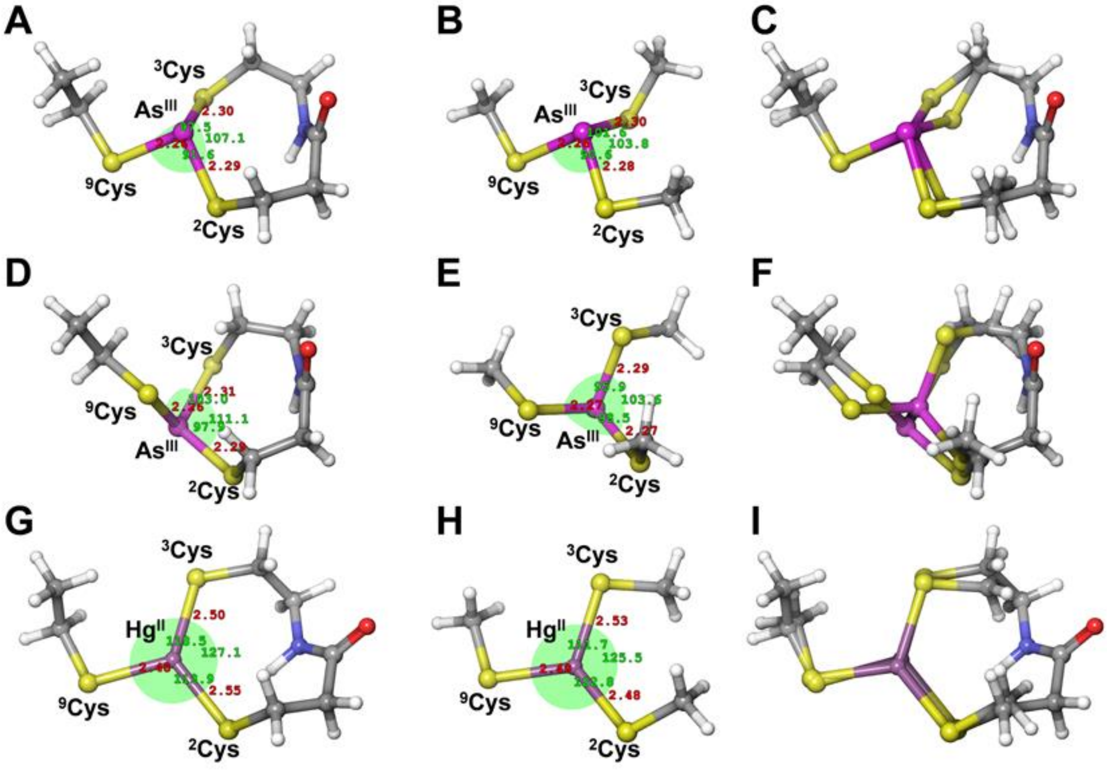

Figure S18. DFT optimized small models and minimal models of the metal binding site. **A, D, G**: Small models corresponding to Figures 9.C (As^{III} in *endo*-conformation), 9.D (As^{III} in *exo*-conformation) and 9.B (Hg^{II} model) in the main text. **B, E, H**: Minimal models. **C, F, I**: Overlay (without superimposition) of small and minimal models.

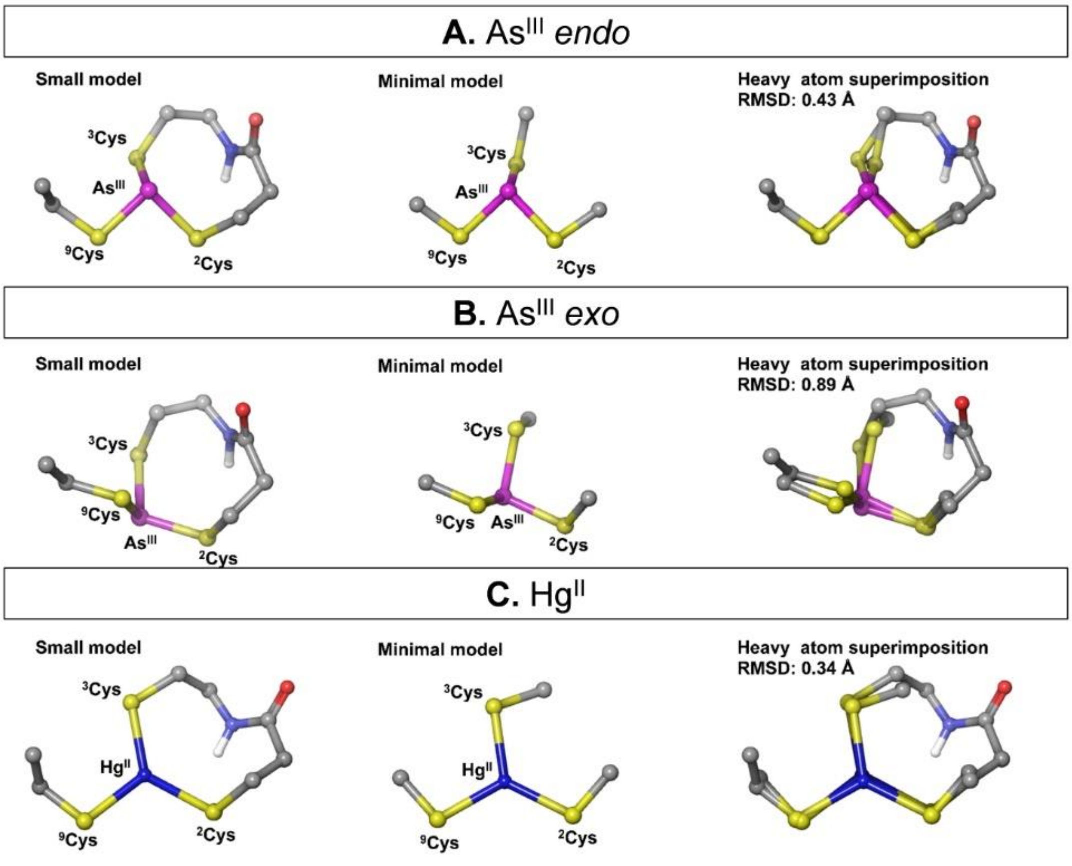

Figure S19. Superimposition of small and minimal models (only heavy atoms and polar hydrogens shown). Superimposition and RMSD based on all heavy atoms common to the minimal and small model.

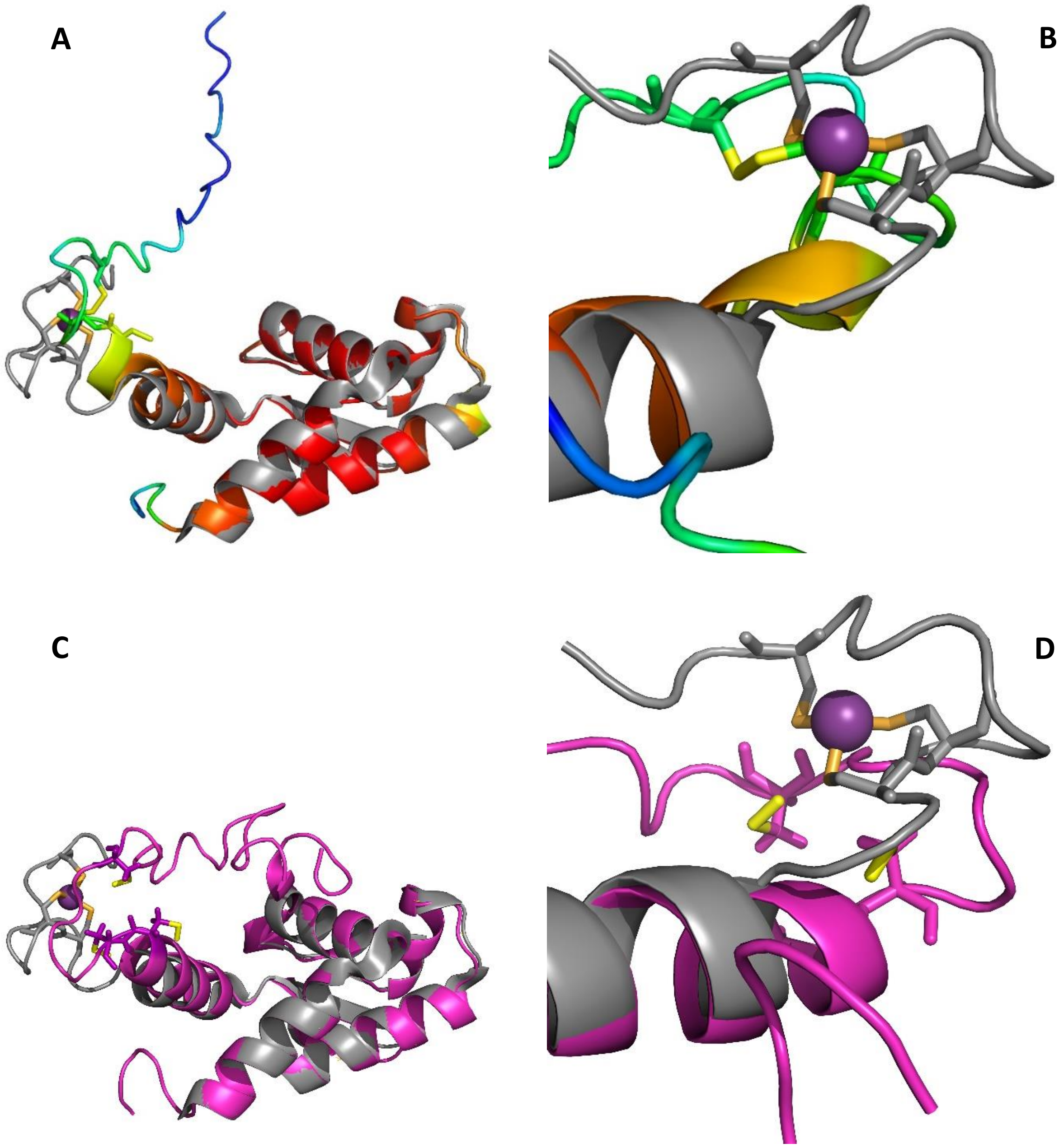

Figure S20. Superimposition of the X-ray crystal structure of AfArsR-As with the structure of apo AfArsR predicted by Alphafold (**A, B**) and I-TASSER (**C, D**)

A) and **B**): The X-ray crystal structure of AfArsR-As (one subunit, color codes: protein scaffold: grey, cysteine sulfur atoms: orange, As: purple) overlaid with the AlphaFold[49,50] structure of the apo AfArsR monomer colored according to the reliability scores (B-factor) of the predicted structure, displaying lower scores in green-blue and higher scores in orange-red. (Cysteine sulfurs are highlighted as sticks in yellow.)

C) and **D**): The X-ray crystal structure of AfArsR-As (one subunit, color codes: protein scaffold: grey, cysteine sulfur atoms: orange, As: purple) overlaid with the I-TASSER[51–53] structure of the apo AfArsR monomer colored in magenta. (Cysteine sulfurs are highlighted as sticks in yellow.)

The images were prepared by PyMOL[54] depicting the overall structures on the left images (**A** and **C**) and a zoom at the metal site on the right images (**B** and **D**). Note that AlphaFold predicted a disulfide bridge in the As^{III}-free state of the protein between ^{95}Cys and ^{96}Cys which is most probably not present under reducing (cellular) conditions. The three cysteine residues are in their reduced form in the I-TASSER prediction. Apart from this issue, both the Alphafold and I-TASSER predicted structures of the apo protein show an excellent structural match with that of the holo form except the C-terminal part, including the metalloid binding site, which notably differs from the crystal structure. The low B-factors for these residues, provided by Alphafold, indicate that this region of the protein is flexible and partially unstructured.

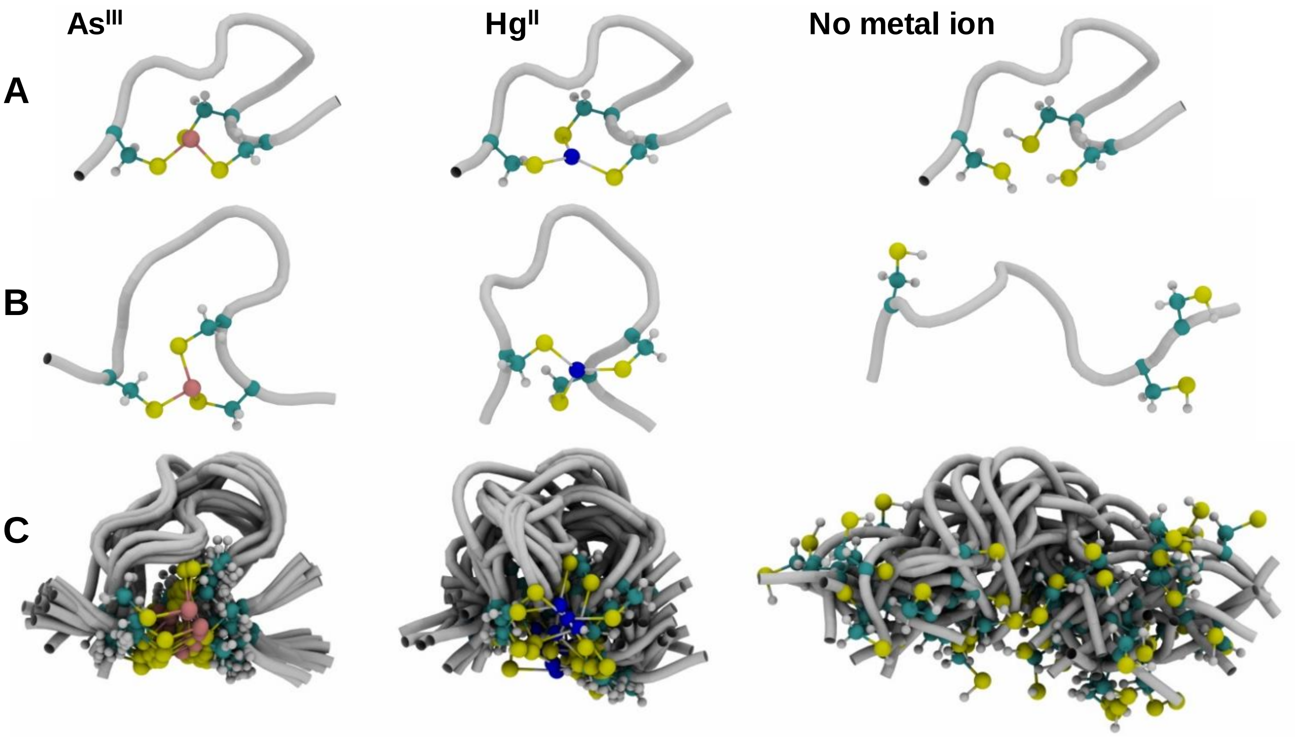

Figure S21. MD snapshots from the trajectories for the three simulations on the Ac-NCCHGTRDCA-NH_2 peptide with As^{III} (left), Hg^{II} (middle) and without metal ions (right). A: Initial structures. B: Simulation endpoints at 2000 ns. C: Superposition of all frames in each trajectory reduced to 20 frames for clarity.

Coordinates of optimized DFT structures

<u>As^{III} *endo*, small model</u>

27

Coordinates from ORCA-job 6J05_small_AsIII_inp

C	-11.98372715847220	6.99079139993306	-2.82883179275886
C	-13.07673073765957	7.05243485445071	-3.89340030303660
O	-12.88800879965514	7.56075610463006	-4.99933204598786
C	-11.95546918370931	8.24284260670942	-1.93804880851114
S	-13.34675823641478	8.36179006938673	-0.73146691092852
N	-14.27918160759167	6.51316018921946	-3.51845844558080
C	-15.47240838046116	6.66022329523330	-4.33750629534185
C	-16.14480033579422	8.03294781719091	-4.20167682794721
S	-16.60117964084375	8.44083117446289	-2.46662436677049
C	-16.54245350253141	12.87651530192042	-1.44519992963889
C	-16.97882042115967	11.63467846400720	-0.67680411683011
S	-15.59336127230024	10.61540388943081	-0.01295166546480
As	-14.76644783389524	9.70893146787521	-1.91557246772625
H	-12.08811025016331	6.09333009266504	-2.20042704374628
H	-11.92429519527158	9.15658994624028	-2.54910848035138
H	-11.05142448161256	8.23699702200158	-1.31295089831896
H	-14.41317103961047	6.31704182924689	-2.52821928305341
H	-16.17638648829296	5.86332726033665	-4.05940204469198
H	-17.07752465923857	8.05729742298469	-4.78413044389503
H	-15.48350229779624	8.81789430805832	-4.59741941619232
H	-17.42300418169275	13.46578347738455	-1.74596167307386
H	-17.62482554363240	10.98346269779608	-1.28188269485371
H	-17.54198054761606	11.90553413761041	0.22901645409504
H	-11.02637920594471	6.92370657483856	-3.36649919209327
H	-15.17663290704276	6.51374826096825	-5.38703711348172
H	-15.99213987665780	12.60929459397074	-2.35885384762744
H	-15.89127621493920	13.51498574144760	-0.83295034619225

As^{III} endo, minimal model

16

Coordinates from ORCA-job ASIII_start_minimal_RERUN

C	-12.07790196498786	8.12949767087871	-2.01029385424706
S	-12.81406568822480	9.22090223396794	-0.74363808089250
C	-15.81662053203623	8.12569864332140	-4.21176257301036
S	-15.78042955145711	8.00802090946470	-2.38020499049313
C	-17.00308800986651	11.57576709316603	-0.73675992992191
S	-15.62257282928570	10.62158461450384	-0.00602585750946
As	-14.62584590028741	9.94057006316147	-1.91776819232529
H	-11.72747161718032	8.69569727157876	-2.88268568939685
H	-11.21826065606992	7.64512276002246	-1.52952301696616
H	-16.82180867132425	7.84213606974286	-4.54776387024236
H	-15.61607549109672	9.15523211211902	-4.53995236076918
H	-17.66128535326834	10.92145649469369	-1.32200673924199
H	-17.56904284873628	11.98138849469314	0.11189962185387
H	-15.07678287731432	7.45144478883799	-4.65950915671872
H	-12.80088059356365	7.36065436760260	-2.31398151871626
H	-16.64026741530021	12.40752641224517	-1.35282379140258

As^{III} *exo*, small model

27

Coordinates from ORCA-job 6J05_small_inv_ASIII_inp

C	-12.04261205224706	7.30004446309951	-2.75791568412450
C	-13.11763772899587	7.41388220620328	-3.83392638794959
O	-12.97607217005578	8.12662354603930	-4.82720779483037
C	-12.36659274913716	8.15031437554178	-1.51906106744999
S	-13.58765178846275	7.38775597214674	-0.36004381079536
N	-14.25426869961430	6.68637876082764	-3.58782779838913
C	-15.47776186112272	6.95071958629536	-4.32627776084854
C	-16.31664846326899	8.05888561231636	-3.68480222474826
S	-16.98322502020504	7.60910691219685	-2.03322634268464
C	-16.37441371853795	12.69278122207691	-1.83822991847410
C	-16.56095905922064	11.31378207885317	-1.21123538843992
S	-14.94065165151891	10.42554216303239	-1.21610089446066

H	-11.88005516617430	6.25214827013329	-2.46163196705051
H	-12.71094789622382	9.15341662548732	-1.80587430845481
H	-11.46689374545878	8.27657775118415	-0.90080390034193
H	-14.35244008823400	6.28664077331679	-2.65315352579556
H	-16.06049717230873	6.02154455802328	-4.40578789227772
H	-17.19968778474828	8.27985878388347	-4.30314455739553
H	-15.72739627090384	8.98364788344890	-3.58263050026620
H	-17.33411992851170	13.23067058533186	-1.83624673933992
H	-17.28370905001855	10.71025230170519	-1.77744304469705
H	-16.91044919587619	11.39599621113419	-0.17319270767278
H	-11.11190878892072	7.67691122804161	-3.20551180344749
H	-15.18121548621942	7.26711767454920	-5.33699305970111
H	-16.02749818108106	12.61638523491319	-2.87789194717747
H	-15.64692190236117	13.29515309977553	-1.27664231489388
As	-15.62326438057164	8.44356212044238	-0.36779665829292

<u>As^{III} *exo*, minimal model</u>

16

Coordinates from ORCA-job ASIII_inv_minimal

C	-12.21125099467765	8.97954195992814	-1.93496435619011
S	-13.24132334186792	7.86819674529801	-0.91426783025815
C	-16.07016407626822	7.45437960533684	-3.99583162097805
S	-15.51368860882910	9.09442343050752	-3.39673681366553
C	-16.72793353723094	11.42681411575537	-0.66186830495047
S	-15.01768740935158	10.78880905169257	-0.50829167168453
H	-12.00817851750493	9.92086633141447	-1.41249150304000
H	-11.26969981503693	8.44561847806329	-2.11751616217532
H	-16.28833605609043	7.57545409092072	-5.06485656800564
H	-15.28040346235936	6.70317422551512	-3.87430140004399
H	-17.06308621918648	11.40818525625784	-1.70565185320522
H	-17.42321666806583	10.86319059076558	-0.02716610328677
As	-15.36341135888926	8.63952022577782	-1.16124424381397
H	-16.98397176363835	7.13001863845505	-3.48240761505434
H	-12.71414479574317	9.17319460277671	-2.89247760083907
H	-16.69140337525963	12.46671265153481	-0.31162635280880

Hg^{II}, small model

27

Coordinates from ORCA-job 6J05_small_inp

C	-11.84474114364240	6.69855780375118	-2.96198956679808
C	-13.01286090727148	6.49844411186949	-3.92875666514518
O	-12.83346027314384	6.20182730241497	-5.11838347115188
C	-11.85099705532013	8.05011807059991	-2.22099470888415
S	-12.97459437281188	8.13983533337593	-0.75516258885303
N	-14.24390960136066	6.67949689378337	-3.37246994972443
C	-15.44372958704434	6.80845970895419	-4.18251868265764
C	-15.72625082579696	8.25037568916801	-4.62808799715070
S	-16.43504607925259	9.39309406116722	-3.37045372822144
C	-16.99468592143160	12.86814585561636	-1.33003926902605
C	-17.22823595158820	11.88383305835776	-0.18648538033267
S	-15.69339476787544	11.11079052012680	0.47919861336269
Hg	-14.98795224234570	9.58164852101865	-1.34294368234101
H	-11.83145352252907	5.88408938665193	-2.21845843352590
H	-12.08129202885630	8.86582392755817	-2.92060499795820
H	-10.83979262164292	8.23982109977747	-1.83021998029104
H	-14.25265003832855	7.03455202608131	-2.40522323779524
H	-16.30315663150451	6.42122264069266	-3.61394941054698
H	-16.48764911801603	8.22385678660899	-5.42542452339841
H	-14.81150354258308	8.67689973091159	-5.06433770838417
H	-17.94083262225893	13.34736373704539	-1.63991339790279
H	-17.92914781799589	11.09582608774648	-0.49740393933951
H	-17.68422225530896	12.40240705949456	0.67342105361877
H	-10.93469802563464	6.60906617933091	-3.57490363370534
H	-15.29218546264507	6.17852922163105	-5.07322835983834
H	-16.57382117898206	12.35139665846658	-2.20514567218457
H	-16.28773640482792	13.65481852779853	-1.02722068182459

Hg^{II}, minimal model

16

Coordinates from ORCA-job HG_start_minimal

C	-12.04348466913751	7.96606190608095	-2.41839970447227
S	-12.57818256220735	8.59146080880477	-0.77578180536208
C	-15.46718826573326	8.03565881100052	-4.15653772244539
S	-16.09507721260899	9.55320635082676	-3.33277115057641
C	-17.34258379287671	11.74660491022738	-0.44961736815792
S	-15.83623913653995	11.22235730085561	0.46329629798002
Hg	-14.73791644649818	9.73390119495992	-1.20677656817256
H	-12.02232752911247	8.76675744832267	-3.16968047840791
H	-11.02281573107436	7.56873514993624	-2.30706383610235
H	-16.11226714371098	7.82998838268317	-5.02485601208235
H	-14.43551577181964	8.15944963169804	-4.51377197514242
H	-17.86598613800946	10.88599948142631	-0.88682667989814
H	-18.01587549265457	12.24823066236909	0.26246551136270
H	-12.69290521618083	7.15796995655180	-2.78175975870820
H	-15.50485423485045	7.16484763076401	-3.48790349256815
H	-17.09918065698495	12.45147037349255	-1.25681525724655

References

(1) Ellman, G. L. Tissue sulfhydryl groups. *Arch. Biochem. Biophys.* **1959**, *82*, 70–77.

(2) Riddles, P.W.; Blakeley, R. L.; Zerner, B. Reassessment of Ellman's reagent. *Methods Enzymol.* **1983**, *91*, 49–60.

(3) Zékány, L.; Nagypál, I.; Peintler, G. *PSEQUAD for chemical equilibria*, Technical Software Distributors, Baltimore, MD, **1991**.

(4) Sillen, L. G. Electrometric investigation of equilibria between mercury and halogen ions. VIII. Survey and conclusions. *Acta Chem. Scand.* **1949**, *3*, 539–553.

(5) Grenthe, I.; Plyasunov A. V.; Spahiu, K. In *Modelling in Aquatic Chemistry*. Grenthe, I.; Puigdomenech, I., Eds. OECD Publications, **1997**, Ch. IX, pp 325–426.

(6) Powell, K. J.; Brown, P. L.; Byrne, R. H.; Gajda, T.; Hefter, G.; Sjöberg, S.; Wanner, H. Chemical speciation of environmentally significant heavy metals with inorganic ligands. Part 1: The Hg^{2+}– Cl^{-}, OH^{-}, CO_3^{2-}, SO_4^{2-}, and PO_4^{3-} aqueous systems. (IUPAC Technical Report) *Pure Appl. Chem.* **2005**, *77*, 739–800.

(7) Mesterházy, E.; Lebrun, C.; Crouzy, S.; Jancsó, A.; Delangle, P. Short oligopeptides with three cysteine residues as models of sulphur-rich Cu(I)- and Hg(II)-binding sites in proteins. *Metallomics* **2018**, *10*, 1232–1244.

(8) Beynon, R.; Easterby, J. *Buffer Solutions*, Oxford University Press, New York, **1996**.

(9) Catherall, R.; Andreazza, W.; Breitenfeldt, M.; Dorsival, A.; Focker, G. J.; Gharsa, T. P.; Giles, T. J.; Grenard, J.-L.; Locci, F.; Martins, P.; Marzari, S.; Schipper, J.; Shornikov, A.; Stora, T. The ISOLDE facility. *J. Phys. G: Nucl. Part. Phys.* **2017**, *44*, 094002.

(10) Jäger, M.; Iwig, K.; Butz, T. A compact digital time differential perturbed angular correlation-spectrometer using field programmable gate arrays and various timestamp algorithms. *Rev. Sci. Instrum.* **2011**, *82*, 065105.

(11) Jancsó, A.; Correia, J. G.; Balogh, R. K.; Schell, J.; Jensen, M. L.; Szunyogh, D.; Thulstrup, P. W.; Hemmingsen, L. A reference compound for ^{199m}Hg perturbed angular correlation of γ-rays spectroscopy. *Nucl. Instrum. Methods Phys. Res. A* **2021**, *1002*, 165154.

(12) Frauenfelder, H.; Steffen, R. M. In *Alpha-, Beta and Gamma-Ray Spectroscopy*; North Holland, Amsterdam, **1965**, p. 997.

(13) Hemmingsen, L.; Sas, K. N.; Danielsen, E. Biological Applications of Perturbed Angular Correlations of γ-Ray Spectroscopy. *Chem. Rev.* **2004**, *104*, 4027–4062.

(14) Mah, V.; Jalilehvand, F. Glutathione Complex Formation with Mercury(II) in Aqueous Solution at Physiological pH. *Chem. Res. Toxicol.* **2010,** *23,* 1815–1823.

(15) Ravel, B.; Newville, M. ATHENA, ARTEMIS, HEPHAESTUS: data analysis for X-ray absorption spectroscopy using IFEFFIT. *J. Synchrotron Rad.* **2005**, *12*, 537–541.

(16) Jancsó, A.; Szunyogh, D.; Larsen, F. H.; Thulstrup, P. W.; Christensen, N. J.; Gyurcsik, B.; Hemmingsen, L. Towards the role of metal ions in the structural variability of proteins: Cd^{II} speciation of a metal ion binding loop motif. *Metallomics* **2011**, *3*, 1331–1339.

(17) Jancsó, A.; Gyurcsik, B.; Mesterházy. E.; Berkecz, R. Competition of zinc(II) with cadmium(II) or mercury(II) in binding to a 12-mer peptide. *J. Inorg. Biochem.* **2013**, *126*, 96–103.

(18) Gans, P.; Sabatini, A.; Vacca, A. Investigation of equilibria in solution. Determination of equilibrium constants with the HYPERQUAD suite of programs. *Talanta* **1996**, *43*, 1739–1753.

(19) Szekeres, L. I.; Bálint, S.; Galbács, G.; Kálomista, I.; Kiss, T.; Larsen, F. H.; Hemmingsen, L.; Jancsó, A. Hg^{2+} and Cd^{2+} binding of a bioinspired hexapeptide with two cysteine units constructed as a minimalistic metal ion sensing fluorescent probe. *Dalton Trans.* **2019**, *48*, 8327–8339.

(20) Prabaharan, C.; Kandavelu, P.; Packianathan, C.; Rosen, B. P.; Thiyagarajan, S. Structures of two ArsR As(III)-responsive transcriptional repressors: Implications for the mechanism of derepression. *J. Struct. Biol.* **2019**, *207*, 209–217.

(21) Berman, H. M.; Westbrook, J.; Feng, Z.; Gilliland, G.; Bhat, T. N.; Weissig, H.; Shindyalov, I. N.; Bourne, P. E. The Protein Data Bank. *Nucleic Acids Res.* **2000**, *28*, 235–242.

(22) Schrödinger Release 2018-2: Maestro, *Schrödinger,* LLC, New York, NY, **2018**.

(23) Becke, A. D. Density-functional exchange-energy approximation with correct asymptotic behavior. *Phys. Rev. A Gen. Phys.* **1988**, *38*, 3098–3100.

(24) van Lenthe, E.; Snijders, J. G.; Baerends, E. J. The zero‐order regular approximation for relativistic effects: The effect of spin–orbit coupling in closed shell molecules. *J. Chem. Phys.* **1996**, *105*, 6505–6516.

(25) Weigend, F.; Ahlrichs, R. Balanced basis sets of split valence, triple zeta valence and quadruple zeta valence quality for H to Rn: Design and assessment of accuracy. *Phys. Chem. Chem. Phys.* **2005**, *7*, 3297–3305.

(26) Pantazis, D. A.; Chen, X.-Y.; Landis, C. R.; Neese, F. All-Electron Scalar Relativistic Basis Sets for Third-Row Transition Metal Atoms. *J. Chem. Theory Comput.* **2008**, *4*, 908–919.

(27) Weigend, F. Accurate Coulomb-fitting basis sets for H to Rn. *Phys. Chem. Chem. Phys.* **2006**, *8*, 1057–1065.

(28) Neese, F. Software update: The ORCA program system—Version 5.0. *WIREs Computational Molecular Science* **2022**, *12*, e1606.

(29) Schrödinger Release 2018-1: Desmond Molecular Dynamics System, *D. E. Shaw Research,* New York, NY, **2018**. Maestro-Desmond Interoperability Tools, *Schrödinger,* New York, NY, **2018**.

(30) Schrödinger Release 2018-1: MacroModel, *Schrödinger,* LLC, New York, NY, **2018.**

(31) Harder, E.; Damm, W.; Maple, J.; Wu, C.; Reboul, M.; Xiang, J. Y.; Wang, L.; Lupyan, D.; Dahlgren, M. K.; Knight, J. L.; Kaus, J. W.; Cerutti, D. S.; Krilov, G.; Jorgensen, W. L.; Abel, R.; Friesner, R. A. OPLS3: A Force Field Providing Broad Coverage of Drug-like Small Molecules and Proteins. *J. Chem. Theory Comput.* **2016,** *12*, 281–296.

(32) Jorgensen, W. L.; Chandrasekhar, J.; Madura, J. D.; Impey, R. W.; Klein, M. L. Comparison of simple potential functions for simulating liquid water. *J. Chem. Phys.* **1983,** *79*, 926–935.

(33) Humphrey, W.; Dalke, A.; Schulten, K. VMD: Visual molecular dynamics. *J. Mol. Graphics* **1996,** *14*, 33–38.

(34) Szekeres, L. I.; Maldivi, P.; Lebrun, C.; Gateau, C.; Mesterhazy, E.; Delangle, P.; Jancso, A. Tristhiolato Pseudopeptides Bind Arsenic(III) in an AsS3 Coordina-tion Environment Imitating Metalloid Binding Sites in Proteins. *Inorg. Chem.* **2023**, *62*, 6817–6824.

(35) Kolozsi, A.; Lakatos, A.; Galbacs, G.; Madsen, A. O.; Larsen, E.; Gyurcsik, B. A pH-Metric, UV, NMR, and X-ray Crystallographic Study on Arsenous Acid Reacting with Dithioerythritol. *Inorg. Chem.* **2008**, *47*, 3832–3840.

(36) Szekeres, L. I.; Gyurcsik, B.; Kiss, T.; Kele, Z.; Jancso, A. Interaction of Arsenous Acid with the Dithiol-Type Chelator British Anti-Lewisite (BAL): Structure and Stability of Species Formed in an Unexpectedly Complex System. *Inorg. Chem.* **2018**, *57*, 7191–7200.

(37) Zahler, W. L.; Cleland, W. W. A Specific and Sensitive Assay for Disulfides. *J. Biol. Chem.* **1968**, *243*, 716–719.

(38) Spuches, A. M.; Kruszyna, H. G.; Rich, A. M.; Wilcox, D. E. Thermodynamics of the As(III)-Thiol Interaction: Arsenite and Monomethylarsenite Complexes with Glutathione, Dihydrolipoic Acid and Other Thiol Ligands *Inorg. Chem.* **2005**, *44*, 2964–2972.

(39) Kitchin, K. T.; Wallace, K. Arsenite binding to synthetic peptides based on the Zn finger region and the estrogen binding region of the human estrogen receptor-α. *Toxicol. Appl. Pharmacol.* **2005**, *206*, 66–72.

(40) Pires, S.; Habjanič, J.; Sezer, M.; Soares, C. M.; Hemmingsen, L.; Iranzo, O. Design of a Peptidic Turn with High Affinity for Hg^{II}. *Inorg. Chem.* **2012**, *51*, 11339–11348.

(41) Szunyogh, D. PhD thesis, University of Szeged (Hungary), **2016**. (https://doktori.bibl.u-szeged.hu/id/eprint/2874/2/Tezis_ENG_Szunyogh.pdf)

(42) Baxter, N. J.; Williamson, M. P. Temperature dependence of ^{1}H chemical shifts in proteins. *J. Biomol. NMR* **1997**, *9*, 359–369.

(43) Cierpicki, T.; Otlewski, J. Amide proton temperature coefficients as hydrogen bond indicators in proteins. *J. Biomol. NMR* **2001**, *21*, 249–261.

(44) Shi, W.; Dong, J.; Scott, R. A.; Ksenzenko, M. Y.; Rosen, B. P. The Role of Arsenic-Thiol Interactions in Metalloregulation of the ars Operon. *J. Biol. Chem.* **1996**, *271*, 9291–9297.

(45) Shoukry, M. M.; Cheesman, B. V.; Rabenstein, D. L. Polarimetric and nuclear magnetic resonance studies of the complexation of mercury by thiols. *Can. J. Chem*. **1988**, *66*, 3184–3189.

(46) Pickering, I. J.; Prince, R. C.; George, M. J.; Smith, R. D.; George, G. N.; Salt D. E. Reduction and Coordination of Arsenic in Indian Mustard. *Plant Physiol.* **2000**, *122*, 1171–1177.

(47) Manceau, M.; Nagy K. L. Relationships between Hg(II)–S bond distance and Hg(II) coordination in thiolates. *Dalton Trans*. **2008**, 1421-1425.

(48) Mah, V.; Jalilehvand, F. Mercury(II) complex formation with glutathione in alkaline aqueous solution. *J. Biol. Chem.* **2008,** *13,* 541–553.

(49) Jumper, J.; Evans, R.; Pritzel, A.; Green, T.; Figurnov, M.; Ronneberger, O.; Tunyasuvunakool, K.; Bates, R.; Žídek, A.; Potapenko, A.; Bridgland, A.; Meyer, C.; Kohl, S. A. A.; Ballard, A. J.; Cowie, A.; Romera-Paredes, B.; Nikolov, S.; Jain, R.; Adler, J.; Back, T.; Petersen, S.; Reiman, D.; Clancy, E.; Zielinski, M.; Steinegger, M.; Pacholska, M.; Berghammer, T.; Bodenstein, S.; Silver, D.; Vinyals, O.; Senior, A. W.; Kavukcuoglu, K.; Kohli, P.; Hassabis, D. Highly accurate protein structure prediction with AlphaFold. *Nature* **2021**, 596, 583–589.

(50) Varadi, M.; Anyango, S.; Deshpande, M.; Nair, S.; Natassia, C.; Yordanova, G.; Yuan, D.; Stroe, O.; Wood, G.; Laydon, A.; Žídek, A.; Green, T.; Tunyasuvunakool, K.; Petersen, S.; Jumper, J.; Clancy, E.; Green, R.; Vora, A.; Lutfi, M.; Figurnov, M.; Cowie, A.; Hobbs, N.; Kohli, P.; Kleywegt, G.; Birney, E.; Hassabis, D.; Velankar, S. AlphaFold Protein Structure Database: massively expanding the structural coverage of protein-sequence space with high-accuracy models. *Nucleic Acids Res.* **2021**, *50*, D439–D444.

(51) Zheng, W.; Zhang, C.; Li, Y.; Pearce, R.; Bell, E. W.; Zhang, Y. Folding non-homology proteins by coupling deep-learning contact maps with I-TASSER assembly simulations. *Cell Rep. Methods* **2021**, *1*, 100014.

(52) Zhang, C.; Freddolino, P. L.; Zhang, Y. COFACTOR: improved protein function prediction by combining structure, sequence and protein-protein interaction information. *Nucleic Acids Res.* **2017**, *45*, W291-299.

(53) Yang, J.; Zhang, Y. I-TASSER server: new development for protein structure and function predictions, *Nucleic Acids Res.* **2015**, *43*, W174-W181.

(54) Schrödinger, L.; DeLano, W. **2020**. *PyMOL*, Available at: http://www.pymol.org/pymol

Appendix

Prediction of CD spectra by semiempirical methods

SESCA[A1] and PDBMD2CD[A2] are recently developed circular dichroism (CD) spectrum prediction programs using structural information either from a Protein Data Bank (PDB) file or from the results of molecular dynamic (MD) simulations. The basis of the CD prediction by these programs is the secondary structure element (SSE) composition of the peptide/protein structures. This approach reveals excellent agreement of the predicted and experimental CD spectra of proteins. They also provide opportunity to get an impression on the intensity range of the CD spectra for short oligopeptides, without considering additional chiral contributions, such as those arising from the charge transfer transitions in metal complexes. The aim of these calculations was to elucidate, whether it was possible to obtain CD predictions that are similar to the experimental spectra based solely on the peptide backbone coordinates.

While trying both programs for CD prediction, we carried out detailed analysis with SESCA, using a basis set optimized for loop-type structures (DS5-4). The NCCHGTRDCA peptide, forming the metalloid binding loop of the AfArsR protein was extracted from the AfArsR-As crystal structure (PDB ID: 6J05) with fixed coordinates and its CD spectrum was calculated in the absence of As^{III}. Predictions were also carried out using the peptide models obtained from the molecular dynamic simulations with the peptide only, as well as with its As^{III} or Hg^{II} complexes. 20 equidistant snapshots from 100 to 2000 ns were selected from each 2000 ns MD trajectories.

Analyzing these structures by the DISICL[A3] algorithm that uses the ϕ, ψ backbone dihedral angles to classify tetrapeptide segments into 19 SSEs, remarkable differences were observed (Figure A1). The SSE composition of the free peptide showed the largest random variations in time, which is consistent with its flexibility in solution. The peptide structures from the MD simulations of the As^{III}-peptide and Hg^{II}-peptide complexes shoved much less variations in their SSEs, both reflecting high similarity to the SSE distribution calculated for the peptide derived from the crystal structure and included as a reference in Figure A1. However, the Hg^{II} complex quite often exhibited β-strand (NBS) and polyproline helix (PP) SSEs not found in the reference structure.

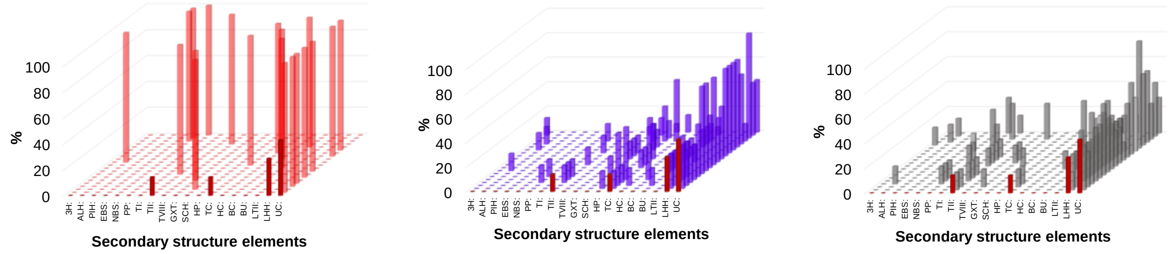

Figure A1. The secondary structure element compositions determined by the DISICL algorithm for the free NCCHGTRDCA peptide (left panel), the As^{III}- (middle panel) and the Hg^{II}-bound (right panel) structures from equidistant MD snapshots between 100 and 2000 ns. The first row in each panel represents the result obtained for the peptide from the AfArsR-As crystal structure (PDB ID: 6J05) for comparison. Definitions of SSEs are as follows: 3/10-helix (3H); α-helix (ALH); π-helix (PIH); Ext. β-strand (EBS); β-strand (NBS); PP-helix (PP); Turn type 1 (TI); Turn type 2 (TII); Turn type 8 (TVIII); γ-turn (GXT); Schellman-turn (SCH); Hairpin 2:2 (HP); Turn-cap (TC); Helix-cap (HC); β-cap (BC); β-bulge (BU); Left turn 2 (LTII); Left-handed helix (LHH); and unclassified (UC) structures.

In accordance with the SSE distributions, the predicted CD spectra related to the from the 20 MD snapshots of the free peptide showed the largest deviations, in agreement with the high flexibility of the molecule. The averaged spectra of the free peptide, the As^{III}-peptide and the Hg^{II}-peptide complexes, together with their standard deviations are shown in Figure A2.

Figure A3. collects the predicted CD spectra for the MD snapshots at 2000 ns, i.e. at the end of the calculation. All the structures collected in the right panel of Figure A3 are different. Based on the MD trajectory of the As^{III}-bound peptide, mainly two types of structures are expected. Therefore, we have repeated the calculation with the 250 ns snapshot taken from the part of the RMSD curve with lower RMSD values. The results shown in

Figure A4. reveal an even better agreement with the experimental spectra in terms of the intensity range, than those in Figure A3, while the backbone structure of the As^{III}-bound form is similar to that of the peptide excised from the AfArsR-As crystal structure.

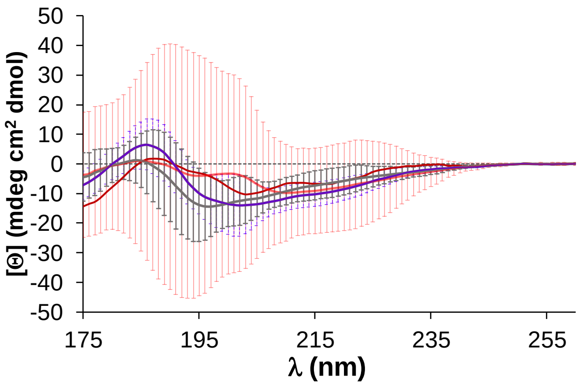

Figure A2. The averages of the predicted CD spectra and their standard deviations for the free NCCHGTRDCA peptide (red), the As^{III}- (violet) and the Hg^{II}-bound (grey) structures predicted from the equidistant MD snapshots between 100 and 2000 ns. The spectrum calculated for the peptide from the AfArsR-As crystal structure (PDB ID: 6J05) is included as a dark red curve for comparison.

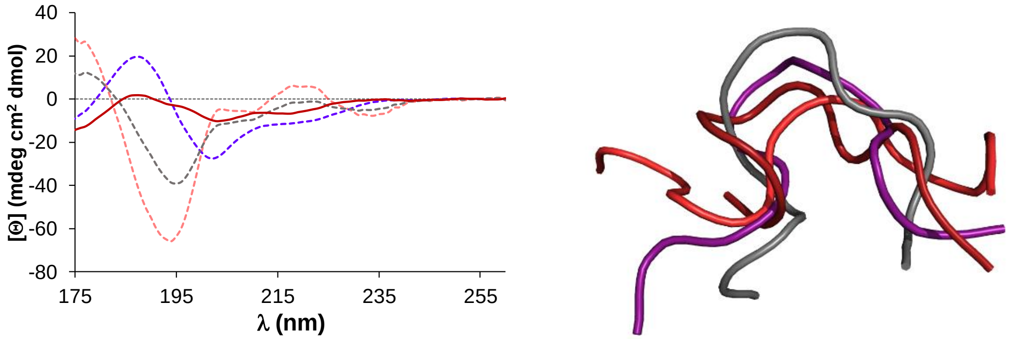

Figure A3. Left panel: Comparison of the predicted circular dichroism spectra of the NCCHGTRDCA peptide in its free form (red); As^{III}-bound form (violet) and Hg^{II}-bound form (grey) from the 2000 ns snapshot of the MD simulations. The spectrum calculated for the peptide from the AfArsR-As crystal structure (PDB ID: 6J05) is included as a dark red curve for comparison. Right panel: Cartoons of the peptide structures related to the CD spectra shown in the left panel using the same color code.

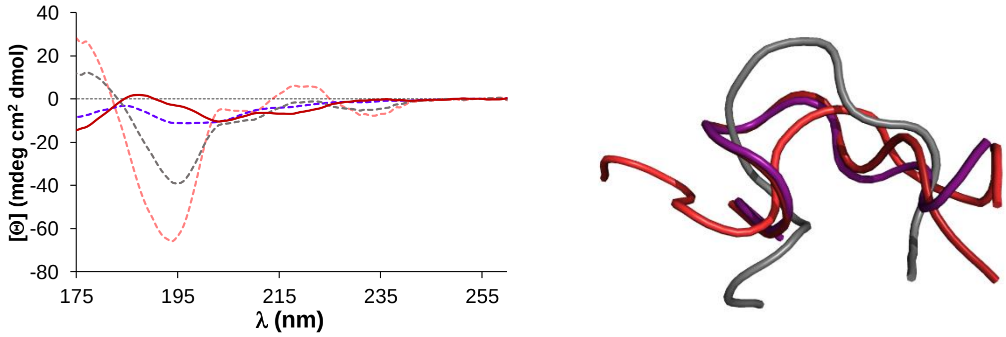

Figure A4. Left panel: Comparison of the predicted circular dichroism spectra of the NCCHGTRDCA peptide in its free form (red); As^{III}-bound form (violet) and Hg^{II}-bound form (grey). The 2000 ns snapshot of the MD simulations was used for calculations, with the exception of the As^{III}-bound form. The spectrum calculated for the peptide from the AfArsR-As crystal structure (PDB ID: 6J05) is included as a dark red curve for comparison. Right panel: Cartoons of the peptide structures related to the CD spectra shown in the left panel using the same color code.

In view of the limitations of the CD prediction algorithms, we cannot consider the above results as a definite proof of the suggested peptide structures. Nevertheless, the same trends in the intensity ranges of the predicted and experimental CD spectra suggest that it is not impossible to correlate the intensity changes of the CD spectra to the peptide backbone structural changes.

References

(A1) Nagy, G.; Igaev, M.; Hoffmann, S. V.; Jones, N. C.; Grubmueller, H. SESCA: Predicting Circular Dichroism Spectra from Protein Molecular Structures. *J. Chem. Theory Comput.* **2019**, *15*, 5087–5102.

(A2) Drew, E. D.; Janes, R. W. PDBMD2CD: Providing Predicted Protein Circular Dichroism Spectra from Multiple Molecular Dynamics-Generated Protein Structures. Nucl. Acids Res. 2020, 48, W17–W24.

(A3) Nagy, G.; Oostenbrink, C. Dihedral-Based Segment Identification and Classification of Biopolymers I: Proteins. *J. Chem. Inf. Model.* **2014**, *54*, 266−277.

Prediction of CD spectral properties using time-dependent density functional theory (TD-DFT) of the As^{III} bound NCCHGTRDCA peptide

We attempted to utilize a quantum mechanical formalism to understand the electronic transitions of the decapeptide fragment analyzed in MD simlulations in the As^{III} bound form. A snapshot from the MD calculations was used in the zwitterionic form as the starting point for a geomtetry optimization at the B3LYP DEF2-SVP D4 level with using Orca 5.0.4 specifying tight optimization and SCF convergence criteria.

Using this optimized geometry a time-dependent DFT calculation was performed using B3LYP with the V2.5 D4 dispersion correction and the DEF2-TZVP basis set on the system of 138 atoms with 622 electrons requesting 150 excited states. The standard TD-DFT procedure in Orca was used which utilizes the Tamm-Dancoff approach.

The TD-DFT calculation output was visualized using Avogadro v. 1.2.0 to show the predicted oscillator and rotational strengths in the velocity dipole formalism (Figure A5).

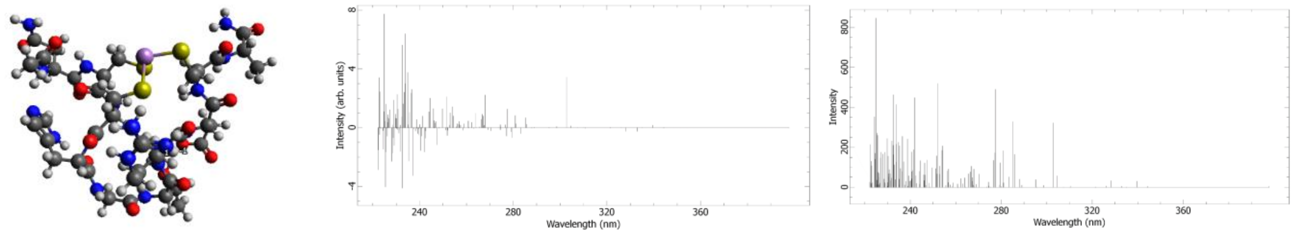

Figure A5. Converged geometry (left), the predicted intensity of excitation (center), and the rotational strength (right) with y-axes in arbitrary units.

An analysis of the predicted excited states showed that nearly all the transitions predicted to have a significant rotational strength had participation of As centered orbitals. Pure peptide backbone centered excitations were not found in the energy range predicted for the requested 150 excited states as the highest predicted excitation energy corresponded to 222 nm. Out of the 150 predicted excited states the 50 with highest energy fell in the range from 235 nm to 222 nm. As a further increase in the density of states is expected for higher excitation

energies, it is very challenging to computationally approach the far-UV range where peptide backbone amide transitions are observed experimentally.

Optimized cartesian coordinates NCCHGTRDCA peptide B3LYP DEF2-SVP D4:

C	5.248678	-5.125241	2.453025
C	3.972387	-5.229043	3.256195
O	3.822228	-4.584749	4.307794
H	6.095249	-4.977985	3.137205
H	5.156530	-4.231408	1.814783
H	5.422552	-6.001632	1.813098
N	3.021113	-6.026629	2.734503
C	1.636672	-6.099523	3.206405
C	0.900397	-4.826422	2.756334
O	0.066449	-4.816721	1.864969
C	1.483831	-6.389108	4.709646
C	2.351265	-7.579883	5.111788
O	2.036144	-8.724923	4.844263
N	3.493092	-7.241970	5.774902
H	3.147177	-6.357844	1.761072
H	1.176155	-6.931957	2.659668
H	0.435229	-6.665639	4.892881
H	1.722043	-5.504743	5.310782
H	4.160677	-7.981706	5.962053
H	3.814203	-6.278241	5.770178
N	1.309388	-3.691343	3.403427
C	1.001807	-2.410054	2.829788
C	1.914699	-2.126625	1.622665
O	2.963276	-2.714653	1.428734
C	1.157650	-1.342722	3.930258
S	0.613505	0.355419	3.465457
H	2.182098	-3.774491	3.940932
H	-0.039864	-2.431087	2.473529
H	2.203730	-1.304401	4.275848
H	0.541285	-1.647760	4.787626
N	1.474006	-1.127125	0.788076
C	2.412780	-0.445960	-0.083283

C	2.822075	-1.252229	-1.323277
O	3.802900	-0.918578	-1.999228
C	3.594553	0.190504	0.655816
S	3.154739	1.850766	1.322498
H	0.727541	-0.544424	1.164422
H	1.845924	0.380776	-0.535938
H	4.419042	0.350210	-0.048130
H	3.975061	-0.472530	1.444821
N	1.942027	-2.182369	-1.720451
C	1.984670	-2.813298	-3.030695
C	0.520754	-2.993644	-3.463671
O	-0.253396	-3.671280	-2.789012
C	2.793845	-4.134997	-3.044209
C	2.575411	-5.035394	-1.873537
N	1.340626	-5.503762	-1.474191
C	3.469843	-5.587059	-0.981536
C	1.529355	-6.287654	-0.386515
N	2.807758	-6.368191	-0.065695
H	1.206274	-2.464705	-1.077078
H	2.490599	-2.109484	-3.707121
H	3.860201	-3.866801	-3.058172
H	2.584933	-4.649734	-3.999866
H	0.457347	-5.158879	-1.857791
H	4.551228	-5.458502	-0.958897
H	0.700980	-6.732002	0.160505
N	0.151025	-2.349148	-4.587020
C	-1.236915	-2.223186	-5.002711
C	-1.541141	-0.722875	-5.104881
O	-0.691338	0.023668	-5.587178
H	0.776922	-1.674597	-5.020322
H	-1.866788	-2.747072	-4.272172
H	-1.389519	-2.688322	-5.992032
N	-2.720626	-0.319759	-4.610892
C	-3.064995	1.079040	-4.393560
C	-2.063163	1.778103	-3.465377
O	-1.712276	2.932558	-3.639954

C	-4.498542	1.084948	-3.778122
O	-4.559683	0.034000	-2.844403
C	-5.569503	0.908563	-4.850846
H	-3.305740	-0.953808	-4.062312
H	-3.031039	1.644975	-5.334650
H	-4.660744	2.054318	-3.276236
H	-4.663876	0.448830	-1.918290
H	-5.546827	1.734302	-5.580261
H	-5.434324	-0.042382	-5.392446
H	-6.556932	0.893577	-4.366865
N	-1.585600	1.008297	-2.439074
C	-0.566614	1.489341	-1.527102
C	-1.042114	2.603964	-0.559775
O	-0.281529	3.515280	-0.235714
C	0.691687	2.016799	-2.224254
C	1.257794	1.174098	-3.355553
C	2.592920	1.735618	-3.845578
N	3.646507	1.714376	-2.824432
C	3.990192	2.712571	-2.021739
N	3.290624	3.854797	-1.952144
N	5.120931	2.606136	-1.285245
H	-2.107310	0.167393	-2.214369
H	-0.293445	0.618550	-0.904418
H	1.424834	2.152407	-1.416838
H	0.448286	3.016784	-2.613457
H	0.565147	1.154213	-4.209059
H	1.383915	0.123247	-3.048679
H	2.470420	2.771622	-4.197901
H	2.968916	1.152406	-4.699984
H	3.981379	0.770244	-2.568248
H	3.649497	4.622953	-1.398265
H	2.288742	3.850381	-2.124234
H	5.136273	3.012106	-0.353132
H	5.722924	1.805566	-1.438530
N	-2.292673	2.467944	-0.097753
C	-3.014991	3.592036	0.485400

C	-2.320798	4.118307	1.742385
O	-2.255717	5.300991	2.018549
C	-4.479745	3.163990	0.743495
C	-5.032460	2.393844	-0.484891
O	-4.506095	1.216237	-0.606912
O	-5.817249	2.931257	-1.254801
H	-2.939080	1.715843	-0.427459
H	-3.008212	4.440006	-0.219064
H	-5.083213	4.060364	0.936512
H	-4.519710	2.499376	1.622036
N	-1.819358	3.138844	2.573579
C	-0.797020	3.453729	3.554937
C	-1.233872	4.557098	4.535600
O	-0.440469	5.387257	4.938110
C	0.545979	3.822554	2.904543
S	1.979969	3.520059	4.024729
H	-1.781641	2.207637	2.169772
H	-0.661066	2.548778	4.166453
H	0.577882	4.897057	2.683286
H	0.687738	3.283409	1.960162
N	-2.524033	4.448993	4.971526
C	-3.191882	5.563623	5.634999
C	-3.776328	5.184257	7.008222
O	-4.619495	5.875234	7.550418
C	-4.249766	6.200538	4.735491
H	-3.115463	3.832992	4.420808
H	-2.396019	6.297374	5.846918
H	-5.035107	5.468925	4.478821
H	-4.732881	7.033145	5.263782
H	-3.787732	6.556213	3.804032
N	-3.259220	4.057540	7.567938
H	-2.537394	3.530399	7.096326
H	-3.550087	3.806640	8.504627
As	2.650138	1.440057	3.539018
